# Combined nicotinamide N-methyltransferase inhibition and reduced-calorie diet normalizes body composition and enhances metabolic benefits in obese mice

**DOI:** 10.1038/s41598-021-85051-6

**Published:** 2021-03-11

**Authors:** Catherine M. Sampson, Andrea L. Dimet, Harshini Neelakantan, Kehinde O. Ogunseye, Heather L. Stevenson, Jonathan D. Hommel, Stanley J. Watowich

**Affiliations:** 1 Department of Pharmacology and Toxicology, University of Texas Medical Branch At Galveston, Galveston, TX USA; 2 Center for Addiction Research, University of Texas Medical Branch, Galveston, TX USA; 3 Department of Biochemistry and Molecular Biology, University of Texas Medical Branch at Galveston, Galveston, TX USA; 4 Ridgeline Therapeutics, Houston, TX USA; 5 Institute for Translational Sciences, University of Texas Medical Branch, Galveston, TX USA; 6 Department of Pathology, University of Texas Medical Branch at Galveston, Galveston, TX USA

**Keywords:** Target validation, Obesity

## Abstract

Obesity is a large and growing global health problem with few effective therapies. The present study investigated metabolic and physiological benefits of nicotinamide N-methyltransferase inhibitor (NNMTi) treatment combined with a lean diet substitution in diet-induced obese mice. NNMTi treatment combined with lean diet substitution accelerated and improved body weight and fat loss, increased whole-body lean mass to body weight ratio, reduced liver and epididymal white adipose tissue weights, decreased liver adiposity, and improved hepatic steatosis, relative to a lean diet substitution alone. Importantly, combined lean diet and NNMTi treatment normalized body composition and liver adiposity parameters to levels observed in age-matched lean diet control mice. NNMTi treatment produced a unique metabolomic signature in adipose tissue, with predominant increases in ketogenic amino acid abundance and alterations to metabolites linked to energy metabolic pathways. Taken together, NNMTi treatment’s modulation of body weight, adiposity, liver physiology, and the adipose tissue metabolome strongly support it as a promising therapeutic for obesity and obesity-driven comorbidities.

## Introduction

The ever-increasing global incidence of obesity, a disorder characterized by excessive whole-body adiposity, is a major wellness, healthcare, and economic concern. Among US adults, the age-adjusted prevalence of obesity (defined as a body mass index [BMI] ≥ 30 kg/m^2^) has increased by 12.1% over the past two decades^1^. The associated upsurge in obesity-driven comorbidities, including Type 2 Diabetes (T2D), cardiovascular disease, cancer, and non-alcoholic steatohepatitis (NASH) has elevated obesity to the third leading cause of preventable death in the US^2–5^. Lifestyle modifications (e.g., low-calorie diets) and behavioral therapies remain the standard-of-care for obesity treatment^6,7^, even though 40–65% of individuals are unable to sustain ≥ 5% body weight loss for two years after beginning a comprehensive lifestyle intervention^7^. While weight loss benefits associated with lifestyle modification can generally be improved with adjunct pharmacotherapies^6^, the medications currently approved to treat obesity have only modest efficacy and suffer numerous side effects^8^. Thus, the quest for safe and effective pharmacotherapies to treat obesity continues.

The multifactorial nature of obesity and its related metabolic dysfunctions pose great challenges while offering diverse targets for developing effective therapeutics. Several drug classes with different mechanisms of action to treat obesity are in preclinical-to-late-stage clinical testing, including centrally- and peripherally-acting anorexigenic agents, lipase inhibitors, and adipose tissue-targeted modulators of lipid metabolism, inflammatory signaling, and angiogenesis^9^. Nicotinamide N-methyltransferase (NNMT), an enzyme highly expressed in white adipose tissue (WAT)^10^, is a recently discovered drug target that has been preclinically validated^11–13^ and clinically evaluated^14,15^ for the management of obesity and T2D. Located at the nexus of the nicotinamide adenine dinucleotide (NAD) salvage and methionine metabolic pathways, NNMT serves as a critical regulator of cellular energy metabolism and epigenetic alterations linked to adiposity and insulin sensitivity^11,16–18^. NNMT expression has been proposed to modulate metabolic dysfunction in key nutrient-metabolizing and energetically active tissues (e.g., adipose, liver, skeletal muscle)^16,19^ linked to obesity, fatty liver disease, and muscle aging^11,12,20,21^. NNMT activity is dramatically increased in the WAT of diet-induced obese (DIO) mice^22^, and its mRNA expression is increased in both the omental and subcutaneous adipose tissues of individuals with T2D^14^. Conversely, NNMT mRNA levels significantly decline in WAT (i.e., visceral fat) following bariatric surgical intervention in obese individuals and in subcutaneous fat following increased exercise in individuals with impaired glucose tolerance or T2D^14^.

We previously characterized a novel NNMT inhibitor (NNMTi), 5-amino-1-methylquinolinium, that decreases lipogenesis in adipocytes and protects animals against diet-induced body weight and fat gain^12^. 5-amino-1-methylquinolinium is an NNMT-selective probe small molecule inhibitor compound with an IC_50_ ~ 1µM^23^; this NNMTi displays favorable drug-like characteristics including high aqueous solubility, amenable passive permeability and active transport, target engagement both in vitro (e.g., adipocytes) and in vivo (e.g., murine adipose tissue), cross-species liver metabolic stability, and substantial systemic exposure and pharmacokinetic profile^12,23^; the latter three are known in part from unpublished work.. While systemic NNMTi treatment dramatically limits EWAT weight increases in DIO mice^12^, the WAT signaling pathways and metabolomic alterations that are associated with NNMTi treatment-mediated protection from an obesogenic phenotype are largely unknown. Moreover, since few studies have systematically analyzed metabolomic changes within the WAT following prolonged exposure to high-fat diet (HFD)^24,25^, it is currently unknown if a low-calorie diet, either alone or combined with pharmacotherapy, can return the WAT metabolome to a healthy (i.e., lean) state following chronic HFD dysregulation. In the comprehensive study described herein, we analyze the effect of NNMTi treatment combined with a reduced-calorie diet (lean diet, LD) on body weight, body composition, obesity-associated liver pathologies, and the WAT metabolomic profile in a mouse model of diet-induced obesity.

## Results

### NNMTi treatment combined with LD switch produced rapid and sizable body weight and fat mass losses in obese mice

**Figure 1:**
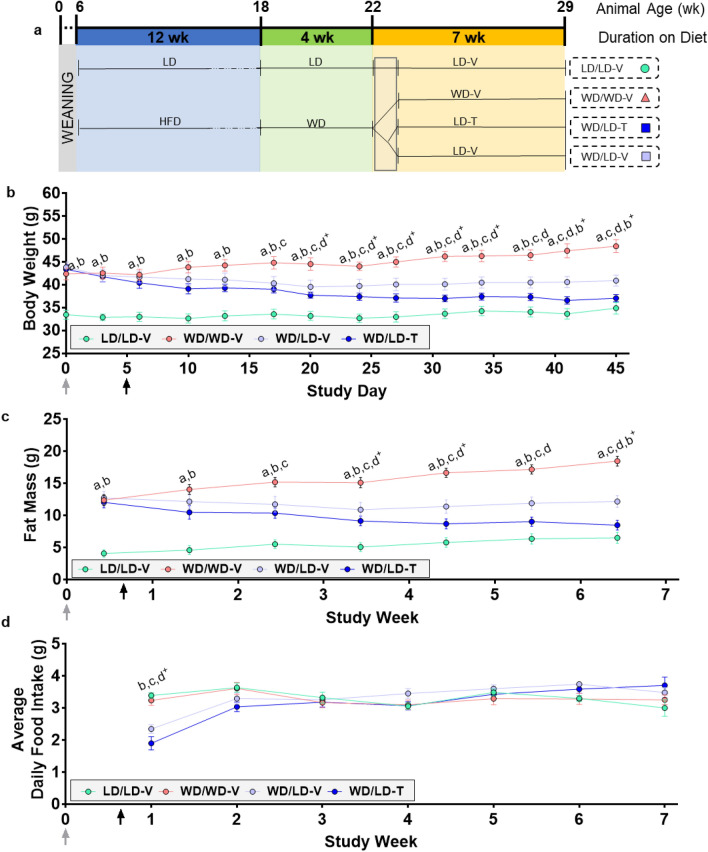
NNMTi treatment combined with a lean diet switch accelerated weight and fat loss, promoting persistent weight and fat reductions without impacting food intake. Study timeline (**a**), body weight (**b**), fat mass (**c**), and average daily food intake (**d**) across the study period (n = 6–8/group); weights are in grams and age is in weeks (wk). Data are represented as mean ± SEM; treatment start is labeled by the black arrow and diet change by the grey arrow; the grey box represents the 3-day transition to diet followed by 2 days of sham injections. Body weight analyses demonstrated a significant main effect of treatment group (p < 0.0001), study day (p < 0.0001), and an interaction of treatment group by study day (p < 0.0001), rendering the NNMTi-treated group indistinguishable from the LD control group upon study completion. Fat mass demonstrated main effects of treatment group (p < 0.0001), study week (p < 0.0001), and a study week by treatment group interaction (p < 0.0001). Average daily food intake only demonstrated a significant effects of study week (p < 0.0001) and a study week by treatment group interaction (p < 0.0001). Mixed effects models (**b**,**d**); two-way repeated measures ANOVA (**c**). For a full list of statistical results, see Supplementary Table S1. *LD* lean diet, *HFD* high-fat diet, *-T* NNMTi-treated, *-V* vehicle-treated, *WD* Western diet; a, LD/LD-V vs. WD/WD-V; b, both WD/LD groups vs. LD/LD-V; c, both WD/LD groups vs. WD/WD-V; d, WD/LD-T vs. WD/LD-V. Labeled pairwise comparisons are significant (< 0.05) corrected (q) and uncorrected (p) for multiple comparisons; ‘ + ’, significant result with FDR correction only.

Twenty-two-week old obese mice (previously maintained on the high-fat "Western" diet [WD]) were switched to LD, acclimated for 3 days, and then randomized into vehicle control (WD/LD-V) and NNMTi (WD/LD-T) treatment arms alongside LD and WD controls (described schematically in Fig. 1a). Reduced calorie intake with the LD switch resulted in an initial body weight loss (average 4.3 g by day 20) that plateaued with a slight weight regain thereafter in WD/LD-V mice (cumulative body weight loss averaged 2.9 g by day 45; Fig. 1b). The changes in body weight in the WD/LD-V group were paralleled by a reduction in fat mass during the initial 3.5 weeks following switch to LD and then a continual regain of fat mass to the end of the study (cumulative fat mass loss in the WD/LD-V group, 0.55 g; Fig. 1c). In contrast, WD/LD-T mice exhibited accelerated body weight and fat loss that robustly persisted through the study (cumulative body weight loss of 6.3 g at study end) that was statistically significant different compared to LD switch alone and nearly indistinguishable from the LD/LD-V group by body weight and whole-body fat mass levels (Fig. 1b,c; Supplementary Table S1). The percent change in fat mass from baseline was ten-fold higher with NNMTi treatment (~ 29.3% fat mass loss), relative to LD switch alone (2.9% fat mass loss), and the body weight loss, measured as percent from baseline, was more than double with NNMTi treatment relative to LD switch alone (Supplementary Fig. S1). Unexpectedly, LD/LD-V control mice displayed a shift towards increased fat mass, i.e., an average net whole-body fat mass gain of 2.4 g despite maintaining relatively stable body weight throughout the 7-week study (cumulative body weight gain, 1.4 g; Fig. 1b,c). This is attributed, in part, to the long-term maintenance on the LD that has substantially higher carbohydrate composition. In contrast, WD/WD-V mice demonstrated a classic obesogenic phenotype, with continual body weight (cumulative body weight gain, 6.0 g) and fat mass gain—whole-body fat mass was nearly threefold higher than the LD/LD-V controls at the end of the study (Fig. 1b, c; Supplementary Table S1). The net fat mass change across all experimental groups positively correlated with the net body weight change (Supplementary Fig. S2; Supplementary Table S3), suggesting a tight correlation between these measures and their responsiveness to dietary and/or treatment interventions.

NNMTi treatment combined with a reduced-calorie diet produced sizeable, persistent, and cumulative increases in the ratio of whole-body lean mass to body weight from baseline, which was significantly different compared to the vehicle-treated counterparts throughout the study (Supplementary Fig. S1; Supplementary Table S1). In the WD/LD-T group, the ratio of whole-body lean mass to body weight increased by 6.4% from baseline at the study termination. Importantly, despite causing modest reductions in body fat levels, reducing calorie intake with only the LD switch did not change lean mass to body weight ratios from baseline. Mice maintained on WD throughout the study showed continual declines in their whole-body lean mass to body weight ratio, suggesting obese mice gain fat mass without corresponding increases in lean mass when continuously maintained on WD. Surprisingly, the LD/LD-V mice showed similar longitudinal decreases in their ratio of whole-body lean mass to body weight, supporting an overall shift toward higher fat-to-lean mass ratio, which may be supported in part by the long-term maintenance on an unrestricted, high-carbohydrate lean diet.

Average daily food intake did not significantly differ by treatment group but differed by study week and had a significant interaction of study week by treatment group. Mice transitioned from WD to LD exhibited significant decreases in average daily food intake for the first week after the diet switch relative to WD/WD-V mice; this decrease resolved after week 2 (Fig. 1d). This suggested that initial differences in average daily food intake were driven mainly by LD switch, and likely related to differences in food neophobia^27^ and/or palatability issues arising through the acclimatization phase after the LD switch.

**Figure 2:**
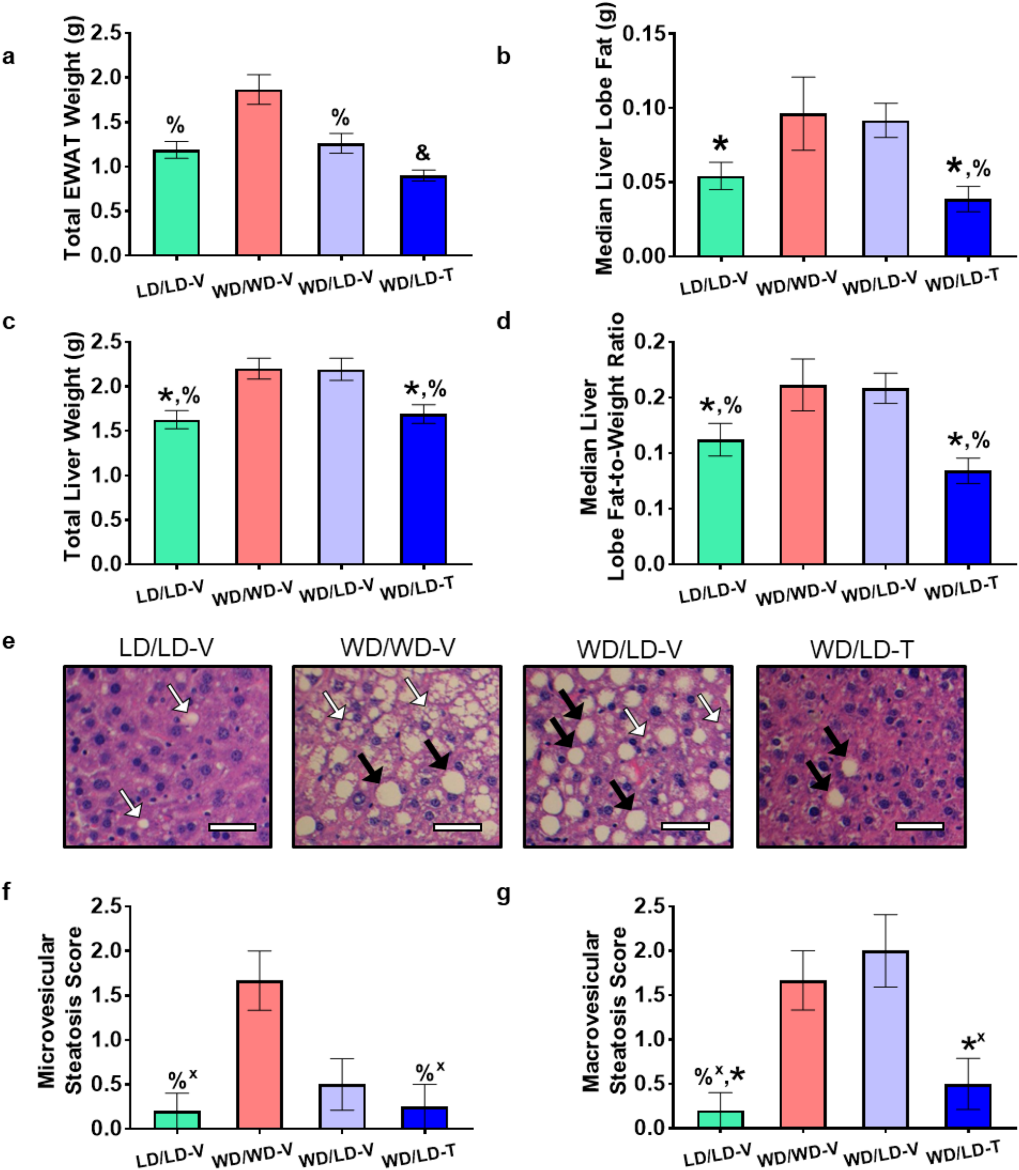
NNMTi treatment combined with a lean diet (LD) switch returned liver adiposity, liver and EWAT weights, and microvesicular and macrovesicular steatosis from pathological values associated in an obese state to values observed in mice continually maintained on LD. Total EWAT weight (**a**), median liver lobe fat content (**b**), total liver weight (**c**), median liver lobe fat-to-weight ratio (**d**), representative histology showing microvesicular steatosis (white arrows) and macrovesicular steatosis (black arrows) (**e**, scale bar 25 µm), and microvesicular (**f**) and macrovesicular (**g**) steatosis scores. Data are represented as mean ± SEM; **a-d** n = 6–8/group and weights are in grams; **f-g**, n = 3–5/group. Significant main effects for treatment group were observed for total EWAT weight (p < 0.0001), median liver lobe fat (p = 0.0034), total liver weight (p = 0.0011), median liver lobe fat-to-weight ratio (p = 0.0045), microvesicular (p = 0.0425) and macrovesicular (p = 0.0039) steatosis scores. One-way ANOVA on log transformed data (**a,b**) ; one-way ANOVA (**c,d**); Kruskal–Wallis test (**f,g**). For a full list of statistical results, see Supplementary Table S2. *LD* lean diet, *-T* NNMTi-treated, *-V* vehicle-treated, *WD* Western diet, &, vs. all other groups displayed; *, vs. WD/LD-V; %, vs. WD/WD-V. Labeled pairwise comparisons are significant (< 0.05) corrected (q) and uncorrected (p) for multiple comparisons; ‘ + ’, significant result with FDR correction only.

The EWAT is one of two primary sources of WAT in the mouse^28^. Gross EWAT weight (combined left and right lobes) differed significantly across treatment groups (Fig. 2a; Supplementary Table S2); as expected, LD/LD-V mice had significantly lower EWAT weights relative to WD/WD-V mice. Moreover, EWAT weight was significantly lower in WD/LD-V mice compared to WD/WD-V mice, suggesting a preferential visceral fat loss mediated by reduced calorie intake. In the WD/LD-T group, EWAT weights were dramatically lower compared to all other groups, significantly lower than the reductions observed with LD switch alone; the changes in EWAT weight were maintained when total EWAT weight was normalized to terminal body weight (Supplementary Table S2). Consistent with the unexpected whole-body fat mass gain noted in the LD/LD-V control group, total EWAT weight accounted for a larger proportion of the whole-body adiposity compared to all other groups, (Supplementary Fig. S2; Supplementary Table S2). Specifically, gross EWAT weight accounted for 18.7% of body fat in the LD/LD-V mice, a near doubling compared to the other groups, suggesting a preference of storage in the EWAT, that may be mediated, in part, by the long-term, high-carbohydrate diet composition. The NNMTi-mediated reduction in total EWAT weight (Fig. 2a) to below that of all other groups, including the LD control group, may suggest an NNMTi-mediated preferential loss of fat in the EWAT, which could portend preferential loss of visceral fat in humans.

Liver fat content was significantly different between the treatment groups (Fig. 2b,d; Supplementary Table S2). Notably, liver adiposity levels in the median lobe with lean diet switch alone were no different than the liver adiposity measures in obese WD/WD-V mice. In contrast, liver adiposity levels and fat-to-weight ratios in the median lobe of the combined LD switch/NNMTi treatment group (WD/LD-T) were significantly lower than the levels observed in the obese WD/WD-V mice and LD switch alone WD/LD-V group (reduced by more than 45%), and comparable to the LD/LD-V controls. Similar differences were observed across groups when analyzing gross whole liver weights (Fig. 2c; Supplementary Table S2), although the overall effect of treatment group was lost when whole liver weights were normalized to body weights (Supplementary Table S2). Importantly, the relative hepatic fat content of the median lobe of WD/LD-T mice was identical to aged-matched LD/LD-V control mice, whereas the relative hepatic fat content of WD/LD-V mice was identical to that of obesogenic WD/WD-V mice (Fig. 2b,d; Supplementary Fig. S2). Taken together, the data suggest that NNMTi treatment combined with LD switch can achieve extensive and restorative adiposity changes in the EWAT and liver of previously obese mice; these reservoirs collectively correspond to > 10% of the whole-body fat. In contrast, LD switch alone was insufficient to lower the EWAT and liver adiposity levels of previously obese mice, suggesting that pathogenic adiposity levels that develop with diet-induced obesity are difficult to reverse by subsequent calorie restriction.

### NNMTi treatment combined with LD switch improved liver steatosis and did not exhibit serum markers of organ damage

**Figure 3:**
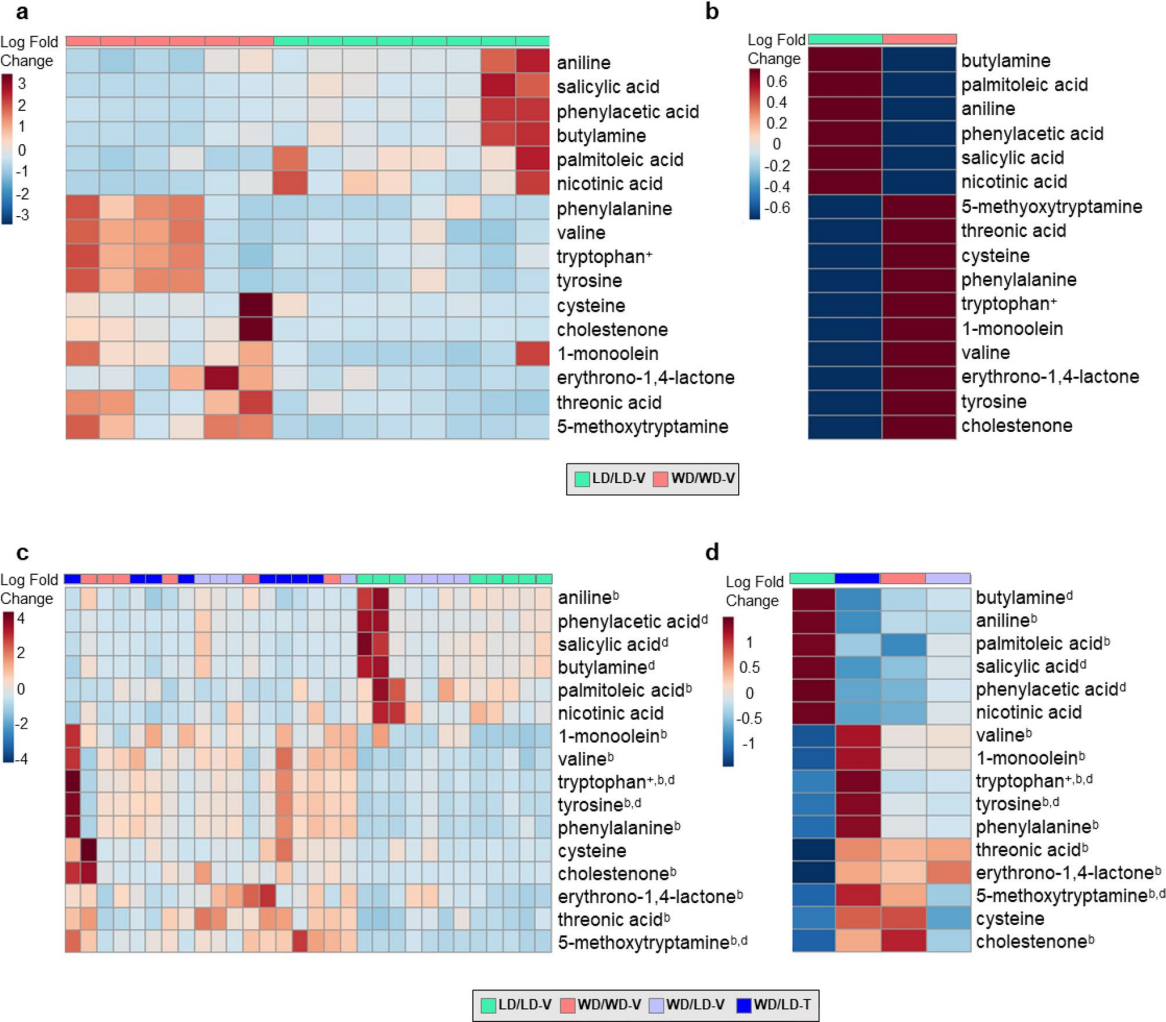
LD Diet switch alone and NNMTi treatment combined with LD switch influence the obesogenic diet-induced EWAT metabolic signature. Heat maps of the sixteen metabolites that demonstrated a significant pairwise comparison between control mice maintained on WD vs. LD; individual mouse results (**a**) and group averages (**b**) are displayed for these in the top panels, and the bottom panels include the addition of the individual mouse results (**c**) and group averages (**d**) for the WD/LD-V and WD/LD-T groups (n = 6–8/group for all panels). Metabolites were analyzed raw or log transformed, and using a one-way ANOVA, Welch’s ANOVA, or Kruskal–Wallis test. For a full list of statistical results, see Supplementary Table S5. *LD* lean diet, *-T* NNMTi-treated, *-V* vehicle-treated, *WD* Western diet, *b* both WD/LD groups vs. LD/LD-V, *d* WD/LD-T vs. WD/LD-V. Labeled pairwise comparisons are significant (< 0.05) corrected (q) and uncorrected (p) for multiple comparisons; ^+^significant result with FDR correction only.

Since liver adiposity was dramatically reduced in WD/LD-T mice, we examined the impact of NNMTi treatment on liver pathologies associated with obesity, particularly the steatosis phenotype defining NAFLD (non-alcoholic fatty liver disease) and NASH (non-alcoholic steatohepatitis). Hepatic microvesicular and macrovesicular steatosis scores (illustrated by representative histological images from sectioned left lateral lobes; Fig. 2e) differed significantly across treatment groups (Fig. 2f-g; Supplementary Table S2). Mice in the WD/WD-V group had numerous small and large cytoplasmic lipid vacuoles within hepatocytes, the defining morphological features of microvesicular and macrovesicular steatosis, respectively. The high micro- and macrovesicular steatosis scores observed in WD/WD-V mice were comparable to those observed in WD/LD-V mice. In contrast, and consistent with the liver adiposity reductions, the NNMTi treatment group (WD/LD-T) had significantly lowered microvesicular and macrovesicular steatosis scores compared to WD/WD-V controls (results significant without an FDR correction only); the levels of steatosis in WD/LD-T mice were comparable to the steatosis levels observed in LD/LD-V control mice (Fig. 3b,c). Consistently, microvesicular and macrovesicular steatosis scores positively correlated with liver fat content as measured by MRI scans (Supplementary Table S3). As expected for DIO models^29^, particularly given the duration of energy-rich diet exposure^30^, hepatic fibrosis was not observed in any animal. Additional NAFLD-specific phenotypes, such as inflammation and hepatocellular ballooning^31^, did not differ significantly between treatment groups in the samples analyzed, although there was a slight trend toward improved inflammation scores in the WD/LD-T group relative to the WD/WD-V group (Supplementary Table S2).

Of note, repeated dosing of WD/LD-T mice with NNMTi over the 7-week period did not produce any adverse effects and was well tolerated. This was further confirmed by the fact that serum chemistry markers were not significantly different between groups (for a full list of results, see Supplementary Table S4), except total protein concentration and albumin that were significantly higher in the WD/LD-V group compared to all other groups; albumin likely drove the total protein concentration changes^32^.

### NNMTi treatment combined with LD switch displays a unique EWAT metabolomic signature

**Figure 4:**
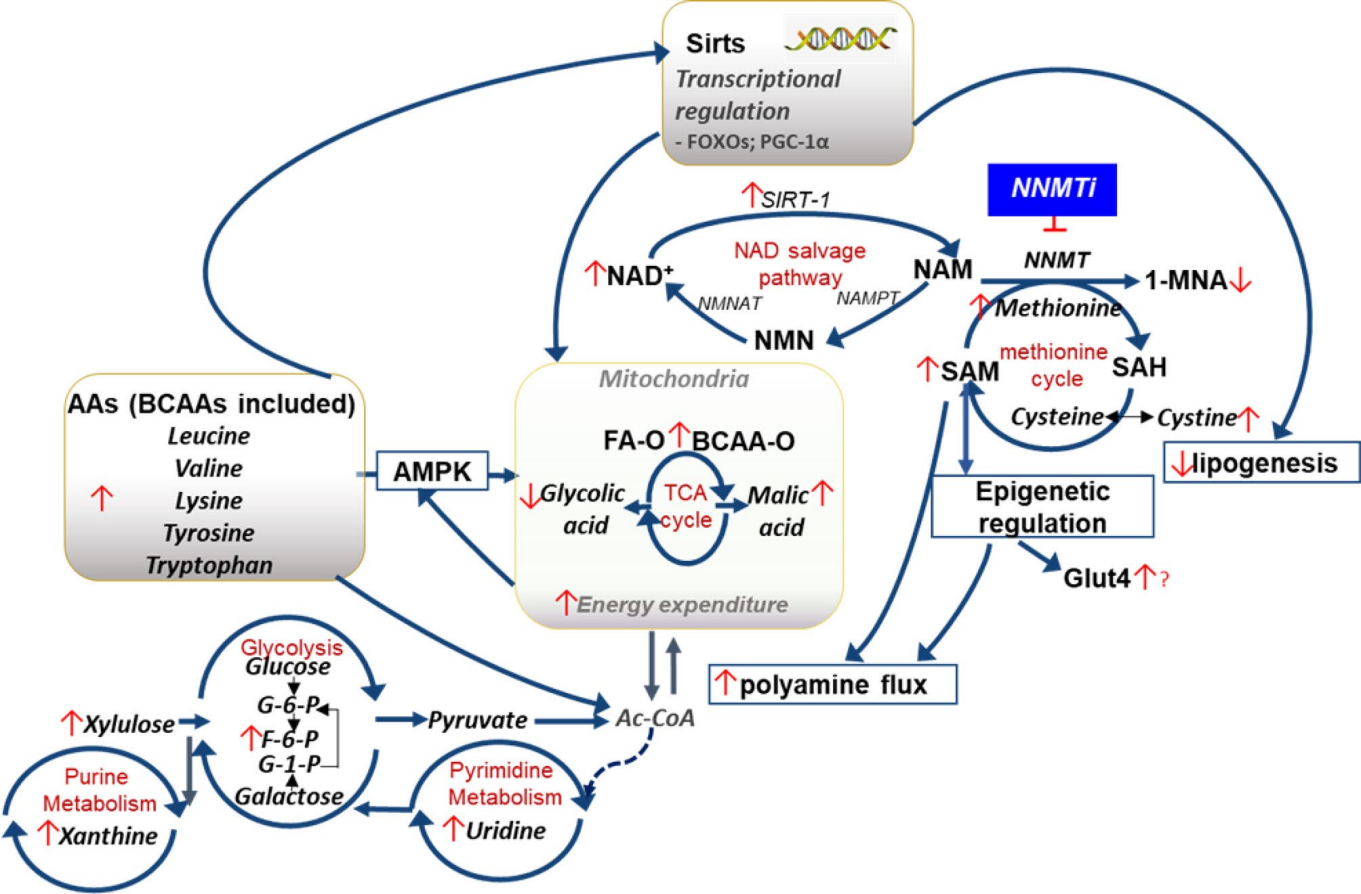
Impact of NNMTi treatment combined with LD switch on EWAT metabolome and associated pathways regulated in DIO mice. Schematic illustration of pathways modulated by NNMTi treatment independently or in conjunction with diet switch**.** *1-MNA* 1-methylnicotinamide, *AA* amino acid, *Ac-CoA* acetyl-coenzyme A, *AMPK* adenosine monophosphate kinase, *BCAA* branched-chain amino acid, *F-6-P* fructose-6-phosphate, *FA* fatty acid, *FOXO* forkhead box protein O, *Glut4* glucose transporter type 4, *G-1-P* glucose-1-phosphate, *G-6-P* glucose-6-phosphate, *NAD* nicotinamide adenine dinucleotide, *NAM* nicotinamide, *NAMPT* nicotinamide phosphoribosyltransferase, *NF-κB* nuclear factor-kappa B, *NMN* nicotinamide mononucleoside, *NMNAT* nicotinamide adenylyltransferase, *NNMT* nicotinamide N-methyltransferase, *-O* oxidation, *PGC-1 α* peroxisome proliferator-activated receptor (PPAR) gamma coactivator 1-alpha, *SAH* S-adenosyl-L-homocysteine, *SAM* S-adenosyl-L-methionine, *Sirts* sirtuins, *SIRT1* (NAD-dependent deacetylase) sirtuin 1, *TCA* tricarboxylic acid.

Given the dramatic impact on NNMTi treatment combined with LD switch had on whole-body adiposity, EWAT metabolomic analyses were performed to identify the molecular pathways modulated by this intervention. Of 170 metabolites measured in EWAT, thirty were significantly altered by treatment group (Fig. 3; Table 1; Supplementary Table S5). Our results evinced a clear obese EWAT metabolomic profile in WD/WD-V mice compared to lean LD/LD-V mice, with sixteen EWAT metabolites demonstrating significant differences (Fig. 3a,b; Table 1; Supplementary Table S5). The distinct WD/WD-V and LD/LD-V EWAT metabolomic signatures were used as baseline standards to evaluate the impact of calorie reduction (LD switch), either alone or combined with NNMTi treatment, on restoration or modulation of the EWAT metabolome (Fig. 3c,d). Contrary to our expectations, NNMTi treatment did not simply shift the EWAT metabolomic profile from the obese state profile to the lean state profile (Table 1), but instead produced a unique EWAT metabolomic signature (Fig. 3c,d). Metabolomic changes in the WD/LD-T group relative to those metabolomic changes observed with LD switch alone were mostly overlapping, but some effects were augmented or reversed by NNMTi treatment. Contrary to our expectations, we only observed three metabolites that were modified in the WD/LD-V group relative to the WD/WD-V group differently than the WD/LD-T group relative to the WD/LD-V group: 5-methoxytryptamine, cystine, and phenylacetic acid (Table 1). Surprisingly, however, for 5-methoxytryptamine and phenylacetic acid the diet switch alone had brought the abundance closer to that of the LD control and the NNMTi treatment restored the abundance to the WD control levels (only a trend for 5-methoxytryptamine). For cystine, diet switch increased the relative abundance of cystine beyond that of both the LD and WD control groups, and NNMTi treatment exacerbated this effect. There were also only three metabolites modified in the WD/LD-V group relative to the LDL/LD-V group differently than the WD/LD-T group relative to the WD/LD-V group: xylulose, tyrosine and tryptophan (Table 1). Each of these were increased with diet switch, and further increased with NNMTi treatment. Collectively, the metabolites where NNMTi treatment augmented or reversed the impact of diet switch relative to a control group were upstream of glycolysis, downstream of the methionine cycle, or amino acids or their derivatives (Fig. 4).

**Table 1:** Epididymal white adipose tissue metabolome.

Metabolite name	LD/LD-V	WD/WD-V	WD/LD-V	WD/LD-T
	(mean ± SD)	(mean ± SD)	(mean ± SD)	(mean ± SD)
1-Monoolein^1,a,b^	**3.73 ± 0.41**	**4.24 ± 0.24**	**4.24 ± 0.25**	**4.42 ± 0.23**
5-Methoxytryptamine^1,a,b,d^	**2.68 ± 0.16**	**3.44 ± 0.27**	**3.11 ± 0.37**	**3.54 ± 0.32**
Aniline^ax,bx^	**1283.13 ± 746.82**	**683.50 ± 309.33**	**685.80 ± 156.52**	**484.40 ± 179.57**
Aspartic acid^dx^	7391.38 ± 4162.76	30,892.17 ± 24,287.27	29,406.90 ± 16,253.93	59,051.50 ± 42,748.03
Benzoic acid	71,280.50 ± 59,311.67	28,965.67 ± 5,349.76	33,954.90 ± 14,962.07	20,891.90 ± 8390.10
Butylamine^ax,dx^	**2887.38 ± 2323.44**	**1146.83 ± 432.85**	**1298.90 ± 644.53**	**617.50 ± 339.61**
Cholestenone^1,a,b^	**1.86 ± 0.36**	**3.20 ± 0.49**	**2.68 ± 0.50**	**3.01 ± 0.50**
Cysteine^1,a^	**2.34 ± 0.59**	**3.07 ± 0.59**	**2.79 ± 0.37**	**3.27 ± 0.43**
Cystine^1,f+,c+,^	2.70 ± 0.10	2.68 ± 0.26	2.92 ± 0.24	3.24 ± 0.26
Erythrono-1,4-lactone^1,a,b^	**2.51 ± 0.09**	**2.76 ± 0.25**	**2.85 ± 0.14**	**2.80 ± 0.19**
Fructose-6-phosphate^1^	3.32 ± 0.13	3.33 ± 0.27	3.49 ± 0.25	3.72 ± 0.26
Glycolic acid^dx^	7090.88 ± 1711.87	5927.17 ± 1515.89	5812.90 ± 1676.06	3674.50 ± 1428.89
Hypoxanthine^bx^	5839.13 ± 2467.30	11,304.83 ± 7272.84	16,410.90 ± 12,690.11	22,452.90 ± 7092.86
Inosine^bx^	7187.50 ± 1469.54	12,198.50 ± 5990.20	23,034.10 ± 18,366.01	25,718.90 ± 13,395.88
Leucine^d^	76,138.63 ± 23,928.64	175,005.50 ± 83,715.30	177,467.90 ± 84,555.93	327,353.40 ± 188,924.60
Lysine^d^	5826.25 ± 2183.29	10,479.83 ± 6329.03	9575.00 ± 3787.36	21,211.90 ± 11,587.18
Malic acid^b^	4684.00 ± 1737.87	7237.17 ± 2195.39	9317.50 ± 3930.47	9012.50 ± 2262.07
Methionine^d^	9269.25 ± 2232.44	23,571.33 ± 12,213.49	21,099.60 ± 9325.22	38,675.60 ± 20,918.42
Nicotinamide-to-nicotinic acid ratio^1,ax,dx^	0.86 ± 0.29	1.22 ± 0.34	1.16 ± 0.25	1.54 ± 0.38
Nicotinic acid^1,a^	**3.08 ± 0.28**	**2.71 ± 0.15**	**2.85 ± 0.18**	**2.61 ± 0.29**
Palmitoleic acid^1,a,b^	**4.19 ± 0.20**	**3.88 ± 0.11**	**4.01 ± 0.16**	**3.97 ± 0.12**
Phenylacetic acid^a,d^	**552.63 ± 540.96**	**133.50 ± 33.06**	**223.10 ± 107.26**	**115.10 ± 32.78**
Phenylalanine^ax,bx^	**18,226.75 ± 8088.40**	**44,509.50 ± 24,384.25**	**42,351.10 ± 21,380.39**	**80,573.40 ± 49,016.87**
Salicylic acid^a,d^	**1935.88 ± 1707.85**	**518.33 ± 165.27**	**801.00 ± 445.89**	**316.50 ± 119.46**
Threonic acid^a,b^	**2765.50 ± 1050.38**	**7609.50 ± 3811.13**	**7910.60 ± 4126.15**	**8273.60 ± 3525.86**
Tryptophan^1,a+,b,d^	**4.02 ± 0.10**	**4.26 ± 0.34**	**4.33 ± 0.19**	**4.61 ± 0.26**
Tyrosine^1,a,b,d^	**4.47 ± 0.13**	**4.80 ± 0.34**	**4.81 ± 0.22**	**5.11 ± 0.25**
Uridine^bx^	499.25 ± 295.68	652.00 ± 471.50	1317.10 ± 1018.05	1969.00 ± 944.81
Valine^1,a,b^	**4.55 ± 0.13**	**4.82 ± 0.25**	**4.85 ± 0.16**	**5.00 ± 0.19**
Xanthine^d^	1443.13 ± 1168.78	3351.33 ± 2797.43	4692.50 ± 2849.65	11,670.30 ± 6587.79
Xylulose^1,b,d^	2.58 ± 0.13	2.67 ± 0.14	2.76 ± 0.12	2.94 ± 0.24

Metabolite abundance (mean ± SD), as measured by gas chromatography time-of-flight mass spectrometry, for those with a significant effect of treatment group. Bolded metabolites comprise the EWAT metabolomic signature observed to be highly modulated in the obesogenic group relative to the lean control (Fig. 3). Statistical results from ANOVA/Kruskal–Wallis tests are labeled with superscripts as follows: 'a' (and bolded), significant metabolite differences between lean and obese animals (LD/LD-V control vs. WD/WD-V control); 'b', significant metabolite differences attributed to a shift from WD to LD relative to the lean control (both WD/LD groups vs. LD/LD-V control); 'c', significant metabolite differences attributed to a shift from WD to LD relative to the obese control (both WD/LD groups vs. WD/WD-V control); and 'd', significant metabolite differences attributed to NNMTi treatment (WD/LD-T group vs. WD/LD-V group). Additionally, the superscript '1′ denotes data analyzed log transformed, with mean and SD reported log transformed accordingly; superscript ‘ + ’ denotes a significant result with FDR correction only; superscript ‘x’ denotes a significant result without FDR correction only. n = 6–8 per group. Ranges for each metabolite and additional details on statistical results can be found in Supplementary Table S5.

**Figure 5:**
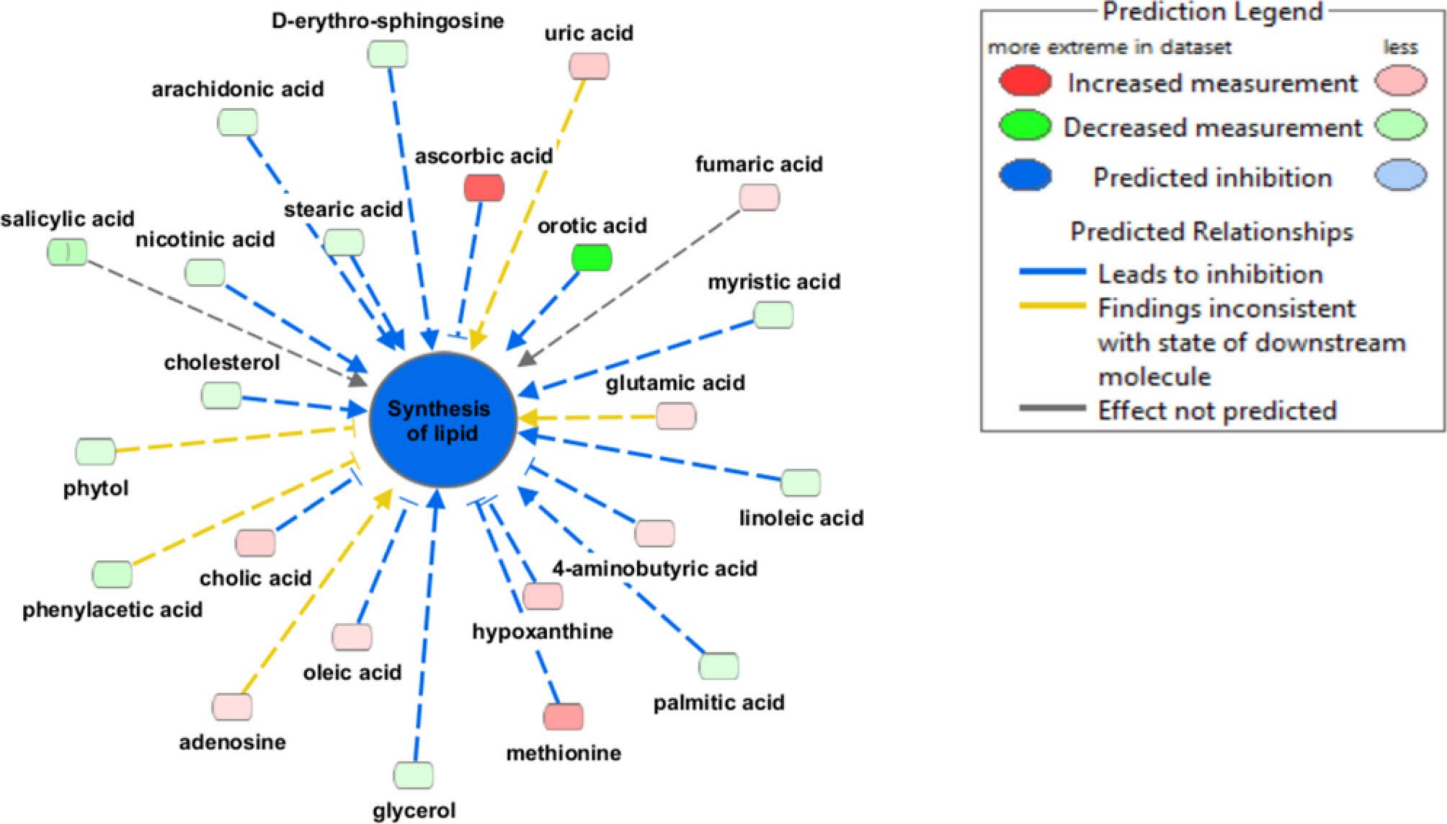
IPA of the EWAT metabolome predicts inhibition of lipid synthesis as a major mechanistic pathway differentially modulated by NNMTi treatment relative to diet switch alone. IPA included all metabolites measured in the WD/LD-T group relative to the WD/LD-V group and predicted that lipid synthesis was inhibited with NNMTi treatment (p = 0.045, activation z-score = − 2.5660).

Finally, when all metabolites were input into IPA with the log_2_ fold change for the WD/LD-T group relative to the WD/LD-V group as well as the log_2_ fold change for the WD/WD-V group relative to the LD/LD-V group, networks relevant to the phenotypes herein were identified. One of the most relevant may be synthesis of lipid, shown in Fig. 5). The changes in the abundance of metabolites linked to synthesis of lipid function resulted in the prediction that the function is inhibited in the NNMTi-treatment/LD switch combined (WD/LD-T) group relative to the LD switch alone (WD/LD-V) group, with a significant activation z-score (p = 0.0059, activation z-score = − 2.5660). In addition, IPA comparing EWAT metabolomics profiles of the WD/LD-T group to WD/LD-V group identified tRNA charging (p < 0.0001, z-score = − 3.900) and citrulline biosynthesis (p = 0.0009, z-score = − 2.000) as likely causal Canonical Pathways (significant activation z-scores of z ≥ 2.000 or ≤ − 2.000)), the latter of these may be relevant since citrullination inactivates NNMT^33^. Additional Disease and Functions and Canonical Pathways identified by IPA are reported in Supplementary Table S6.

## Methods

### Compound

The NNMT inhibitor, 5-amino-1-methylquinolium, was synthesized by a previously established synthetic method^23^. Compound was prepared weekly in a sterile environment by dissolving in 0.9% sterile saline and filtered using a sterile 0.2 µm syringe filter, then stored at 4 °C between dosing.

### Animals

Experiments were carried out in accordance with all national and local guidelines and regulations, including the Guide for the Care and Use of Laboratory Animals^34^, and with the approval of the Institutional Animal Care and Use Committee at The University of Texas Medical Branch (UTMB). All efforts were made to minimize animal suffering and to reduce the number of animals used.

Male, 18-week-old C57BL/6J diet-induced obese mice (DIO, JAX cat no. 380050) and lean control counterparts (DIO control, JAX cat no. 380056) were purchased from The Jackson Laboratory (Bar Harbor, ME, USA), and received one of two diets purchased from Research Diets, Inc. (New Brunswick, NJ, USA) during the studies: 60% high-fat diet (HFD; 60 kcal% fat, OpenSource Diets formula D12492), Western diet (WD; 45 kcal% fat, OpenSource Diets formula D12451), or lean diet (LD; 10 kcal% fat, OpenSource Diets formula D12450B). Mice were singly housed upon arrival at UTMB, provided ad libitum access to food and water in their home cages, and kept in a colony room maintained at 21–23 °C and 45–50% humidity with a 12-h light–dark cycle (lights on 6AM–6PM Central Standard Time [CST]). All studies were carried out in compliance with the ARRIVE (Animal Research: Reporting of In Vivo Experiments) guidelines (https://arriveguidelines.org).

### Study design

Twenty-two 18-week-old DIO mice transitioned from HFD to WD upon arrival to the animal care facilities, were maintained on WD for 4 weeks, then were randomized into three treatment groups balanced by average baseline body weights and composition, with two saline control groups dosed 10 µL per gram body weight (n = 6–8) and 1 experimental 5-amino-1-methylquinolinium group dosed 4 mg/mL, 10 µL per gram body weight (n = 8). Eight lean control mice were maintained on LD throughout the study and were dosed with saline at 10µL per gram of body weight (n = 8) alongside the DIO mice. The NNMTi-treated group and one of the WD saline-treated groups were switched to LD five days prior to treatment commencement. Three days after these two WD groups transitioned diets, all mice were given daily sham saline injections for two days to acclimate to injections and handling. Body weight and food intake measures were collected (obtained twice weekly), and MRI scans were performed (once each week) on the mice. Average daily food intake was an estimate obtained from food intake divided by the number of days between measurements, and an average of these twice-weekly calculated daily intake averages was used for subsequent analysis. Injection volumes were adjusted twice weekly per most recently recorded body weight for each animal. Daily dosing was performed between approximately 4PM and 6PM Central Standard Time (CST). For graphical representation of this timeline, see Fig. 1a. Mice were euthanized over the course of two days, in a non-fasted state, with five WD/LD-T animals and six animals from all other groups euthanized the first day (Day 47), and the remaining three WD/LD-T mice and two mice each in the WD/LD-V and LD/LD-V groups euthanized two days later (Day 49).

### Whole-body composition analysis

Mice were scanned weekly using an EchoMRI 4-in-1-500 Body Composition Analyzer (EchoMRI Whole Body Composition Analyzer; EchoMRI LLC, Houston, TX, USA), which was calibrated using a canola oil standard prior to scanning each day, to assess fat and lean mass. Lean mass analyzed by this method predominantly focuses on skeletal muscle mass^35^. This study did not measure total water or free water (the water stage was off during scanning), thus fat and lean mass collectively are not equal to the body weight but represent specific components of it. Two scans were taken for each animal at each time point, with 20 s between scans, and an average of these was used for further analyses. Percent fat and lean mass changes were calculated by subtracting each time point’s fat or lean mass from baseline measures, dividing by the baseline, and multiplying by 100. The ratio of whole-body fat mass/lean mass to body weight was calculated by dividing the grams of fat or lean mass by the grams of body weight at the time of scan and multiplying by 100; change was then calculated by subtracting this ratio from baseline ratio.

### Euthanizing and blood collection

Upon study completion, which was determined by collective animal weight loss, animals were injected with a lethal ketamine/xylazine combination (364 mg/kg ketamine, 36.4 mg/kg xylazine). A cardiac puncture was performed to obtain serum; blood was placed in uncoated microcentrifuge tubes. Animals were weighed and transcardially perfused with 25–30 mL phosphate-buffered saline at a rate of 10 mL/min then the respective tissues were dissected.

### Liver adiposity measure

Median liver lobes were analyzed for samples from all treatment groups. The gallbladder was not actively removed from the sample prior to imaging. Samples had been stored at 4 °C and were acclimated to room temperature, weighed, and scanned using the EchoMRI device set to ‘Tissue.’ The water stage was turned on for this scan, but not analyzed. An average of 2–3 scans was used to determine the fat and lean contents. One data point from a WD/LD-T mouse was excluded from analyses as an outlier as the lean value for that animal was zero grams in each of the three scans performed and one of the fat values was also zero, clearly indicating a machine error.

### Tissue harvests and liver histology

EWAT and liver samples were collected at study termination. Total EWAT weight was established by adding the right and left EWAT weights together. The entirety of the liver was weighed; the gallbladder was not removed for the collection of the weight. Some data points were accidentally missed during tissue harvests, including liver weight for one LD/LD-V mouse and one WD/LD-T mouse. EWATs and liver lobes were fixed in 10% neutral buffered formalin or flash frozen in liquid nitrogen, except the median liver lobes which were stored overnight at 4 °C before MRI scans. Formalin-fixed left lateral liver lobes were dehydrated with alcohol and cleared with xylene, paraffin embedded, sliced to a thickness of five microns on a SAKURA VIP6 processor, and mounted to a slide. Samples were then deparaffinized (xylene), rehydrated (reducing alcohol concentrations, then water), stained with hematoxylin and eosin, dehydrated (alcohol), cleared (xylene), mounted, and coverslipped. Images were taken on an AxioImager M2 (Zeiss) at 20X magnification using Stereo Investigator software (version 2019, 1.3, MBF Bioscience). Livers were scored for microvesicular steatosis, macrovesicular steatosis, inflammation, and hepatocellular ballooning, and also evaluated for fibrosis, by a blinded pathologist who assessed a single slide from each animal using the Kleiner et al., 2005 method^31^ with independent scoring of macrovesicular and microvesicular steatosis. Select tissues were eliminated from analyses due to technical concerns about hepatocytes demonstrating cytoplasmic loss and therefore being difficult to distinguish from adipocytes (i.e., three samples each from the LD/LD-V and WD/WD-V groups respectively, and four each from the WD/LD-V and WD/LD-T groups). The pathologist remained truly blinded throughout all studies and yet, when given multiple slides from the same animals (not included in the analyses), identically scored these slides, validating the reliability of the scoring methodology as well as the phenotype of the liver pathology across multiple tissue sections.

### Serum processing and chemistry panel analysis

Blood samples were placed in a non-coated microcentrifuge tube and allowed to clot at room temperature before being placed at 4 °C for serum processing. Clotted blood samples were processed for serum by centrifuging at 8000 rpm for 10 min at 4 °C, then the supernatant was collected, transferred to another non-coated microcentrifuge tube, and centrifuged again at 8000 rpm for 10 min at 4 °C; the second supernatant was collected in a third non-coated microcentrifuge tube and served as the final serum sample.

Serum was analyzed by the Texas A&M Veterinary Medical Diagnostic Laboratory. Only five serum samples from the WD/LD-V group, three from the WD/WD-V group, and six each from the LD/LD-V and WD/LD-T groups were analyzed, due to difficulty acquiring sufficient blood volumes from all animals. In these analyses, α-amylase, alanine transaminase (ALT), albumin, albumin/globulin ratio, alkaline phosphatase (ALKP), aspartate aminotransferase (AST), blood urea nitrogen (BUN), calcium, chloride, cholesterol, creatinine, gamma-glutamyl transferase (GGT), globulin, glucose, glutamate dehydrogenase (GLDH), phosphorus, potassium, sodium, sodium/potassium ratio, total bilirubin, and total protein were assessed (Supplementary Table S4). The degree of hemolysis was used to establish outliers, and data points accordingly were eliminated from analyses. Specifically, AST and potassium results were not reliably measured when the level of hemolysis exceeded 2, thus were excluded from the analyses. Similarly, samples with 3 + hemolysis were disregarded from α-amylase results, and samples with 4 + hemolysis from bilirubin, ALKP, phosphorous, GLDH, and GGT results.

### Metabolomics analysis

Metabolites from each mouse’s EWAT were identified using automated liner exchange-cold injection system gas chromatography time-of-flight mass spectrometry. Analytical metabolomics was performed by the West Coast Metabolomics Center, as described in Fiehn et al., 2008^36^, using untargeted analyses focused on carbohydrates, sugar phosphates, amino acids, hydroxyl acids, free fatty acids, purines, pyrimidines, aromatics, and exposome-derived chemicals. Results were normalized to the average total ion chromatogram for identified metabolites (unknowns were not included) and metabolites with BinBase names, and not the unknowns which only have BinBase IDs, were further analyzed. Heat maps were produced in MetaboAnalyst 4.0^37^ (www.metaboanalyst.ca), using only the metabolites with a significant pairwise comparison (p or q < 0.05) between the WD and LD groups in the ANOVA/Kruskal–Wallis analyses, with a Euclidian distance measure, Ward clustering algorithm, and features standardized by autoscaling; there were no missing data points. Since it was relevant to NNMTi intervention, a ratio of nicotinamide to nicotinic acid was also computed by dividing the former by the latter. Finally, the log_2_ fold change between the NNMTi- and vehicle-treated animals switched from WD to LD for all metabolites, as well as the log_2_ fold change between the WD and LD controls, were input into Ingenuity Pathway Analysis (IPA), since the significant subset alone was too small to generate many networks and pathways. Nine metabolites were not able to map into IPA: sophorose, monomyristin, maleimide, ethylsuccinate, dehydroabietic acid, conduritol-beta-epoxide, 2,5-dihydroxypyrazine, 2-deoxypentitol, 1,3-dihydroxypyridine. Direct and indirect relationships were considered, all node and data types were allowed for in experimentally observed data from all species, tissues, and cell lines, and all mutations were allowed for.

### Statistics

Statistics were performed using GraphPad Prism versions 7 and 8 (www.graphpad.com) with two-tailed hypothesis tests. All data were tested for homogeneity of variance and normality to choose the appropriate statistical test. Homogeneity of variance was assessed with an F test (two-group datasets) and a Brown-Forsythe test (> 2 group datasets) but not repeated measures; normality was always assessed with a Shapiro–Wilk test. Repeated measures data was tested for normality (sphericity was not assumed) with a Geisser-Greenhouse correction (see Supplementary Table S1 for analysis details). If the n was too low to test for normality (n ≤ 2 or identical values resulting in ≤ 2 unique n’s, i.e., in the serum data), data were analyzed non-parametrically. Log transformation was used to resolve heteroscedastic and non-normally distributed datasets, with the exception of the microvesicular and macrovesicular steatosis scores (due to zero values) and body weight/fat/lean change data (due to negative values). If the log-transformed data was non-normal, or if it was only heteroscedastic but the matching raw data was homoscedastic, the matching raw data was analyzed non-parametrically. If the log-transformed data was heteroscedastic but resolved the non-normal distribution of the raw data, the log transformed data was analyzed with the appropriate correction or test adjusting for heteroscedasticity. And if the log-transformed data was both homoscedastic and normally distributed, it was analyzed with the appropriate parametric test.

Data with two groups that were normally distributed and/or homoscedastic were analyzed with parametric (unpaired t-test) or non-parametric (Mann–Whitney) tests, and a Welch’s correction was used to adjust for heteroscedastic data. Data with three or more groups (not repeated measures) that were normally distributed and/or homoscedastic were analyzed with parametric (one-way ANOVA) or non-parametric (Kruskal–Wallis) tests, and a Welch’s ANOVA was used for heteroscedastic data. Analyses including multiple comparisons each underwent the two-stage linear step-up procedure of Benjamini, Krieger and Yekutieli to correct for multiple comparisons. Data with repeated measures missing any data points or non-normally distributed were analyzed using a mixed-effects model ANOVA whereas complete and normally distributed data were analyzed with a two-way ANOVA with repeated measures; significant pairwise comparisons for repeated measures data can be found in Supplementary Table S1. Pearson’s R correlations were run on normally distributed and homoscedastic data, while Spearman’s rank-order tests were run on non-normally distributed and/or heteroscedastic data (see Supplementary Table S3).

For the metabolomics data (Supplementary Table S5), each metabolite was analyzed individually, then the main effect p values were subjected to a Benjamini–Hochberg false-discovery rate correction (set to 5%), and multiple comparisons of significant metabolites underwent the two-stage linear step-up procedure of Benjamini, Krieger and Yekutieli-correction. In addition to main effects across all groups, the LD and WD groups were analyzed separately and, as with the main effects of all groups, results were subjected to a Benjamini–Hochberg false-discovery rate correction (set to 5%). In some instances, results demonstrated a significant corrected p value (i.e., q value) but not a significant uncorrected p value, a phenomenon sometimes called a “paradoxical result”^38^. In the studies herein, this exclusively occurred in the repeated measures data and the metabolomics data; paradoxical comparisons are reported in Supplementary Tables S1 and S5 and noted in the figure legends.

## Discussion

Under current clinical best practices, diet modifications (e.g., hypocaloric diet) and exercise are first-line interventions to manage obesity and its associated health complications, including metabolic syndrome and liver pathologies such as NAFLD/NASH^6,7,39,40^. However, only a small fraction of obese individuals can attain target weight loss from diet and exercise, and these individuals rarely sustain the weight reductions necessary to resolve obesity-linked comorbidities such as NAFLD/NASH^41–45^. Given this reality, pharmacotherapies are considered crucial to support lifestyle modifications for weight loss and associated health benefits^6^. The studies presented herein investigated the benefits of combining NNMTi treatment with a reduced-calorie diet to improve the rate and/or magnitude of body weight and adiposity loss in DIO mice. Our results demonstrated that NNMTi treatment promoted rapid, robust, and persistent body weight and fat loss compared to LD switch alone, with NNMTi-treated mice effectively attaining body weight and adiposity levels equivalent to age-matched control lean mice. In contrast, obese mice switched to LD alone achieved only modest weight and fat loss, with the effects on these parameters occurring briefly after diet switch but not continuing through the end of the study period. Furthermore, the weight and adiposity of the animals with diet switch alone remained significantly greater than their age-matched lean mice. These results align well with other work demonstrating weight regain over time in mice transitioned from energy-rich to leaner diets^25^, and in obese humans^46,47^.

Many biological factors are speculated to play a role in the limited weight loss or the weight regain that occurs only with reduced calorie diet that may be critical to overcome to sustain weight and fat loss effects^46,48^. The more rapid, more complete, and sustained weight loss effects observed with NNMTi treatment combined with LD switch versus reduced calorie diet alone provide conclusive evidence that NNMTi mitigates weight regain relative to LD switch alone, and support NNMTi as an adjunct therapy to dietary lifestyle modification. It was evident in our study that the magnitude of body weight reductions imparted by NNMTi treatment strongly correlated with the degree of whole-body adiposity loss, which is consistent with previous studies showing a positive correlation between NNMT activity and whole-body weight at the onset of obesity in mice^22^ and reduced omental and subcutaneous NNMT expression in obese humans following bariatric surgery^14^. Moreover, these results greatly extend our own and other previous work examining NNMTi in diet-induced or genetically-induced obese/diabetic mice^12,13^ and male and female NNMT knockdown or knockout mice maintained on WD^11,49^.

NNMTi treatment combined with LD switch lowered body weight primarily by reducing adiposity in obese mice EWAT and liver tissues, to a degree greater than LD switch alone. These tissue-specific effects closely align with the high expression and enzymatic activity of NNMT in mouse adipose and liver tissues^10,22,50,51^. While DIO mice do not fully recapitulate human NAFLD/NASH pathologies (e.g., fibrosis^52^), liver pathologies indicative of early-stage diagnostic features of NAFLD^53,54^, such as microvesicular and macrovesicular steatosis, were evident in WD/WD-V obese mice as has previously been reported in HFD-fed mice^55^ . LD switch alone failed to modulate liver steatosis (particularly macrovesicular steatosis) and liver fat measures, and a similar lack of an effect of reduced calorie diet alone on liver steatosis has previously been demonstrated in Ldlr^-/-^ mice switched from HFD to LD^25^. In contrast, obese animals treated with an NNMTi combined with diet switch relative to LD switch alone showed decreased liver adiposity, weight, and relative fat content, which was reflected by reduced liver steatosis scores. Importantly, NNMTi treatment with LD switch restored the fatty liver profiles in previously obese animals to levels observed in lean controls. This observation suggests that NNMTi treatment effectively counters hepatic NNMT activity; overexpression of NNMT (concurrent with nicotinamide supplementation) has been shown to drive fatty liver disease and promote steatosis, inflammation, and fibrosis which are associated with dysregulated NAD/sirtuin pathway- and methionine cycle-linked epigenetic mechanisms^21^.

Given the robust effects of NNMTi intervention on restoring whole-body adiposity in recently-obese animals to the lean state, we examined the EWAT metabolome to identify potential pathways modulated by NNMTi intervention combined with LD switch. The EWAT metabolic signature of obese mice indicated highly upregulated amino acids (essential and branched-chain amino acids [BCAAs], cysteine), downregulated vitamin B (i.e., nicotinic acid), and increased levels of many lipid metabolism pathway intermediates (e.g., fatty acid oxidation/synthesis metabolites and a cholesterol derivative), relative to lean mice. These results, particularly the amino acid findings, extend previous studies reporting global or targeted metabolomic changes in obese rodent and human adipose tissues^24,25,56–58^. Increased levels of glucogenic and ketogenic amino acids leucine, valine, and isoleucine (BCAAs), phenylalanine, tryptophan, and tyrosine were associated with the obese phenotype. These observations are in agreement with previous studies that report a well-characterized state of hyperaminoacidemia associated with obesity-related insulin resistance in humans and rodents and link it to downregulated activity of amino acid (e.g., BCAAs) catabolic enzymes^57,59–62^.

Our results indicated that formerly-obese animals (regardless of treatment condition) switched to LD for several weeks had increased abundance of metabolites involved in lipid metabolism^63,64^ (i.e., palmitoleic acid and 1-monoolein) that were still representative of the profile of obese mice, suggesting an effect largely driven by the previous exposure to obesogenic diet. The metabolites retained from the obese phenotype may override other EWAT metabolite changes associated with diet switch alone that seemingly supported potential benefits. For example, elevated levels of metabolites involved in protein metabolism and energy metabolic (e.g., malic acid in the tricarboxylic acid cycle) pathways were observed in the EWAT of WD/LD-V mice (Table 1). Similarly, significantly lower abundance of cholestenone (a cholesterol product^65^) and higher phenylacetic acid abundance (which inhibits fatty acid synthesis via pyruvate decarboxylase inhibition^66,67^) were noted in the vehicle-treated LD switch group compared to obese control mice, the latter of which normalized to the level of aged-matched lean control mice. Taken together, our data support modest changes to the obesogenic EWAT metabolome mediated by diet switch alone, however, the overall EWAT metabolomic profile of obese mice was not restored to the profile observed in lean controls by simply reducing calorie intake.

Intriguingly, the EWAT metabolome profile in mice treated with NNMTi treatment and LD switch also did not reflect the profile observed in lean controls, even though critical physiological parameters (e.g., body weight, EWAT and liver masses, relative whole body and hepatic fat masses) were restored to lean control levels. In vitro effects of NNMTi have been associated with decreases in lipogenesis and increases in the levels of NAD and S-adenosyl methionine (SAM) in differentiated 3T3-L1 adipocytes^12^. Kraus and colleagues observed upregulation of NAD and SAM in WAT of DIO mice with NNMT knockdown in vivo^11^, both co-factors that were mechanistically demonstrated to link to cellular energy metabolic regulation^17,68,69^. The current EWAT metabolomic data extend our previous study results, indicating NNMTi-associated changes linking to the NAD salvage pathway (e.g., increased nicotinamide-to-nicotinic acid ratio) and increases in metabolites in the methionine cycle and transsulfuration pathways (i.e., methionine and a trend for cysteine) that collectively suggest increased flux through these pathways critical for maintaining cellular energy homeostasis^16^ (Fig. 4).

Several other metabolites were synchronously and significantly changed in a stand-alone or additive manner in mice receiving the NNMTi/LD switch combination intervention compared to LD switch alone, including tyrosine, uridine, xylulose, and glycolic acid, the latter of which bears emerging evidence of lipase inhibitory activity^70^. Furthermore, inputting the abundance of all metabolites in NNMTi-treated mice combined with LD switch relative to LD switch alone into IPA analysis predicted inhibition of synthesis of lipid/fatty acid, cell death of liver cells/hepatocytes, liver lesion, and glucose metabolism disorder function (Fig. 5; Supplementary Table S6).

Strikingly, NNMTi treatment combined with LD switch also produced in increased levels of four out of the nine essential amino acids (specifically, leucine, lysine, methionine, tryptophan) and the non-essential amino acid tyrosine relative to LD switch alone, and the LD and WD control groups. Mechanisms linking to these observations are yet to be investigated; however, since NNMTi selectively modulates the flux through the methionine cycle that links to the folate cycle involved with the tetrahydrobiopterin salvage pathway^71^, it is possible that downstream decreases in the levels of tetrahydrobiopterin, a common co-factor for several aromatic amino acid hydroxylases^72^, may drive amino acid upregulation.

A limitation of the current study was the non-inclusion of concurrent global multi-omics assessments in the EWAT tissues. Hence, it remains to be further established if NNMTi intervention alters expression of enzymes involved in amino acid and fatty acid catabolism. Since mice were maintained on LD for prolonged periods alongside treatment, we speculate that NNMTi treatment with LD switch may shift the metabolic signature of the adipose tissue to a net “energy expenditure” state by synergistically increasing the flux of ketogenic amino acids through the TCA cycle and activating fatty acid oxidation, similar to the proposed mechanisms in other oxidative and energetically-active tissues (e.g., muscle)^73^.

The increased levels of amino acids in the EWAT metabolome analyses in the WD/LD-T group compared to LD switch alone may confer additional benefits, including impact on other tissues such as the liver and muscle. IPA analysis of the metabolome predicted significantly increased “efflux of neutral amino acid” function in the EWAT of NNMTi/LD switch mice, relative to LD switch alone, suggesting enhanced systemic transport of neutral amino acids such as leucine. Leucine has been independently shown to modulate several pathologies associated with the obesogenic state^74^ and stimulate muscle protein synthesis through established mechanisms^75^, in additional to its known effects on increasing adipocyte fatty acid oxidation (likely by Sirt1 activation)^76^ and adipose tissue and skeletal muscle resting energy expenditure^74^. Furthermore, mice fed HFD to induce hepatic steatosis and then supplemented long-term with ketogenic and/or glucogenic amino acids (*i.e*., leucine, isoleucine, valine, lysine, and threonine) exhibit reduced hepatic steatosis and fatty acid sources derived from de novo lipogenesis in the liver^77,78^, suggesting a reversal of hepatic steatosis mediated by dietary ketogenic and BCAA supplementation^78^.

In conclusion, the current study provides strong evidence for small molecule NNMTis as promising adjunct therapies to lifestyle dietary interventions to treat diet-induced obesity and related metabolic comorbidities. Particularly, the NNMTi 5-amino-1-methylquinolinium combined with LD switch produced rapid, substantial, and sustained body weight and fat loss as well as resolution of comorbid liver pathologies in obese animals beyond that of LD switch alone and restored obese animals to the lean state. Consistent with our previous studies^12,20^, treatment was well tolerated with no concerning changes to serum markers of major organ functions and electrolytic balance. The metabolomic findings in the present study point to unique cellular pathways, particularly emphasizing lipid and amino acid metabolism, that were uniquely regulated by NNMTi/LD switch combination and support the substantial body weight and fat loss in this cohort compared to LD switch alone. Collectively, NNMT’s master regulatory involvement in key metabolic processes in the metabolically active tissues (adipose, liver, and muscle) suggests that NNMT may represent an attractive, druggable target serving as a niche access point to treat obesity and related chronic metabolic diseases.

## Supplementary Information

Supplementary Information
