# Supplementary material for: Combined nicotinamide N-methyltransferase inhibition and reduced-calorie diet normalizes body composition and enhances metabolic benefits in obese mice
Source: Sci Rep. 2021 Mar 11;11:5637. doi: 10.1038/s41598-021-85051-6 (PMC7952898; doi:10.1038/s41598-021-85051-6)
Supplement: Supplementary file 1 — Supplementary Information [file 41598_2021_85051_MOESM1_ESM.pdf]

Supplementary Information

Combined Nicotinamide N-Methyltransferase Inhibition and Reduced-Calorie Diet Normalizes Body Composition and Enhances Metabolic Benefits in Obese Mice

Catherine M. Sampson□[1,2], Andrea L. Dimet (0000-0003-2214-3670)□[3], Harshini Neelakantan□[4] (0000-0003-2913-1824), Kehinde O. Ogunseye[2,5], Heather L. Stevenson[6], Jonathan D. Hommel (0000-0001-8305-3746)[1,2], and Stanley J. Watowich*[3].

□Joint first authors

[1]Department of Pharmacology and Toxicology, University of Texas Medical Branch at Galveston, Galveston, TX, USA

[2]Center for Addiction Research, University of Texas Medical Branch, Galveston, TX, USA

[3]Department of Biochemistry and Molecular Biology, University of Texas Medical Branch at Galveston, Galveston, TX, USA

[4]Ridgeline Therapeutics, Houston, TX, USA

[5]Institute for Translational Sciences, University of Texas Medical Branch, Galveston, TX, USA

[6]Department of Pathology, University of Texas Medical Branch at Galveston, Galveston, TX, USA

*Corresponding author

Supplementary Figure 1

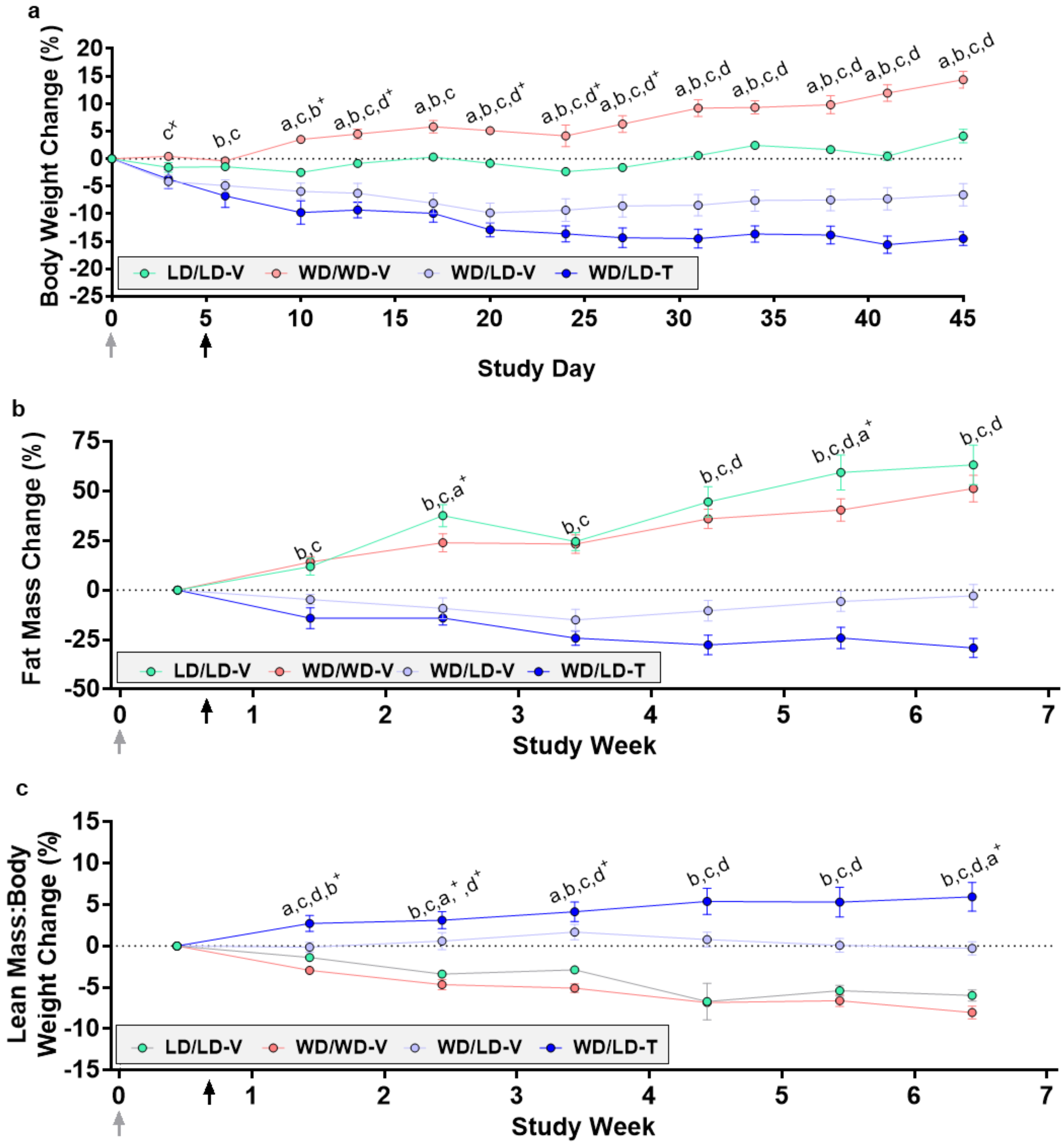

Supplementary Figure 1. NNMTi treatment improved weight and fat loss, and increased the proportion of lean mass to body weight over time, when combined with a switch from Western to lean diet. Percent body weight change from baseline (**a**), percent fat mass change from baseline (**b**); and change in the proportion of whole-body lean mass to body weight from baseline (**c**). Data are represented as mean +/- SEM; n=6-8/group; treatment start is labeled by the black arrow and diet change by the grey arrow; for **a** and **c**, some error bars are smaller than the height of the symbol. Percent body weight change from baseline demonstrated significant main effects of treatment group (p<0.0001), study day (p<0.0001), and a study day by treatment group interaction (p<0.0001). Percent fat mass change from baseline exhibited significant main effects of treatment group (p<0.0001), study week (p<0.0001), and a study week by treatment group interaction (p<0.0001). The change in the proportion of whole-body lean mass to body weight from baseline demonstrated significant main effects for treatment group (p<0.0001), study week (p=0.0100), and a study week by treatment group interaction (p<0.0001). Two-way repeated measures ANOVA (**a**-**c**). For a full list of statistical results, see **Supplementary Table 1.** LD, lean diet; -T, NNMTi-treated; -V, vehicle-treated; WD, Western diet; a, LD/LD-V vs. WD/WD-V; b, both WD/LD groups vs. LD/LD-V; c, both WD/LD groups vs. WD/WD-V; d, WD/LD-T vs. WD/LD-V. Labeled pairwise comparisons are significant (<0.05) corrected (q) and uncorrected (p) for multiple comparisons; '+' significant results with FDR correction only; 'x', significant result without FDR correction only.

Supplementary Figure 2

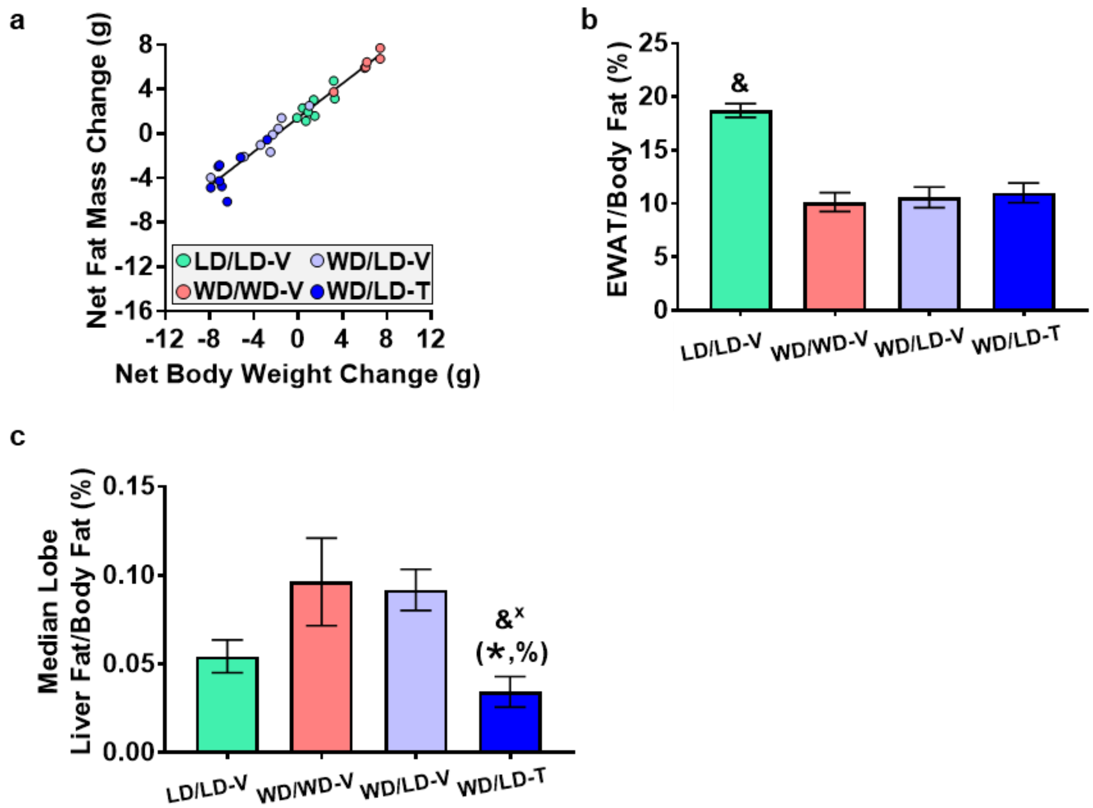

Supplementary Figure 2. Net fat mass change tightly correlated with net body weight change, NNMTi treatment combined with a lean diet switch selectively reduced liver adiposity, and long-term lean diet consumption shifts body fat distribution to the EWAT. Net fat mass change correlated to final body weight change at the end of the study (**a**), EWAT weight normalized to terminal whole-body body fat (%) (**b**), and median lobe liver fat normalized to terminal whole-body body fat (%) (**c**) Data are represented as or individual mouse values with linear regression (**a**; n=30) or mean +/- SEM (**b,c**; n=6-8/group). Net fast mass change positive correlated with final body weight change (r^2=0.9565; p<0.0001), and there were main effects of treatment group for the terminal EWAT weight (p=0.0009) and median lobe live fat (p=0.0089) each normalized to whole-body fat. Pearson's R correlation test (**a**); Kruskal-Wallis test (**b**); one-way ANOVA (**c**). For a full list of statistical results, see **Supplementary Tables 2** and **3**. LD, lean diet; T, NNMTi-treated; -V, vehicle-treated; WD, western diet. *, vs. WD-V/LD-V; %, vs. WD/WD-V; &, vs. all other groups displayed. Labeled pairwise comparisons are significant (<0.05) corrected (q) and uncorrected (p) for multiple comparisons; 'x' significant result without FDR correction only.

Supplementary Table 1

Supplementary Table 1 Significant Results from Repeated Measures Analyses. Test descriptors, main effects (F statistic, degrees of freedom numerator, degrees of freedom denominator), and significant multiple comparisons for each analysis performed on repeated measures data, as well as additional details on subject matching, including chi (χ) square (as relevant), and the Geisser-Greenhouse epsilon (ε) to estimate and correct for lack of sphericity. Tests are ordered as they are reported in the article text. 'Mid-week' defined as in the middle of the week listed; *, significant main effect of treatment group (p<0.05); g, grams; LD, lean diet; q, the false discovery rate-corrected p-value; -T, treated with the nicotinamide N-methyltransferase inhibitor; -V, vehicle-treated; WD, Western diet

**Analysis**	**Greenhouse Epsilon; Chi-Square Results of Mixed Model Analyses**	**Main Effects**	**Significant Pairwise Comparisons (with or without correction for multiple comparisons)**
Body Weight*	Mixed-effects Model; ε=0.282; χ2(1)=755.178, p<0.0001	Treatment Group: F(3,26)=18.101, p<0.0001; Study Day: F(3.668, 95.358)=12.361, p<0.0001; Study Day x Treatment Group: F(39,338)=21.440, p<0.0001	Day 0 LD/LD-V vs. WD/WD-V q=0.0003, p=0.0003; Day 0 LD/LD-V vs. WD/LD-V q<0.0001, p<0.0001; Day 0 LD/LD-V vs. WD/LD-T q<0.0001, p<0.0001; Day 3 LD/LD-V vs. WD/WD-V q=0.0002, p=0.0002; Day 3 LD/LD-V vs. WD/LD-V q=0.0002, p<0.0001; Day 3 LD/LD-V vs. WD/LD-T q<0.0001, p<0.0001; Day 6 LD/LD-V vs. WD/WD-V q=0.0002, p=0.0001; Day 6 LD/LD-V vs. WD/LD-V q=0.0002, p=0.0001; Day 6 LD/LD-V vs. WD/LD-T q=0.0004, p=0.0003; Day 10 LD/LD-V vs. WD/WD-V q<0.0001, p<0.0001; Day 10 LD/LD-V vs. WD/LD-V q=0.0003, p=0.0002; Day 10 LD/LD-V vs. WD/LD-T q=0.0006, p=0.0008; Day 10 WD/WD-V vs. WD/LD-T q=0.0086, p=0.0164; Day 13 LD/LD-V vs. WD/WD-V q<0.0001, p<0.0001; Day 13 LD/LD-V vs. WD/LD-V q=0.0005, p=0.0008; Day 13 LD/LD-V vs. WD/LD-T q=0.0005, p=0.0006; Day 13 WD/WD-V vs. WD/LD-T q=0.0050, p=0.0095; Day 17 LD/LD-V vs. WD/WD-V q=0.0001, p<0.0001; Day 17 LD/LD-V vs. WD/LD-V q=0.0020, p=0.0029; Day 17 LD/LD-V vs. WD/LD-T q=0.0012, p=0.0012; Day 17 WD/WD-V vs. WD/LD-V q=0.0197, p=0.0468; Day 17 WD/WD-V vs. WD/LD-T q=0.0032, p=0.0060; Day 20 LD/LD-V vs. WD/WD-V q<0.0001, p<0.0001; Day 20 LD/LD-V vs. WD/LD-V q=0.0009, p=0.0034; Day 20 LD/LD-V vs. WD/LD-T q=0.0009, p=0.0023; Day 20 WD/WD-V vs. WD/LD-V q=0.0058, p=0.0277; Day 20 WD/WD-V vs. WD/LD-T q=0.0009, p=0.0025; Day 20 WD/LD-V vs. WD/LD-T q=0.0489, p=0.2794; Day 24 LD/LD-V vs. WD/WD-V q<0.0001, p<0.0001; Day 24 LD/LD-V vs. WD/LD-V q=0.0005, p=0.0016; Day 24 LD/LD-V vs. WD/LD-T q=0.0005, p=0.0020; Day 24 WD/WD-V vs. WD/LD-V q=0.0062, p=0.0297; Day 24 WD/WD-V vs. WD/LD-T q=0.0001, p=0.0002; Day 24 WD/LD-V vs. WD/LD-T q=0.0337, p=0.1925; Day 27 LD/LD-V vs. WD/WD-V q<0.0001, p<0.0001; Day 27 LD/LD-V vs. WD/LD-V q=0.0005, p=0.0015; Day 27 LD/LD-V vs. WD/LD-T q=0.0035, p=0.0135; Day 27 WD/WD-V vs. WD/LD-V q=0.0037, p=0.0175; Day 27 WD/WD-V vs. WD/LD-T q=0.0001, p=0.0002; Day 27 WD/LD-V vs. WD/LD-T q=0.0173, p=0.0987; Day 31 LD/LD-V vs. WD/WD-V q<0.0001, p<0.0001; Day 31 LD/LD-V vs. WD/LD-V q=0.0006, p=0.0018; Day 31 LD/LD-V vs. WD/LD-T q=0.0045, p=0.0214; Day 31 WD/WD-V vs. WD/LD-V q=0.0010, p=0.0037; Day 31 WD/WD-V vs. WD/LD-T q<0.0001, p<0.0001; Day 31 WD/LD-V vs. WD/LD-T q=0.0108, p=0.0617; Day 34 LD/LD-V vs. WD/WD-V q<0.0001, p<0.0001; Day 34 LD/LD-V vs. WD/LD-V q=0.0006, p=0.0017; Day 34 LD/LD-V vs. WD/LD-T q=0.0059, p=0.0283; Day 34 WD/WD-V vs. WD/LD-V q=0.0017, p=0.0063; Day 34 WD/WD-V vs. WD/LD-T q=0.0001, p=0.0002; Day 34 WD/LD-V vs. WD/LD-T q=0.0100, p=0.0574; Day 38 LD/LD-V vs. WD/WD-V q<0.0001, p<0.0001; Day 38 LD/LD-V vs. WD/LD-V q=0.0005, p=0.0015; Day 38 LD/LD-V vs. WD/LD-T q=0.0068, p=0.0322; Day 38 WD/WD-V vs. WD/LD-V q=0.0011, p=0.0041; Day 38 WD/WD-V vs. WD/LD-T q<0.0001, p=0.0001; Day 38 WD/LD-V vs. WD/LD-T q=0.0085, p=0.0484; Day 41 LD/LD-V vs. WD/WD-V q<0.0001, p<0.0001; Day 41 LD/LD-V vs. WD/LD-V q=0.0003, p=0.0009; Day 41 LD/LD-V vs. WD/LD-T q=0.0102, p=0.0584; Day 41 WD/WD-V vs. WD/LD-V q=0.0014, p=0.0055; Day 41 WD/WD-V vs. WD/LD-T q=0.0002, p=0.0003; Day 41 WD/LD-V vs. WD/LD-T q=0.0035, p=0.0169; Day 45 LD/LD-V vs. WD/WD-V q<0.0001, p<0.0001; Day 45 LD/LD-V vs. WD/LD-V q=0.0010, p=0.0039; Day 45 LD/LD-V vs. WD/LD-T q=0.0302, p=0.1728; Day 45 WD/WD-V vs. WD/LD-V q=0.0007, p=0.0021; Day 45 WD/WD-V vs. WD/LD-T q<0.0001, p=0.0001; Day 45 WD/LD-V vs. WD/LD-T q=0.0041, p=0.0196

Analysis	Greenhouse Epsilon; Chi-Square Results of Mixed Model Analyses	Main Effects	Significant Pairwise Comparisons (with or without correction for multiple comparisons)
Percent Body Weight Change from Baseline*	Mixed-effects Model; ε=0.271; $\chi^2(1)$=347.354, p<0.0001	Treatment Group: F(3, 26) =37.550, p<0.0001; Study Day: F(3.253, 84.570) =10.016, p<0.0001; Study Day x Treatment Group: F(36, 312) =17.408, p<0.0001	Day 3 WD/WD-V vs. WD/LD-V q=0.0286, p=0.0055; Day 3 WD/WD-V vs. WD/LD-T q=0.0996, p=0.0490; Day 6 LD/LD-V vs. WD/LD-V q=0.0200, p=0.0172; Day 6 LD/LD-V vs. WD/LD-T q=0.0319, p=0.0405; Day 6 WD/WD-V vs. WD/LD-V q=0.0107, p=0.0034; Day 6 WD/WD-V vs. WD/LD-T q=0.0200, p=0.0191; Day 10 LD/LD-V vs. WD/WD-V q=0.0001, p<0.0001; Day 10 LD/LD-V vs. WD/LD-V q=0.0263, p=0.0627; Day 10 LD/LD-V vs. WD/LD-T q=0.0058, p=0.0110; Day 10 WD/WD-V vs. WD/LD-V q=0.0002, p=0.0002; Day 10 WD/WD-V vs. WD/LD-T q=0.0002, p=0.0003; Day 13 LD/LD-V vs. WD/WD-V q=0.0002, p=0.0007; Day 13 LD/LD-V vs. WD/LD-V q=0.0047, p=0.0223; Day 13 LD/LD-V vs. WD/LD-T q=0.0001, p=0.0003; Day 13 WD/WD-V vs. WD/LD-V q=0.0001, p=0.0004; Day 13 WD/WD-V vs. WD/LD-T q<0.0001, p<0.0001; Day 13 WD/LD-V vs. WD/LD-T q=0.0365, p=0.2087; Day 17 LD/LD-V vs. WD/WD-V q=0.0007, p=0.0033; Day 17 LD/LD-V vs. WD/LD-V q=0.0007, p=0.0027; Day 17 LD/LD-V vs. WD/LD-T q<0.0001, p=0.0003; Day 17 WD/WD-V vs. WD/LD-V q<0.0001, p<0.0001; Day 17 WD/WD-V vs. WD/LD-T q<0.0001, p<0.0001; Day 20 LD/LD-V vs. WD/WD-V q<0.0001, p<0.0001; Day 20 LD/LD-V vs. WD/LD-V q=0.0002, p=0.0011; Day 20 LD/LD-V vs. WD/LD-T q<0.0001, p<0.0001; Day 20 WD/WD-V vs. WD/LD-V q<0.0001, p<0.0001; Day 20 WD/WD-V vs. WD/LD-T q<0.0001, p<0.0001; Day 20 WD/LD-V vs. WD/LD-T q=0.0326, p=0.1860; Day 24 LD/LD-V vs. WD/WD-V q=0.0043, p=0.0205; Day 24 LD/LD-V vs. WD/LD-V q=0.0029, p=0.0110; Day 24 LD/LD-V vs. WD/LD-T q<0.0001, p<0.0001; Day 24 WD/WD-V vs. WD/LD-V q=0.0002, p=0.0005; Day 24 WD/WD-V vs. WD/LD-T q<0.0001, p<0.0001; Day 24 WD/LD-V vs. WD/LD-T q=0.0197, p=0.1124; Day 27 LD/LD-V vs. WD/WD-V q=0.0005, p=0.0019; Day 27 LD/LD-V vs. WD/LD-V q=0.0022, p=0.0104; Day 27 LD/LD-V vs. WD/LD-T q<0.0001, p<0.0001; Day 27 WD/WD-V vs. WD/LD-V q<0.0001, p<0.0001; Day 27 WD/WD-V vs. WD/LD-T q<0.0001, p<0.0001; Day 27 WD/LD-V vs. WD/LD-T q=0.0089, p=0.0507; Day 31 LD/LD-V vs. WD/WD-V q=0.0021, p=0.0013; Day 31 LD vs. WD/LD-V q=0.0026, p=0.0020; Day 31 LD/LD-V vs. WD/LD-T q<0.0001, p<0.0001; Day 31 WD/WD-V vs. WD/LD-V q<0.0001, p<0.0001; Day 31 WD/WD-V vs. WD/LD-T q<0.0001, p<0.0001; Day 31 WD/LD-V vs. WD/LD-T q=0.0376, p=0.0358; Day 34 LD/LD-V vs. WD/WD-V q=0.0017, p=0.0014; Day 34 LD/LD-V vs. WD/LD-V q=0.0017, p=0.0011; Day 34 LD/LD-V vs. WD/LD-T q<0.0001, p<0.0001; Day 34 WD/WD-V vs. WD/LD-V q<0.0001, p<0.0001; Day 34 WD/WD-V vs. WD/LD-T q<0.0001, p<0.0001; Day 34 WD/LD-V vs. WD/LD-T q=0.0282, p=0.0269; Day 38 LD/LD-V vs. WD/WD-V q=0.0038, p=0.0030; Day 38 LD/LD-V vs. WD/LD-V q=0.0032, p=0.0020; Day 38 LD/LD-V vs. WD/LD-T q<0.0001, p<0.0001; Day 38 WD/WD-V vs. WD/LD-V q<0.0001, p<0.0001; Day 38 WD/WD-V vs. WD/LD-T q<0.0001, p<0.0001; Day 38 WD/LD-V vs. WD/LD-T q=0.0280, p=0.0266; Day 41 LD/LD-V vs. WD/WD-V q=0.0003, p=0.0002; Day 41 LD/LD-V vs. WD/LD-V q=0.0067, p=0.0062; Day 41 LD/LD-V vs. WD/LD-T q<0.0001, p<0.0001; Day 41 WD/WD-V vs. WD/LD-V q<0.0001, p<0.0001; Day 41 WD/WD-V vs. WD/LD-T q<0.0001, p<0.0001; Day 41 WD/LD-V vs. WD/LD-T q=0.0067, p=0.0064; Day 45 LD/LD-V vs. WD/WD-V q=0.0005, p=0.0003; Day 45 LD/LD-V vs. WD/LD-V q=0.0011, p=0.0009; Day 45 LD/LD-V vs. WD/LD-T q<0.0001, p<0.0001; Day 45 WD/WD-V vs. WD/LD-V q<0.0001, p<0.0001; Day 45 WD/WD-V vs. WD/LD-T q<0.0001, p<0.0001; Day 45 WD/LD-Vs.. WD/LD-T q=0.0066, p=0.0063
Fat Mass*	2-Way Repeated Measures ANOVA; ε=0.368	Treatment Group: F(3,26)=24.601, p<0.0001; Study Week: F(2.209, 57.430)=11.722; Study Week x Treatment Group: F(18,156)=24.295, p<0.0001; Subject: F(26,156)=62.070, p<0.0001	mid-Week 0 LD/LD-V vs. WD/WD-V q<0.0001, p<0.0001; mid-Week 0 LD/LD-V vs. WD/LD-V q<0.0001, p<0.0001; mid-Week 0 LD/LD-V vs. WD/LD-T q<0.0001, p<0.0001; mid-Week 1 LD/LD-V vs. WD/WD-V q<0.0001, p<0.0001; mid-Week 1 LD/LD-V vs. WD/LD-V q<0.0001, p<0.0001; mid-Week 1 LD/LD-V vs. WD/LD-T q=0.0004, p=0.0005; mid-Week 1 WD/WD-V vs. WD/LD-T q=0.0096, p=0.0182; mid-Week 2 LD/LD-V vs. WD/WD-V q<0.0001, p<0.0001; mid-Week 2 LD/LD-V vs. WD/LD-V q=0.0003, p=0.0011; mid-Week 2 LD/LD-V vs. WD/LD-T q=0.0002, p=0.0004; mid-Week 2 WD/WD-V vs. WD/LD-V q=0.0072, p=0.0344; mid-Week 2 WD/WD-V vs. WD/LD-T q=0.0003, p=0.0008; mid-Week 3 LD/LD-V vs. WD/WD-V q<0.0001, p<0.0001; mid-Week 3 LD/LD-V vs. WD/LD-V q=0.0003, p=0.0009; mid-Week 3 LD/LD-V vs. WD/LD-T q=0.0004, p=0.0016; mid-Week 3 WD/WD-V vs. WD/LD-V q=0.0021, p=0.0102; mid-Week 3 WD/WD-V vs. WD/LD-T q=0.0001, p=0.0002; mid-Week 3 WD/LD-V vs. WD/LD-T q=0.0387, p=0.2212; mid-Week 4 LD/LD-V vs. WD/WD-V q<0.0001, p<0.0001; mid-Week 4 LD/LD-V vs. WD/LD-V q=0.0003, p=0.0008; mid-Week 4 LD/LD-V vs. WD/LD-T q=0.0036, p=0.0170; mid-Week 4 WD/WD-V vs. WD/LD-V q=0.0003, p=0.0013; mid-Week 4 WD/WD-V vs. WD/LD-T q<0.0001, p<0.0001; mid-Week 4 WD/LD-V vs. WD/LD-T q=0.0098, p=0.0561; mid-Week 5 LD/LD-V vs. WD/WD-V q<0.0001, p<0.0001; mid-Week 5 LD/LD-V vs. WD/LD-V q=0.0012, p=0.0006; mid-Week 5 LD/LD-V vs. WD/LD-T q=0.0308, p=0.0244; mid-Week 5 WD/WD-V vs. WD/LD-V q=0.0014, p=0.0009; mid-Week 5 WD/WD-V vs. WD/LD-T q<0.0001, p<0.0001; mid-Week 5 WD/LD-V vs. WD/LD-T q=0.0347, p=0.0330; mid-Week 6 LD/LD-V vs. WD/WD-V q<0.0001, p<0.0001; mid-Week 6 LD/LD-V vs. WD/LD-V q=0.0001, p=0.0005; mid-Week 6 LD/LD-V vs. WD/LD-T q=0.0187, p=0.1068; mid-Week 6 WD/WD-V vs. WD/LD-V q<0.0001, p=0.0002; mid-Week 6 WD/WD-V vs. WD/LD-T q<0.0001, p<0.0001; mid-Week 6 WD/LD-V vs. WD/LD-T q=0.0016, p=0.0078

Analysis	Greenhouse Epsilon; Chi-Square Results of Mixed Model Analyses	Main Effects	Significant Pairwise Comparisons (with or without correction for multiple comparisons)
Percent Fat Mass Change from Baseline*	2-Way Repeated Measures ANOVA; ε=0.419	Treatment Group: F(3,26)=38.075, p<0.0001; Study Week: F(2.094, 54.446)=26.428, p<0.0001; Study Week x Treatment Group: F(15,130)=15.614, p<0.0001; Subject: F(26,130) = 16.516	mid-Week 1 LD/LD-V vs. WD/LD-V q=0.0034, p=0.0064; mid-Week 1 LD/LD-V vs. WD/LD-T q=0.0013, p=0.0018; mid-Week 1 WD/WD-V vs. WD/LD-V q=0.0007, p=0.0004; mid-Week 1 WD/WD-V vs. WD/LD-T q=0.0007, p=0.0006; mid-Week 2 LD/LD-V vs. WD/WD-V q=0.0342, p=0.0814; mid-Week 2 LD/LD-V vs. WD/LD-V q<0.0001, p<0.0001; mid-Week 2 LD/LD-V vs. WD/LD-T q<0.0001, p<0.0001; mid-Week 2 WD/WD-V vs. WD/LD-V q=0.0002, p=0.0004; mid-Week 2 WD/WD-V vs. WD/LD-T q<0.0001, p<0.0001; mid-Week 3 LD/LD-V vs. WD/LD-V q<0.0001, p<0.0001; mid-Week 3 LD/LD-V vs. WD/LD-T q<0.0001, p<0.0001; mid-Week 3 WD/WD-V vs. WD/LD-V q<0.0001, p=0.0002; mid-Week 3 WD/WD-V vs. WD/LD-T q<0.0001, p<0.0001; mid-Week 4 LD/LD-V vs. WD/LD-V q<0.0001, p<0.0001; mid-Week 4 LD/LD-V vs. WD/LD-T q<0.0001, p<0.0001; mid-Week 4 WD/WD-V vs. WD/LD-V q<0.0001, p<0.0001; mid-Week 4 WD/WD-V vs. WD/LD-T q<0.0001, p<0.0001; mid-Week 4 WD/LD-V vs. WD/LD-T q=0.0061, p=0.0292; mid-Week 5 LD/LD-V vs. WD/WD-V q=0.0169, p=0.0965; mid-Week 5 LD/LD-V vs. WD/LD-V q<0.0001, p<0.0001; mid-Week 5 LD/LD-V vs. WD/LD-T q<0.0001, p<0.0001; mid-Week 5 WD/WD-V vs. WD/LD-V q<0.0001, p<0.0001; mid-Week 5 WD/WD-V vs. WD/LD-T q<0.0001, p<0.0001; mid-Week 5 WD/LD-V vs. WD/LD-T q=0.0053, p=0.0254; mid-Week 6 LD/LD-V vs. WD/LD-V q<0.0001, p=0.0001; mid-Week 6 LD/LD-V vs. WD/LD-T q<0.0001, p<0.0001; mid-Week 6 WD/WD-V vs. WD/LD-V q<0.0001, p<0.0001; mid-Week 6 WD/WD-V vs. WD/LD-T q<0.0001, p<0.0001; mid-Week 6 WD/LD-V vs. WD/LD-T q=0.0007, p=0.0035
Ratio of Whole-Body Fat Mass to Body Weight Change from Baseline*	2-Way Repeated Measures ANOVA; ε=0.403	Treatment Group: F(3, 26) =30.504, p<0.0001; Study Week: F(2.014, 52.373) =13.301, p<0.0001; Study Week x Treatment Group: F(15, 130) =13.312, p<0.0001; Subject: F(26, 130) =20.495, p<0.0001	mid-Week 1 LD/LD-V vs. WD/WD-V q=0.0060, p=0.0287; mid-Week 1 LD/LD-V vs. WD/LD-V q=0.0024, p=0.0090; mid-Week 1 LD/LD-V vs. WD/LD-T q=0.0024, p=0.0092; mid-Week 1 WD/WD-V vs. WD/LD-V q=0.0005, p=0.0004; mid-Week 1 WD/WD-V vs. WD/LD-T q=0.0007, p=0.0014; mid-Week 2 LD/LD-V vs. WD/LD-V q=0.0008, p=0.0015; mid-Week 2 LD/LD-V vs. WD/LD-T q<0.0001, p<0.0001; mid-Week 2 WD/WD-V vs. WD/LD-V q=0.0003, p=0.0005; mid-Week 2 WD/WD-V vs. WD/LD-T q<0.0001, p<0.0001; mid-Week 3 LD/LD-V vs. WD/WD-V q=0.0016, p=0.0076; mid-Week 3 LD/LD-V vs. WD/LD-V q=0.0005, p=0.0021; mid-Week 3 LD/LD-V vs. WD/LD-T q<0.0001, p<0.0001; mid-Week 3 WD/WD-V vs. WD/LD-V q<0.0001, p=0.0002; mid-Week 3 WD/WD-V vs. WD/LD-T q<0.0001, p<0.0001; mid-Week 4 LD/LD-V vs. WD/WD-V q=0.0009, p=0.0044; mid-Week 4 LD/LD-V vs. WD/LD-V q=0.0003, p=0.0013; mid-Week 4 LD/LD-V vs. WD/LD-T q<0.0001, p<0.0001; mid-Week 4 WD/WD-V vs. WD/LD-V q<0.0001, p=0.0001; mid-Week 4 WD/WD-V vs. WD/LD-T q<0.0001, p<0.0001; mid-Week 4 WD/LD-V vs. WD/LD-T q=0.0136, p=0.0778; mid-Week 5 LD/LD-V vs. WD/WD-V q=0.0268, p=0.0765; mid-Week 5 LD/LD-V vs. WD/LD-V q=0.0002, p=0.0005; mid-Week 5 LD/LD-V vs. WD/LD-T q<0.0001, p<0.0001; mid-Week 5 WD/WD-V vs. WD/LD-V q<0.0001, p<0.0001; mid-Week 5 WD/WD-V vs. WD/LD-T q<0.0001, p<0.0001; mid-Week 5 WD/LD-V vs. WD/LD-T q=0.0268, p=0.0665; mid-Week 6 LD/LD-V vs. WD/WD-V q=0.0149, p=0.0142; mid-Week 6 LD/LD-V vs. WD/LD-V q=0.0028, p=0.0018; mid-Week 6 LD/LD-V vs. WD/LD-T q<0.0001, p<0.0001; mid-Week 6 WD/WD-V vs. WD/LD-V q=0.0002, p=0.0001; mid-Week 6 WD/WD-V vs. WD/LD-T q<0.0001, p<0.0001; mid-Week 6 WD/LD-V vs. WD/LD-T q=0.0149, p=0.0133
Weekly Average Daily Food Intake	Mixed-effects Model; ε=0.606; $\chi^2(1)$=38.608, p<0.0001	Treatment Group: F(3, 26) =0.737, p=0.5393; Study Week: F(3.633, 93.859) =17.599, p<0.0001; Study Week x Treatment Group: F(18, 155) =8.092, p<0.0001	Week 1 LD/LD-V vs. WD/LD-V q<0.0001, p<0.0001; Week 1 LD/LD-V vs. WD/LD-T q<0.0001, p<0.0001; Week 1 WD/WD-V vs. WD/LD-V q=0.0006, p=0.0012; Week 1 WD/WD-V vs. WD/LD-T q=0.0002, p=0.0002; Week 1 WD/LD-V vs. WD/LD-T q=0.0397, p=0.0945; Week 2 LD/LD-V vs. WD/LD-T q=0.0698, p=0.0111; Week 2 WD/WD-V vs. WD/LD-T q=0.1250, p=0.0397; Week 4 LD/LD-V vs. WD/LD-V q=0.2427, p=0.0492; Week 6 LD/LD-V vs. WD/LD-V q=0.0197, p=0.0037; Week 6 WD/WD-V vs. WD/LD-V q=0.1152, p=0.0439
Lean Mass	Mixed-effects Model; ε=0.465; χ2(1)=386.237, p<0.0001	Treatment Group: F(3,26)=1.946, p=0.1469; Study Week: F(2.787, 72.462)=21.450, p<0.0001; Study Week x Treatment Group: F(18,156)=1.822, p=0.0269	mid-Week 5 LD/LD-V vs. WD/WD-V q=0.2682, p=0.0426

Analysis	Greenhouse Epsilon; Chi-Square Results of Mixed Model Analyses	Main Effects	Significant Pairwise Comparisons (with or without correction for multiple comparisons)
Percent Lean Mass Change from Baseline	Mixed-effects Model; ε=0.672; $\chi2(1)$=169.445, p<0.0001	Treatment Group: $F(3,26)=0.649$, p=0.5908; Study Week: $F(3.362,87.407)=12.353$, p<0.0001; Study Week x Treatment Group: $F(15,130)=2.516$, p=0.0027	mid-Week 6 WD/WD-V vs. WD/LD-V q=0.0612, p=0.0097; mid-Week 6 WD/WD-V vs. WD/LD-T q=0.1049, p=0.0333
Ratio of Whole-Body Lean Mass to Body Weight Change from Baseline*	2-Way Repeated Measures ANOVA; ε=0.442	Treatment Group: $F(3, 26)$ =25.536, p<0.0001; Study Week: $F(2.212, 57.516)$ =4.763, p=0.0100; Study Week x Treatment Group: $F(15, 130)$ =6.646, p<0.0001; Subject: $F(26, 130)$ =12.319, p<0.0001;	mid-Week 1 LD/LD-V vs. WD/WD-V q=0.0050, p=0.0191; mid-Week 1 LD/LD-V vs. WD/LD-V q=0.0201, p=0.1146; mid-Week 1 LD/LD-V vs. WD/LD-T q=0.0011, p=0.0031; mid-Week 1 WD/WD-V vs. WD/LD-V q=0.0011, p=0.0021; mid-Week 1 WD/WD-V vs. WD/LD-T q=0.0004, p=0.0004; mid-Week 1 WD/LD-V vs. WD/LD-T q=0.0055, p=0.0262; mid-Week 2 LD/LD-V vs. WD/WD-V q=0.0437, p=0.1250; mid-Week 2 LD/LD-V vs. WD/LD-V q=0.0029, p=0.0055; mid-Week 2 LD/LD-V vs. WD/LD-T q=0.0002, p=0.0002; mid-Week 2 WD/WD-V vs. WD/LD-V q=0.0007, p=0.0011; mid-Week 2 WD/WD-V vs. WD/LD-T q<0.0001, p<0.0001; mid-Week 2 WD/LD-V vs. WD/LD-T q=0.0437, p=0.1045; mid-Week 3 LD/LD-V vs. WD/WD-V q=0.0016, p=0.0077; mid-Week 3 LD/LD-V vs. WD/LD-V q=0.0004, p=0.0014; mid-Week 3 LD/LD-V vs. WD/LD-T q=0.0001, p=0.0003; mid-Week 3 WD/WD-V vs. WD/LD-V q<0.0001, p<0.0001; mid-Week 3 WD/WD-V vs. WD/LD-T q<0.0001, p<0.0001; mid-Week 3 WD/LD-V vs. WD/LD-T q=0.0222, p=0.1271; mid-Week 4 LD/LD-V vs. WD/LD-V q=0.0031, p=0.0119; mid-Week 4 LD/LD-V vs. WD/LD-T q=0.0003, p=0.0007; mid-Week 4 WD/WD-V vs. WD/LD-V q<0.0001, p<0.0001; mid-Week 4 WD/WD-V vs. WD/LD-T q<0.0001, p<0.0001; mid-Week 4 WD/LD-V vs. WD/LD-T q=0.0057, p=0.0270; mid-Week 5 LD/LD-V vs. WD/WD-V q=0.0405, p=0.2317; mid-Week 5 LD/LD-V vs. WD/LD-V q<0.0001, p=0.0002; mid-Week 5 LD/LD-V vs. WD/LD-T q<0.0001, p=0.0004; mid-Week 5 WD/WD-V vs. WD/LD-V q<0.0001, p<0.0001; mid-Week 5 WD/WD-V vs. WD/LD-T q<0.0001, p=0.0002; mid-Week 5 WD/LD-V vs. WD/LD-T q=0.0054, p=0.0257; mid-Week 6 LD/LD-V vs. WD/WD-V q=0.0128, p=0.0730; mid-Week 6 LD/LD-V vs. WD/LD-V q<0.0001, p=0.0001; mid-Week 6 LD/LD-V vs. WD/LD-T q<0.0001, p=0.0001; mid-Week 6 WD/WD-V vs. WD/LD-V q<0.0001, p<0.0001; mid-Week 6 WD/WD-V vs. WD/LD-T q<0.0001, p<0.0001; mid-Week 6 WD/LD-V vs. WD/LD-T q=0.0019, p=0.0091

Supplementary Table 2 Statistics for Overall Food Intake, Gross Epididymal White Adipose Tissue/Liver Weights, Liver Adiposity, and Liver Histology Scores. Test descriptors, main effects, and significant multiple comparisons for each analysis performed on the overall food intake, gross epididymal white adipose tissue (EWAT)/liver weights, liver adiposity, and liver histology scores. For unpaired and Welch's t-tests, t(degrees of freedom)=t statistics is reported, for analysis of variance results F(degrees of freedom numerator, degrees of freedom denominator)=F statistic is reported, or Kruskal-Wallis statistic is reported. Data was analyzed log transformed as necessary to resolve heteroscedasticity/non-normal distributions. Tests are ordered as they are reported in the article text. Mean±SD with range and number of mice per group listed beneath the mean (n). *, significant differences between groups with unpaired t-test, Mann-Whitney test, one-way ANOVA, or Kruskal-Wallis test (q<0.05); -- , not applicable or statistically significant with or without correction for multiple comparisons; g, grams; D, lean diet; LT, analysis performed on log-transformed data and means/SD/range reported log transformed; q, the false discovery rate-corrected p-value; -T, treated with the nicotinamide N-methyltransferase inhibitor; -V, vehicle-treated; WD, Western diet

**Dataset**	**LD/LD-V**	**WD/WD-V**	**WD/LD-V**	**WD/LD-T**	**Test Used**	**Main Effects**	**Significant Pairwise Comparisons (with and without correction for multiple comparisons)**
Total Gross EWAT Weight (g)^{LT}*	0.066±0.088 (-0.046-0.255; n=8)	0.263±0.092 (0.146-0.398; n=6)	0.089±0.110 (-0.097-0.230; n=8)	-0.053±0.084 (-0.155-0.079; n=8)	1-way ANOVA	F(3, 26)=12.894, p<0.0001	LD/LD-V vs. WD/WD-V q=0.0004, p=0.0007; WD/LD-V vs. WD/WD-V q=0.0007, p=0.0021; WD/LD-T vs. LD/LD-V q=0.0038, p=0.0179; WD/LD-T vs. WD/WD-V q<0.0001, p<0.0001; WD/LD-T vs. WD/LD-V q=0.0015, p=0.0057
Median Liver Lobe Fat (g)^{LT}*	-1.30±0.18 ([-1.53]-[-0.95]; n=8)	-1.09±0.27 ([-1.44]-[-0.70]; n=6)	-1.06±0.16 ([-1.33]-[-0.84]; n=8)	-1.47±0.24 ([-1.85]-[-1.07]; n=7)	1-way ANOVA	F(3, 25)=5.923, p=0.0034	WD/LD-T vs. WD/LD-V q=0.0040, p=0.0010; WD/LD-T vs. WD/WD-V q=0.0068, p=0.0033; LD/LD-V vs. WD/LD-V q=0.0456, p=0.0326
Gross Whole Liver Weight (g)*	1.63±0.27 (1.3-2.1; n=7)	2.20±0.29 (1.8-2.6; n=6)	2.19±0.35 (1.8-2.75; n=8)	1.69±0.28 (1.3-2.2; n=7)	1-way ANOVA	F(3,24)=7.448, p=0.0011	WD/LD-T vs. WD/LD-V q=0.0025, p=0.0036; WD/LD-T vs. WD/WD-V q=0.0030, p=0.0057; LD/LD-V vs. WD/WD-V q=0.0024, p=0.0023; LD vs. WD/LD-V q=0.0024, p=0.0014
Gross Whole Liver Weight Normalized to Body Weight	0.048±0.004 (0.041-0.053; n=7)	0.047±0.005 (0.042-0.055; n=6)	0.055±0.009 (0.042-0.068; n=8)	0.047±0.007 (0.037-0.056; n=7)	1-way ANOVA	F(3,24)=2.608, p=0.0749	--
Median Liver Lobe Fat-to-Weight Ratio*	0.112±0.041 (0.060-0.195; n=8)	0.161±0.057 (0.097-0.258; n=6)	0.158±0.038 (0.099-0.221; n=8)	0.084±0.030 (0.050-0.125; n=7)	1-way ANOVA	F(3,25)=5.593, p=0.0045	WD/LD-T vs. WD/LD-V q=0.0060, p=0.0022; WD/LD-T vs. WD/WD-V q=0.0060, p=0.0028; LD/LD-V vs. WD/WD-V q=0.0415, p=0.0395; LD/LD-V vs. WD/LD-V q=0.0415, p=0.0370
Total Gross EWAT Weight Normalized to Body Weight*	0.035±0.005 (0.029-0.044; n=8)	0.039±0.007 (0.030-0.048; n=6)	0.032±0.009 (0.023-0.047; n=8)	0.025±0.004 (0.020-0.033; n=8)	1-way ANOVA	F(3, 26)=6.521, p=0.0020	WD/WD-V vs. WD/LD-V q=0.0462, p=0.0355; WD/LD-T vs. LD/LD-V q=0.0078, p=0.0037;WD/LD-T vs. WD/WD-V q=0.0012, p=0.0003; WD/LD-T vs. WD/LD-V q=0.0462, p=0.0440
EWAT/Body Fat (%)*	18.68±1.79 (14.63-20.33; n=8)	10.14±2.20 (7.19-13.08; n=6)	10.62±2.67 (7.67-15.37; n=8)	11.06±2.58 (6.92-15.28; n=8)	Kruskal-Wallis test	Kruskal-Wallis statistic=16.534, p=0.0009	LD/LD-V vs. WD/WD-V q=0.0014, p=0.0007; LD/LD-V vs. WD/LD-V q=0.0014, p=0.0009; LD/LD-V vs. WD/LD-T p=adj.=0.0021, p=0.0020
Liver Fat/Body Fat (%)*	0.83±0.22 (0.46-1.09; n=8)	0.51±0.28 (0.19-0.96; n=6)	0.76±0.25 (0.46-1.15; n=8)	0.44±0.20 (0.15-0.74; n=7)	1-way ANOVA	F (3, 25) = 4.802, p=0.0089	LD/LD-V vs. WD/WD-V q=0.0192, p=0.0183; LD/LD-V vs. WD/LD-T q=0.0112, p=0.0035; WD/WD-V vs. WD/LD-V q=0.0436, p=0.0554; WD/LD-V vs. WD/LD-T q=0.0192, p=0.0128
Microvesicular Steatosis Score*	0.20±0.45 (0-1; n=5)	1.67±0.58 (1-2; n=3)	0.50±0.58 (0-1; n=4)	0.25±0.50 (0-1; n=4)	Kruskal-Wallis test	Kruskal-Wallis statistic=7.591, p=0.0425	WD/WD-V vs. LD/LD-V q=0.0603, p=0.0104; WD/WD-V vs. WD/LD-T q=0.0603, p=0.0192
Macrovesicular Steatosis Score*	0.20±0.45 (0-1; n=5)	1.67±0.58 (1-2; n=3)	2.0±0.82 (1-3; n=4)	0.50±0.58 (0-1; n=4)	Kruskal-Wallis test	Kruskal-Wallis statistic=10.441, p=0.0039	WD/LD-V vs. LD/LD-V q=0.0298, p=0.0057; WD/LD-V vs. WD/LD-T q=0.0613, p=0.0350; LD/LD-V vs. WD/WD-V q=0.0613, p=0.0252
Inflammation Score	0.70±1.30 (0-3; n=5)	1.00±1.00 (0-2; n=3)	0.63±0.48 (0-1; n=4)	0.25±0.50 (0-1; n=4)	Kruskal-Wallis test	Kruskal-Wallis statistic=1.989, p=0.6063	--
Hepatocyte Ballooning Score	1.30±0.97 (0-2; n=5)	1.00±1.00 (0-2; n=3)	1.00±0.82 (0-2; n=4)	1.00±0.82 (0-2; n=4)	Kruskal-Wallis test	Kruskal-Wallis statistic=0.429, p=0.9356	--

Supplementary Table 3 Correlational Analyses. Test descriptors, correlation coefficents (r), squares of the correlation coefficents (r^2), 95% confidence intervals, number of samples (n), and significance reported for each correlational analysis performed. Data was analyzed log transformed as necessary to resolve heteroscedasticity/non-normal distributions; tests are ordered as they are reported in article text. *, significant correlation; EWAT, epididymal white adipose tissue; g, grams; LT, analysis performed on log-transformed data

Data Analyzed	Test Used	R value	R^2 (from simple linear regression)	95% Confidence Interval, n	P Value
Body Weight Change (g) x Whole-Body Fat Mass Change (g)*	Pearson's Correlation	0.978	0.9565	0.954-0.990, n=30	p<0.0001
Body Weight Change (g) x Whole-Body Lean Mass Change (g)*	Spearman's Rho Test	0.570	0.3218	0.254-0.776, n=30	p=0.0010
Terminal Body Weight (g) x Gross EWAT Weight (g)*	Spearman's Rho Test	0.617	0.4938	0.320-0.803, n=30	p=0.0003
Terminal Body Fat Mass (g) x Gross EWAT Weight (g)^{LT}*	Pearson's Correlation	0.690	0.4761	0.439-0.841, n=30	p<0.0001
Terminal Body Weight (g) x Gross Liver Weight (g)^{LT}*	Pearson's Correlation	0.733	0.5374	0.496-0.869, n=28	p<0.0001
Median Lobe Liver Fat Mass (g) x Whole-Body Fat Mass (g)^{LT}*	Pearson's Correlation	0.547	0.2996	0.226-0.761, n=29	p=0.0021
Microvesicular Score x Liver Fat (g)*	Spearman's Rho Test	0.630	0.5318	0.158 to 0.868, n=15	p=0.0140
Macrovesicular Score x Liver Fat (g)*	Spearman's Rho Test	0.720	0.5286	0.315-0.904, n=15	p=0.0034

Supplementary Table 4 Serum Chemistry. Significant main effects of treatment group with one-way ANOVA or Kruskal-Wallis test ($p < 0.05$) are labeled under "measurement" (*). Statistical results report analysis of variance results (F statistic, degrees of freedom numerator, degrees of freedom denominator) or Kruskal-Wallis statistic; data was analyzed log transformed as necessary to resolve heteroscedasticity/non-normal distributions. Mean±SD with range and number of mice per group (n) listed beneath the mean; measurements are alphabetically ordered. -- , not applicable or statistically significant with or without correction for multiple comparisons; dL, deciliter; g, grams; L, liter; LD, lean diet; LT, analysis performed on log-transformed data and means/SD/range are reported log transformed; mg, milligrams; N/A, not applicable (for samples, this means the group was not analyzed); q, the false discovery rate-corrected p-value; -T, treated with the nicotinamide N-methyltransferase inhibitor; U, units; -V, vehicle-treated; WD, Western diet

**Measurement**	**LD/LD-V**	**WD/WD-V**	**WD/LD-V**	**WD/LD-T**	**Test Used**	**Main Effect of Treatment Group Statistical Result**	**Significant Pairwise Comparisons (with/without correction for multiple comparisons)**
α-Amylase (U/L)	677.60±329.72 (466-1,263; n=5)	1349.00±1486.25 (470-3,065; n=3)	816.60±412.80 (585-1,552; n=5)	1139.50±1615.71 (445-4,437; n=6)	Kruskal-Wallis test	Kruskal-Wallis statistic=4.70, p=0.1989	--
Alanine Transaminase (ALT; U/L)	92.83±57.29 (52-205; n=6)	121.67±92.22 (17-191; n=3)	130.40±88.01 (32-272; n=5)	118.17±228.50 (14-584; n=6)	Kruskal-Wallis test	Kruskal-Wallis statistic=3.96, p=0.2662	--
Albumin (g/dL)*	3.03±0.27 (2.6-3.4; n=6)	3.03±0.15 (2.9-3.2; n=3)	3.36±0.13 (3.3-3.6; n=5)	3.07±0.20 (2.7-3.2; n=6)	Kruskal-Wallis test	Kruskal-Wallis statistic=8.94, p=0.0301	LD/LD-V vs. WD/LD-V q=0.0195, p=0.0121; WD/WD-V vs. WD/LD-V q=0.0195, p=0.0186; WD/LD-V vs. WD/LD-T q=0.0195, p=0.0168
Albumin-to-Globulin Ratio	1.53±0.10 (1.4-1.7; n=6)	1.53±0.12 (1.4-1.6; n=3)	1.64±0.05 (1.6-1.7; n=5)	1.60±0.06 (1.5-1.7; n=6)	Kruskal-Wallis test	Kruskal-Wallis statistic=4.93, p=0.1767	--
Alkaline Phosphatase (ALKP; U/L)	78.60±59.50 (51-185; n=5)	77.00±42.67 (48-126; n=3)	64.80±12.52 (55-79; n=5)	59.33±31.62 (42-123; n=6)	Kruskal-Wallis test	Kruskal-Wallis statistic=4.02, p=0.2707	--
Aspartate Aminotransferase (AST; U/L)	188.20±99.74 (104-359; n=5)	162.50±140.71 (63-262; n=2)	166.33±22.05 (142-185; n=3)	376.00±604.78 (35-1,282; n=4)	Kruskal-Wallis test	Kruskal-Wallis statistic=1.25, p=0.7779	--
Blood Urea Nitrogen (BUN; mg/dL)	22.00±3.58 (19-27; n=6)	23.33±1.53 (22-25; n=3)	22.00±2.45 (20-26; n=5)	20.00±1.90 (18-23; n=6)	1-way ANOVA	F(3, 16)=1.26, p=0.3216	--
Calcium (mg/dL)	8.60±0.61 (7.9-9.4; n=6)	8.43±0.91 (7.4-9.1; n=3)	8.54±0.90 (7.1-9.4; n=5)	8.33±1.10 (6.3-9.4; n=6)	1-way ANOVA	F(3, 16)=0.10, p=0.9590	--
Chloride (mEq/L)	111.00±2.10 (108-114; n=6)	110.33±0.58 (110-111; n=3)	109.80±1.92 (108-113; n=5)	109.50±1.05 (108-111; n=6)	Kruskal-Wallis test	Kruskal-Wallis statistic=2.98, p=0.3942	--
Cholesterol (mg/dL)	132.17±46.67 (41-174; n=6)	160.00±58.56 (103-220; n=3)	178.80±23.70 (146-202; n=5)	131.50±46.78 (40-176; n=6)	Kruskal-Wallis test	Kruskal-Wallis statistic=5.04, p=0.1687	--
Creatinine (mg/dL)	0.20±0.00 (0.2; n=6)	0.20±0.00 (0.2; n=3)	0.22±0.04 (0.2-0.3; n=5)	0.20±0.00 (0.2; n=6)	Kruskal-Wallis test	Kruskal-Wallis statistic=3.00, p=0.3916	--
Gamma-Glutamyl Transferase (GGT; U/L)	< 3 (n=5)	< 3 (n=3)	< 3 (n=5)	< 3 (n=6)	N/A	N/A	N/A
Globulins (g/dL)	1.97±0.24 (1.5-2.1; n=6)	2.00±0.20 (1.8-2.2; n=3)	2.08±0.08 (2.0-2.2; n=5)	1.93±0.14 (1.7-2.1; n=6)	Kruskal-Wallis test	Kruskal-Wallis statistic=3.06, p=0.3828	--
Glucose (mg/dL)	272.33±73.12 (138-327; n=6)	262.00±81.50 (173-333; n=3)	292.60±39.11 (248-331; n=5)	244.33±90.89 (62-310; n=6)	Kruskal-Wallis test	Kruskal-Wallis statistic=1.03, p=0.7951	--
Glutamate Dehydrogenase (GLDH; U/L)	66.20±79.38 (14-206; n=5)	78.33±61.74 (20-143; n=3)	63.40±31.71 (31-100; n=5)	93.33±177.88 (14-456; n=6)	Kruskal-Wallis test	Kruskal-Wallis statistic=2.50, p=0.5027	--
Phosphorus (mg/dL)	8.94±0.27 (8.7-9.4; n=5)	7.90±1.04 (7.2-9.1; n=3)	8.72±0.86 (7.7-9.9; n=5)	8.35±1.21 (7.4-10.5; n=6)	1-way ANOVA	F(3, 15)=0.94, p=0.4465	--
Potassium (mEq/L)	5.14±0.95 (4.1-6.4; n=5)	4.75±0.21 (4.6-4.9; n=2)	4.33±0.58 (4.0-5.0; n=3)	5.05±0.82 (4.2-5.8; n=4)	Kruskal-Wallis test	Kruskal-Wallis statistic=2.29, p=0.5623	--
Sodium (mEq/L)	150.67±2.07 (147-153; n=6)	149.00±2.00 (147-151; n=3)	149.00±1.22 (148-151; n=5)	148.67±2.50 (146-153; n=6)	1-way ANOVA	F(3, 16)=1.13, p=0.3664	--
Sodium-to-Potassium Ratio	30.26±5.43 (23.8-36.8; n=5)	31.60±1.70 (30.4-32.8; n=2)	34.90±4.60 (29.6-37.8; n=3)	30.18±4.94 (25.3-35.7; n=4)	Kruskal-Wallis test	Kruskal-Wallis statistic=2.21, p=0.5767	--
Total Bilirubin (mg/dL)	0.26±0.25 (0.1-0.7; n=5)	0.20±0.00 (0.2; n=3)	0.24±0.05 (0.2-0.3; n=5)	0.27±0.15 (0.1-0.5; n=6)	Kruskal-Wallis test	Kruskal-Wallis statistic=1.36, p=0.7604	--
Total Protein (g/dL)*	5.00±0.50 (4.1-5-5; n=6)	5.03±0.29 (4.7-5.2; n=3)	5.44±0.21 (5.3-5.8; n=5)	5.00±0.32 (4.4-5.3; n=6)	Kruskal-Wallis test	Kruskal-Wallis statistic=7.97, p=0.0467	LD/LD-V vs. WD/LD-V q=0.0835, p=0.0354; WD/WD-V vs. WD/LD-V q=0.0835, p=0.0398; WD/LD-V vs. WD/LD-T q=0.0634, p=0.0101

Supplementary Table 5 Epididymal White Adipose Tissue Metabolites. Epididymal white adipose tissue metabolites with BinBase and PubChem identifiers. For analysis of variance results F(degrees of freedom numerator, degrees of freedom denominator)=F statistic is reported, for Welch's ANOVA results W ratio (degrees of freedom numerator, degrees of freedom denominator) is reported, or Kruskal-Wallis statistic is reported. For unpaired and Welch's t-test results, t(degrees of freedom) is reported; alternatively, Mann-Whitney U value is reported. Data was analyzed log transformed as necessary to resolve heteroscedasticity/non-normal distributions; the nicotinamide-to-nicotinic acid ratio was not included in the Benjamini-Hochberg correction. Mean±SD with range and number of mice per group (n) listed beneath the mean; metabolites are alphabetically ordered. *, significant main effect of treatment group with one-way ANOVA or Kruskal-Wallis test (q< 0.05); -- , not applicable or statistically significant with or without correction for multiple comparisons; LD, lean diet; LT, ANOVA or equivalent analysis analysis performed on log-transformed data (details on log transformed for t-test data found in column with test description), and means/SD/range are reported log transformed; q, the false discovery rate-corrected p-value (Benjamini-Hochberg for main effects/t-test results, for multiple pairwise comparisons the two-stage linear step-up procedure of Benjamini, Krieger, and Yekutieli was used); -T, treated with the nicotinamide N-methyltransferase inhibitor; -V, vehicle-treated; WD, Western diet

Metabolite	BinBase Identifier	PubChem Identifier	LD/LD-V	WD/WD-V	WD/LD-V	WD/LD-T	Test Used	Main Effect of Treatment Group Statistical Result	Study 2 Significant Pairwise Comparisons (with/without correction for multiple comparisons)	WD *vs.* LD only: Test Used	WD *vs.* LD only: Result
1,3-dihydroxypyridine	133655	28115	271.00±58.03 (153-321; n=8)	272.67±40.52 (215-322; n=6)	272.30±63.93 (200-416; n=8)	190.90±77.74 (72-303; n=8)	Kruskal-Wallis test	Kruskal-Wallis statistic=7.72, q=0.1452, p=0.0521	--	Unpaired t-test	t(12)=0.06, q =0.9762, p=0.9532
1,5-anhydroglucitol	209168	64960	3,187.63±915.72 (1,733-4,140; n=8)	2,278.50±428.94 (1,834-2,864; n=6)	3,835.00±812.73 (2,491-4,845; n=8)	3,270.90±1,658.65 (1,392-6,808; n=8)	1-way ANOVA	F(3, 26)=2.36, q=0.2227, p=0.0943	--	Unpaired t-test	t(12)=2.24, q =0.3060, p=0.0450
1-hexadecanol[LT]	16713	2682	3.25±0.21 (3.04-3.68; n=8)	3.21±0.08 (3.07-3.30; n=6)	3.16±0.18 (2.81-3.41; n=8)	3.02±0.17 (2.76-3.21; n=8)	1-way ANOVA	F(3, 26)=2.56, q=0.1887, p=0.0766	--	Unpaired t-test on LT data	t(12)=0.40, q =0.8790, p=0.6980
1-kestose	14692	440080	320.63±315.09 (82-1,049; n=8)	243.67±320.86 (18-882; n=6)	380.90±512.91 (93-1,496; n=8)	1,423.40±1,838.58 (35-4,146; n=8)	Kruskal-Wallis test	Kruskal-Wallis statistic=0.79, q=0.8984, p=0.8528	--	Unpaired t-test on LT data	t(12)=1.04, q =0.6124, p=0.3170
1-monoolein[LT]*	7508	5283468	3.73±0.41 (3.24-4.61; n=8)	4.24±0.24 (3.84-4.54; n=6)	4.24±0.25 (3.86-4.55; n=8)	4.42±0.23 (4.15-4.80; n=8)	1-way ANOVA	F(3, 26)=8.16, q=0.0085, p=0.0005	LD/LD-V vs. WD/WD-V q=0.0037, p=0.0036; LD/LD-V vs. WD/LD-V q=0.0028, p=0.0018; LD/LD-V vs. WD/LD-T q=0.0002, p<0.0001	Unpaired t-test on LT data	t(12)=2.72, q =0.3400, p=0.0186
1-monopalmitin	391871	14900	1,156.63±433.48 (478-1,895; n=8)	1,377.33±488.82 (866-2,267; n=6)	1,451.10±461.73 (895-2,069; n=8)	1,785.00±931.56 (967-3,616; n=8)	1-way ANOVA	F(3, 26)=1.39, q=0.3922, p=0.2676	--	Unpaired t-test	t(12)=0.89, q =0.6300, p=0.3891
1-monostearin	648	24699	640.13±172.71 (405-867; n=8)	747.17±200.78 (558-1,126; n=6)	1,037.90±469.78 (524-1,845; n=8)	747.00±412.08 (444-1,729; n=8)	Kruskal-Wallis test	Kruskal-Wallis statistic=5.63, q=0.2655, p=0.1312	--	Unpaired t-test	t(12)=1.07, q =0.5958, p=0.3049
2,5-dihydroxypyrazine	2254	23368901	3,145.75±1,359.52 (1,453-4,901; n=8)	3972.33±1,190.24 (2,257-5,834; n=6)	3,702.50±1,589.30 (1,541-6,191; n=8)	4,847.00±1,901.10 (2,468-8,198; n=8)	1-way ANOVA	F(3, 26)=1.66, q=0.3402, p=0.2001	--	Unpaired t-test	t(12)=1.18, q =0.5242, p=0.2590
2-deoxypentitol	21714	249377	88.50±20.82 (56-117; n=8)	164.83±110.57 (63-376; n=6)	117.90±44.53 (65-185; n=8)	116.80±38.95 (54-170; n=8)	1-way ANOVA	F(3, 26)=1.96, q=0.2760, p=0.1441	--	Welch's t-test	t(5.27)=1.67, q =0.4320, p=0.1530

Metabolite	BinBase Identifier	PubChem Identifier	LD/LD-V	WD/WD-V	WD/LD-V	WD/LD-T	Test Used	Main Effect of Treatment Group Statistical Result	Study 2 Significant Pairwise Comparisons (with/without correction for multiple comparisons)	WD *vs.* LD only: Test Used	WD *vs.* LD only: Result
2-hydroxyglutaric acid	2000	43	2,111.50±865.86 (946-3,716; n=8)	2,647.33±600.20 (2,254-3,809; n=6)	2,208.30±831.41 (779-3,116; n=8)	1,437.10±344.65 (919-1,893; n=8)	Kruskal-Wallis test	Kruskal-Wallis statistic=9.57, q=0.0798, p=0.0226	--	Mann-Whitney test	U=10, q =0.3291, p=0.0813
2-ketoadipic acid	106429	71	24,054.88±17,334.81 (10,493-52,396; n=8)	10,302.00±3,537.41 (7,484-15,730; n=6)	11,714.90±5,799.03 (7,668-24,292; n=8)	19,734.50±38,542.94 (2,207-114,950; n=8)	Kruskal-Wallis test	Kruskal-Wallis statistic=10.81, q=0.0544, p=0.0128	--	Unpaired t-test on LT data	t(12)=2.52, q =0.3825, p=0.0270
2-monoolein[LT]	84531	5319879	3.99±0.25 (3.64-4.50; n=8)	4.26±0.22 (3.93-4.53; n=6)	4.26±0.17 (4.02-4.48; n=8)	4.32±0.15 (4.07-4.55; n=8)	1-way ANOVA	F(3, 26)=4.37, q=0.0544, p=0.0128	--	Unpaired t-test on LT data	t(12)=2.09, q =0.3323, p=0.0587
3,4-dihydroxycinnamic acid[LT]	16546	689043	2.70±0.38 (2.19-3.18; n=8)	2.37±0.20 (2.16-2.64; n=6)	2.53±0.15 (2.34=2.74; n=8)	2.40±0.23 (2.14-2.81; n=8)	Welch's ANOVA	1.93(3.00, 13.58), q=0.3107, p=0.0876	--	Welch's t-test	t(7.87)=2.18, q =0.3256, p=0.0616
4-aminobutyric acid	1842	119	3,023.50±5,581.14 (369-16,753; n=8)	660.67±479.07 (138-1,509; n=6)	699.40±283.86 (183-1,143; n=8)	712.90±213.24 (388-1,095; n=8)	Kruskal-Wallis test	Kruskal-Wallis statistic=2.53, q=0.5891, p=0.4704	--	Unpaired t-test on LT data	t(12)=1.62, q =0.4332, p=0.1314
4-hydroxybenzoic acid	370159	135	28,485.88±13,888.89 (5,595-48,941; n=8)	13,643.67±12,361.56 (2,558-37,373; n=6)	21,148.00±11,757.23 (7,691-45,884; n=8)	29,071.50±17,600.03 (9,150-58,140; n=8)	1-way ANOVA	F(3, 26)=1.77, q=0.3141, p=0.1774	--	Unpaired t-test	t(12)=2.07, q =0.3273, p=0.0606
5-aminovaleric acid[LT]	1698	138	2.78±0.16 (2.51-2.97; n=8)	2.87±0.15 (2.62-3.05; n=6)	2.83±0.08 (2.72-2.93; n=8)	2.89±0.21 (2.69-3.26; n=8)	1-way ANOVA	F(3, 26)=0.67, q=0.6697, p=0.5791	--	Unpaired t-test	t(12)=0.94, q =0.6085, p=0.3651
5'-deoxy-5'-methylthioadenosine	21821	439176	159.63±68.89 (67-255; n=8)	176.50±63.25 (106-276; n=6)	178.30±40.48 (126-237; n=8)	191.10±68.34 (123-288; n=8)	1-way ANOVA	F(3, 26)=0.36, q=0.8440, p=0.7844	--	Unpaired t-test	t(12)=0.47, q =0.8338, p=0.6474
5-methoxytryptamine[LT*]	284	1833	2.68±0.16 (2.43-2.89; n=8)	3.44±0.27 (2.98-3.68; n=6)	3.11±0.37 (2.57-3.61; n=8)	3.54±0.32 (3.07-3.93; n=8)	1-way ANOVA	F(3, 26)=13.68, q=0.0026, p<0.0001	LD/LD-V vs. WD/WD-V q<0.0001, p<0.0001; LD/LD-V vs. WD/LD-V q=0.0036, p=0.0065; LD/LD-V vs. WD/LD-T q<0.0001, p<0.0001; WD/WD-V vs. WD/LD-V q=0.0185, p=0.0440; WD/LD-V vs. WD/LD-T q=0.0036, p=0.0069	Welch's t-test	t(5.10)=4.28, q =0.2125, p=0.0075
aconitic acid	29	643757	310.38±781.93 (9-2,245; n=8)	95.50±86.36 (40-269; n=6)	184.50±129.58 (31-413; n=8)	303.10±274.44 (87-940; n=8)	Kruskal-Wallis test	Kruskal-Wallis statistic=11.18, q=0.0556, p=0.0108	--	Mann-Whitney test	U=12, q =0.4332, p=0.1325

Metabolite	BinBase Identifier	PubChem Identifier	LD/LD-V	WD/WD-V	WD/LD-V	WD/LD-T	Test Used	Main Effect of Treatment Group Statistical Result	Study 2 Significant Pairwise Comparisons (with/without correction for multiple comparisons)	WD *vs.* LD only: Test Used	WD *vs.* LD only: Result
adenine	1764	190	1,035.88±290.97 (655-1,502; n=8)	1,088.33±309.58 (511-1,341; n=6)	1,141.80±298.19 (706-1,508; n=8)	1,150.50±440.41 (568-1,668; n=8)	1-way ANOVA	F(3, 26)=0.19, q=0.9356, p=0.9016	--	Unpaired t-test	t(12)=0.33, q =0.9098, p=0.7508
adenosine	290	60961	1,671.50±657.95 (785-2,562; n=8)	2,756.17±2,613.06 (606-6,520; n=6)	1,371.10±717.96 (467-2,269; n=8)	1,399.40±868.72 (188-2,386; n=8)	1-way ANOVA	F(3, 26)=1.55, q=0.3605, p=0.2248	--	Welch's t-test	t(5.48)=0.99, q =0.6085, p=0.3623
adenosine-5-monophosphate	1726	6083	5,304.63±3,548.86 (698-13,165; n=8)	5,164.17±3,806.22 (103-10,036; n=6)	4,746.50±3,919.80 (0-11,949; n=8)	5,515.80±4,348.24 (866-12,039; n=8)	Kruskal-Wallis test	Kruskal-Wallis statistic=0.06, q=0.9964, p=0.9964	--	Mann-Whitney test	U=21, q =0.9034, p=0.7546
alanine	18223	5950	156,176.50±84,441.16 (37,618-302,822; n=8)	175,492.33±60,834.91 (86,505-241,528; n=6)	184,503.40±51,463.25 (125,586-274,315; n=8)	211,226.30±44,569.51 (149,848-288,218; n=8)	1-way ANOVA	F(3, 26)=1.07, q=0.5145, p=0.3783	--	Unpaired t-test	t(12)=0.47, q =0.8394, p=0.6442
alpha-aminoadipic acid[LT]	125502	92136	3.27±0.20 (2.97-3.65; n=8)	3.20±0.21 (2.83-3.41; n=6)	3.28±0.29 (2.97-3.80; n=8)	3.40±0.23 (3.11-3.77; n=8)	1-way ANOVA	F(3, 26)=0.92, q=0.5727, p=0.4447	--	Unpaired t-test on LT data	t(12)=0.69, q =0.7300, p=0.5057
aminomalonate	413	100714	1,015.38±302.53 (679-1,589; n=8)	1,503.67±780.87 (556-2,596; n=6)	1,795.30±1,067.94 (621-4,125; n=8)	2,295.30±944.42 (883-3,666; n=8)	1-way ANOVA	F(3, 26)=3.31, q=0.1142, p=0.0356	--	Welch's t-test	t(6.13)=1.45, q =0.4433, p=0.1956
aniline*	401918	6115	1,283.13±746.82 (754-2,700; n=8)	683.50±309.33 (395-1,148; n=6)	685.80±156.52 (501-896; n=8)	484.40±179.57 (188-674; n=8)	Kruskal-Wallis test	Kruskal-Wallis statistic=12.94, q=0.0386, p=0.0048	LD/LD-V vs. WD/WD-V q=0.0668, p=0.0332; LD/LD-V vs. WD/LD-V q=0.0668, p=0.0382; LD/LD-V vs. WD/LD-T q=0.0020, p=0.0004	Mann-Whitney test	U=12, q =0.4232, p=0.1419
arachidic acid	291	10467	13,760.38±3,900.62 (6,566-17,995; n=8)	12,188.50±3,464.62 (9,384-17,412; n=6)	11,932.80±2,425.43 (9,522-16,736; n=8)	8,466.00±2,742.67 (4,531-13,584; n=8)	1-way ANOVA	F(3, 26)=3.95, q=0.0751, p=0.0190	--	Unpaired t-test	t(12)=0.78, q =0.6858, p=0.4498
arachidonic acid	6529	444899	24,979.63±5,974.67 (16,704-33,958; n=8)	27,886.67±4,553.21 (23,449-36,029; n=6)	29,181.50±5,640.19 (23,238-39,159; n=8)	22,898.50±3,271.76 (18,665-26,834; n=8)	1-way ANOVA	F(3, 26)=2.49, q=0.1975, p=0.0825	--	Unpaired t-test	t(12)=0.99, q =0.6181, p=0.3409
ascorbic acid[LT]	238	54670067	1.91±0.25 (1.45-2.22; n=8)	2.19±0.43 (1.43-2.79; n=6)	2.16±0.36 (1.70-2.62; n=8)	2.42±0.47 (1.90-3.43; n=8)	1-way ANOVA	F(3, 26)=2.28, q=0.2273, p=0.1033	--	Unpaired t-test on LT data	t(12)=1.49, q =0.4343, p=0.1630
asparagine[LT]	369588	6267	3.46±0.26 (2.93-3.70; n=8)	3.53±0.28 (3.08-3.89; n=6)	3.60±0.29 (3.01-3.93; n=8)	3.85±0.31 (3.33-4.32; n=8)	1-way ANOVA	F(3, 26)=2.75, q=0.1695, p=0.0628	--	Unpaired t-test	t(12)=0.68, q =0.7300, p=0.5067
aspartic acid*	79	5960	7,391.38±4,162.76 (3,330-14,838; n=8)	30,892.17±24,287.27 (3,477-68,548; n=6)	29,406.90±16,253.93 (3,188-47,349; n=8)	59,051.50±42,748.03 (12,007-149,342; n=8)	1-way ANOVA	F(3, 26)=5.26, q=0.0418, p=0.0057	LD/LD-V vs. WD/LD-T q=0.0027, p=0.0005; WD/LD-V vs. WD/LD-T q=0.0830, p=0.0316	Welch's t-test	t(5.22)=2.34, q =0.3190, p=0.0638

Metabolite	BinBase Identifier	PubChem Identifier	LD/LD-V	WD/WD-V	WD/LD-V	WD/LD-T	Test Used	Main Effect of Treatment Group Statistical Result	Study 2 Significant Pairwise Comparisons (with/without correction for multiple comparisons)	WD *vs.* LD only: Test Used	WD *vs.* LD only: Result
azelaic acid	329430	19347555	1,386.50±506.15 (343-1,899; n=8)	1,597.33±496.12 (971-2,293; n=6)	1,732.40±467.55 (1,199-2,612; n=8)	1,275.30±367.23 (825-2,027; n=8)	1-way ANOVA	F(3, 26)=1.57, q=0.3585, p=0.2214	--	Unpaired t-test	t(12)=0.78, q =0.6858, p=0.4518
behenic acid	46315	8215	4,014.13±1,306.86 (2,020-5,557; n=8)	3,670.50±1,126.54 (2,782-5,438; n=6)	3,702.90±917.50 (2,755-5,231; n=8)	2,377.60±822.84 (1,001-3,536; n=8)	1-way ANOVA	F(3, 26)=3.75, q=0.0798, p=0.0230	--	Unpaired t-test	t(12)=0.52, q =0.8308, p=0.6158
benzoic acid*	36	243	71,280.50±59,311.67 (20,052-171,036; n=8)	28,965.67±5,349.76 (19,161-33,350; n=6)	33,954.90±14,962.07 (24,710-69,479; n=8)	20,891.90±8,390.10 (8,571-31,401; n=8)	Kruskal-Wallis test	Kruskal-Wallis statistic=12.02, q=0.0466, p=0.0073	LD/LD-V vs. WD/LD-T q=0.0028, p=0.0005	Mann-Whitney test	U=6, q =0.3400, p=0.0200
beta-alanine	148	239	1,971.00±927.07 (712-3,208; n=8)	1,727.17±636.21 (1,220-2,958; n=6)	1,931.60±1,136.53 (1,296-4,720; n=8)	1,448.80±916.74 (616-3,631; n=8)	Kruskal-Wallis test	Kruskal-Wallis statistic=5.93, q=0.2420, p=0.1153	--	Unpaired t-test on LT data	t(12)=0.30, q =0.9057, p=0.7725
beta-glycerolphosphate	22021	2526	912.38±284.69 (591-1,478; n=8)	710.50±196.87 (448-1,007; n=6)	2,390.90±4,233.51 (564-12,840; n=8)	643.30±164.55 (375-863; n=8)	Kruskal-Wallis test	Kruskal-Wallis statistic=6.24, q=0.2283, p=0.1007	--	Unpaired t-test	t(12)=1.48, q =0.4328, p=0.1635
butylamine*	1871	8007	2,887.38±2,323.44 (1,177-6,777; n=8)	1,146.83±432.85 (768-1,846; n=6)	1,298.90±644.53 (660-2,676; n=8)	617.50±339.61 (56-1,046; n=8)	Kruskal-Wallis test	Kruskal-Wallis statistic=16.40, q=0.0139, p=0.0009	LD/LD-V vs. WD/WD-V q=0.0767, p=0.0438; LD/LD-V vs. WD/LD-T q=0.0003, p<0.0001; WD/LD-V vs. WD/LD-T q=0.0653, p=0.0249	Unpaired t-test on LT data	t(12)=2.38, q =0.3324, p=0.0346
cerotinic acid	17982	10469	378.63±163.67 (103-548; n=8)	304.17±128.76 (152-476; n=6)	336.00±149.24 (98-566; n=8)	267.40±90.22 (75-372; n=8)	1-way ANOVA	F(3, 26)=0.95, q=0.5679, p=0.4309	--	Unpaired t-test	t(12)=0.92, q =0.6154, p=0.3765
cholestenone[LT]*	170271	91477	1.86±0.36 (1.18-2.33; n=8)	3.20±0.49 (2.48-3.99; n=6)	2.68±0.50 (2.10-3.69; n=8)	3.01±0.50 (2.46-3.92; n=8)	1-way ANOVA	F(3, 26)=12.05, q=0.0033, p<0.0001	LD/LD-V vs. WD/WD-V q<0.0001, p<0.0001; LD/LD-V vs. WD/LD-V q=0.0017, p=0.0017; LD/LD-V vs. WD/LD-T q<0.0001, p<0.0001; WD/WD-V vs. WD/LD-V q=0.0387, p=0.0491	Unpaired t-test on LT data	t(12)=5.90, q =0.0124, p=0.0001
cholesterol	19	5997	455,244.00±183,083.25 (210,381-737,583; n=8)	644,992.50±261,867.65 (292,608-1,020,003; n=6)	513,153.80±145,097.07 (306,389-777,220; n=8)	492,895.80±133,794.96 (269,613-683,877; n=8)	1-way ANOVA	F(3, 26)=1.36, q=0.3986, p=0.2767	--	Unpaired t-test	t(12)=1.60, q =0.4281, p=0.1352
cholic acid[LT]	110403	221493	2.52±0.45 (1.90-3.35; n=8)	2.52±0.24 (2.28-2.92; n=6)	2.28±0.42 (1.81-2.86; n=8)	2.43±0.43 (1.87-2.95; n=8)	1-way ANOVA	F(3, 26)=0.58, q=0.5631, p=0.6315	--	Unpaired t-test on LT data	t(12)<0.01, q =0.9999, p=0.9999

Metabolite	BinBase Identifier	PubChem Identifier	LD/LD-V	WD/WD-V	WD/LD-V	WD/LD-T	Test Used	Main Effect of Treatment Group Statistical Result	Study 2 Significant Pairwise Comparisons (with/without correction for multiple comparisons)	WD *vs.* LD only: Test Used	WD *vs.* LD only: Result
cis-gondoic acid	87783	5282768	921.13±469.27 (378-1,737; n=8)	520.33±230.13 (96-742; n=6)	781.60±283.98 (396-1,289; n=8)	721.90±134.90 (539-890; n=8)	1-way ANOVA	F(3, 26)=1.96, q=0.2733, p=0.1447	--	Unpaired t-test	t(12)=1.91, q =0.3371, p=0.0799
citraconic acid[LT]	128883	643798	4.17±0.41 (3.30-4.60; n=8)	4.09±0.24 (3.77-4.42; n=6)	4.30±0.40 (3.52-4.74; n=8)	4.36±0.24 (4.03-4.79; n=8)	1-way ANOVA	F(3, 26)=0.87, q=0.5924, p=0.4690	--	Unpaired t-test	t(12)=0.99, q =0.6232, p=0.3400
citric acid[LT]	288	311	4.36±0.49 (3.79-5.37; n=8)	4.37±0.51 (3.39-4.80; n=6)	4.58±0.42 (3.66-5.07; n=8)	4.67±0.32 (4.39-5.35; n=8)	1-way ANOVA	F(3, 26)=0.94, q=0.5683, p=0.4359	--	Unpaired t-test on LT data	t(12)=0.03, q =0.9853, p=0.9795
citrulline	62268	9750	499.75±149.79 (274-714; n=8)	597.83±290.69 (306-981; n=6)	666.90±226.40 (342-1,066; n=8)	645.80±355.67 (101-1,292; n=8)	1-way ANOVA	F(3, 26)=0.63, q=0.6867, p=0.6019	--	Unpaired t-test	t(12)=0.83, q =0.6681, p=0.4247
conduritol-beta-epoxide[LT]	2670	9989541	2.14±0.39 (1.69-2.72; n=8)	2.13±0.43 (1.48-2.79; n=6)	2.26±0.30 (1.75-2.56; n=8)	2.16±0.51 (1.38-2.65; n=8)	1-way ANOVA	F(3, 26)=0.15, q=0.9478, p=0.9301	--	Unpaired t-test on LT data	t(12)=0.09, q =0.9779, p=0.9319
creatinine	31	588	196,316.38±427,977.75 (17,566-1,254,048; n=8)	61,151.17±32,987.49 (26,807-109,318; n=6)	71,895.10±33,433.34 (44,622-141,974; n=8)	95,877.60±65,630.76 (33,459-214,846; n=8)	Kruskal-Wallis test	Kruskal-Wallis statistic=2.32, q=0.6148, p=0.5096	--	Mann-Whitney test	U=19, q =0.8014, p=0.5728
cysteine[LT]*	65	5862	2.34±0.59 (1.45-3.31; n=8)	3.07±0.59 (2.32-4.07; n=6)	2.79±0.37 (2.18-3.29; n=8)	3.27±0.43 (2.51-3.81; n=8)	1-way ANOVA	F(3, 26)=5.22, q=0.0418, p=0.0059	LD/LD-V vs. WD/WD-V q=0.0231, p=0.0110; LD/LD-V vs. WD/LD-T q=0.0035, p=0.0008	Unpaired t-test on LT data	t(12)=2.31, q =0.3122, p=0.0397
cystine[LT]*	94	595	2.70±0.10 (2.52-2.87; n=8)	2.68±0.26 (2.23-2.88; n=6)	2.92±0.24 (2.54-3.22; n=8)	3.24±0.26 (2.84-3.60; n=8)	1-way ANOVA	F(3, 26)=10.44, q=0.0034, p=0.0001	LD/LD-V vs. WD/LD-V q=0.0347, p=0.0551; LD/LD-V vs. WD/LD-T q=0.0001, p<0.0001; WD/WD-V vs. WD/LD-V q=0.0347, p=0.0502; WD/WD-V vs. WD/LD-T q=0.0001, p<0.0001; WD/LD-V vs. WD/LD-T q=0.0089, p=0.0085	Unpaired t-test	t(12)=0.24, q =0.9466, p=0.8130
dehydroabietic acid[LT]	251689	94391	3.56±0.36 (3.06-3.92; n=8)	3.54±0.35 (3.09-3.93; n=6)	3.70±0.12 (3.56-3.90; n=8)	3.48±0.20 (3.13-3.72; n=8)	1-way ANOVA	F(3, 26)=0.97, q=0.5631, p=0.4240	--	Unpaired t-test	t(12)=0.14, q =0.9738, p=0.8936
dehydroascorbic acid[LT]	3163	440667	3.56±0.67 (2.81-4.87; n=8)	3.57±0.57 (2.75-4.35; n=6)	3.54±0.65 (2.70-4.46; n=8)	3.78±0.58 (2.79-4.36; n=8)	1-way ANOVA	F(3, 26)=0.27, q=0.9056, p=0.8490	--	Unpaired t-test on LT data	t(12)=0.05, q =0.9819, p=0.9646
D-erythro-sphingosine	126903	5280335	540.38±188.97 (281-786; n=8)	667.33±292.40 (241-1,055; n=6)	585.30±154.61 (315-736; n=8)	562.30±131.99 (312-708; n=8)	1-way ANOVA	F(3, 26)=0.54, q=0.7247, p=0.6565	--	Unpaired t-test	t(12)=0.99, q =0.6109, p=0.3420

Metabolite	BinBase Identifier	PubChem Identifier	LD/LD-V	WD/WD-V	WD/LD-V	WD/LD-T	Test Used	Main Effect of Treatment Group Statistical Result	Study 2 Significant Pairwise Comparisons (with/without correction for multiple comparisons)	WD *vs.* LD only: Test Used	WD *vs.* LD only: Result
dihydrocholesterol	42937	66066	997.38±376.47 (465-1,420; n=8)	1,704.00±1,106.61 (584-3,410; n=6)	1,227.80±551.56 (626-2,230; n=8)	1,303.80±466.28 (605-1,872; n=8)	Welch's ANOVA	1.15(3.00, 12.92), q=0.4997, p=0.3645	--	Welch's t-test	t(5.87)=1.50, q =0.4401, p=0.1852
epsilon-caprolactam	3101	7768	5,284.13±7,094.06 (279-19,749; n=8)	468.00±81.54 (336-576; n=6)	1,336.40±1,122.17 (374-3,773; n=8)	592.40±368.81 (272-1,426; n=8)	Welch's ANOVA	3.99(3.00, 13.10), q=0.1049, p=0.0321	--	Welch's t-test on LT data	t(7.22)=2.27, q =0.3312, p=0.0565
erythritol	92	222285	1,080.38±1,031.73 (415-3,589; n=8)	961.33±1,055.59 (404-3,100; n=6)	861.00±198.64 (503-1,163; n=8)	617.80±224.75 (364-1,046; n=8)	Kruskal-Wallis test	Kruskal-Wallis statistic=5.15, q=0.2973, p=0.1609	--	Mann-Whitney test	U=13, q =0.4358, p=0.1692
erythrono-1,4-lactone[LT]*	3187	5325915	2.51±0.09 (2.41-2.66; n=8)	2.76±0.25 (2.46-3.13; n=6)	2.85±0.14 (2.64-3.02; n=8)	2.80±0.19 (2.56-3.19; n=8)	1-way ANOVA	F(3, 26)=6.34, q=0.0230, p=0.0023	LD/LD-V vs. WD/WD-V q=0.0117, p=0.0111; LD/LD-V vs. WD/LD-V q=0.0014, p=0.0005; LD/LD-V vs. WD/LD-T q=0.0032, p=0.0020	Welch's t-test	t(5.25)=2.06, q =0.3468, p=0.0918
ethanolamine[LT]	45341	700	4.68±0.12 (4.50-4.86; n=8)	4.67±0.19 (4.45-4.98; n=6)	4.72±0.15 (4.57-5.04; n=8)	4.70±0.11 (4.56-4.87; n=8)	1-way ANOVA	F(3, 26)=0.19, q=0.9356, p=0.9026	--	Unpaired t-test	t(12)=0.15, q =0.9810, p=0.8829
ethylsuccinate	100863	70610	785.88±1,104.77 (39-2,600; n=8)	150.83±98.81 (81-335; n=6)	220.40±258.64 (41-840; n=8)	581.80±1471.95 (23-4,224; n=8)	Kruskal-Wallis test	Kruskal-Wallis statistic=4.26, q=0.3593, p=0.2346	--	Welch's t-test on LT data	t(9.14)=1.25, q =0.4961, p=0.2422
fructose	21	439709	89,641.63±234,773.01 (1,516-670,396; n=8)	10,063.00±12,591.91 (1,925-33,518; n=6)	7,046.30±5,323.38 (1,777-15,516; n=8)	6,354.00±6,317.65 (1,545-20,891; n=8)	Kruskal-Wallis test	Kruskal-Wallis statistic=0.43, q=0.9453, p=0.9342	--	Mann-Whitney test	U=21, q =0.9034, p=0.7546
fructose-6-phosphate[LT]*	110281	440641	3.32±0.13 (3.13-3.50; n=8)	3.33±0.27 (2.87-3.57; n=6)	3.49±0.25 (3.11-3.79; n=8)	3.72±0.26 (3.44-4.16; n=8)	1-way ANOVA	F(3, 26)=4.85, q=0.0470, p=0.0082	LD/LD-V vs. WD/LD-T q=0.0090, p=0.0021; WD/WD-V vs. WD/LD-T q=0.0099, p=0.0047	Unpaired t-test	t(12)=0.56, q =0.8052, p=0.5826
fucose	3009	439650	386.00±599.33 (132-1,867; n=8)	294.67±308.05 (75-906; n=6)	217.10±34.77 (170-264; n=8)	184.50±68.02 (84-301; n=8)	Kruskal-Wallis test	Kruskal-Wallis statistic=2.49, q=0.5922, p=0.4775	--	Mann-Whitney test	U=23, q =0.9762, p=0.9497
fumaric acid	1718	444972	3,025.13±1,219.96 (1,412-5,121; n=8)	4,524.50±1,876.39 (2,262-7,033; n=6)	5,030.30±2,090.16 (1,424-7,288; n=8)	6,230.30±1,857.12 (3,358-8,292; n=8)	1-way ANOVA	F(3, 26)=4.42, q=0.0561, p=0.0122	--	Unpaired t-test	t(12)=1.82, q =0.3485, p=0.0943

Metabolite	BinBase Identifier	PubChem Identifier	LD/LD-V	WD/WD-V	WD/LD-V	WD/LD-T	Test Used	Main Effect of Treatment Group Statistical Result	Study 2 Significant Pairwise Comparisons (with/without correction for multiple comparisons)	WD *vs.* LD only: Test Used	WD *vs.* LD only: Result
galactinol[LT]	3164	11727586	2.11±0.51 (1.04-2.70; n=8)	2.15±0.39 (1.76-2.81; n=6)	2.29±0.33 (1.76-2.69; n=8)	2.23±0.51 (1.28-2.73; n=8)	1-way ANOVA	F(3, 26)=0.26, q=0.8984, p=0.8561	--	Unpaired t-test on LT data	t(12)=0.14, q =0.9794, p=0.8896
galactose-6-phosphate	84204	439404	50.50±20.72 (8-77; n=8)	76.17±41.86 (29-132; n=6)	60.10±23.74 (36-107; n=8)	42.80±26.13 (10-93; n=8)	1-way ANOVA	F(3, 26)=1.79, q=0.3124, p=0.1746	--	Unpaired t-test	t(12)=1.52, q =0.4320, p=0.1550
glucoheptulose	3191	5459879	618.38±324.38 (181-1,120; n=8)	320.83±245.98 (90-774; n=6)	418.60±142.78 (272-747; n=8)	627.30±416.75 (295-1,350; n=8)	Kruskal-Wallis test	Kruskal-Wallis statistic=4.33, q=0.3587, p=0.2276	--	Unpaired t-test	t(12)=1.87, q =0.3388, p=0.0857
gluconic acid	7501	6857417	206.38±63.62 (149-341; n=8)	179.17±51.95 (110-247; n=6)	220.80±100.83 (72-413; n=8)	320.30±316.78 (145-1,097; n=8)	Kruskal-Wallis test	Kruskal-Wallis statistic=1.80, q=0.6963, p=0.6147	--	Unpaired t-test	t(12)=0.85, q =0.6571, p=0.4102
glucose	22	64689	51,854.75±12,040.02 (33,733-71,088; n=8)	77,373.00±58,464.20 (29,491-176,674; n=6)	82,488.80±19,858.74 (42,921-101,575; n=8)	158,386.00±235,592.00 (21,662-733,903; n=8)	Kruskal-Wallis test	Kruskal-Wallis statistic=4.60, q=0.3424, p=0.2034	--	Welch's t-test	t(5.32)=1.05, q =0.6283, p=0.3380
glucose-1-phosphate[LT]	3167	65533	3.28±0.18 (3.02-3.46; n=8)	3.29±0.15 (3.04-3.46; n=6)	3.28±0.17 (3.07-3.61; n=8)	3.33±0.28 (2.93-3.66; n=8)	1-way ANOVA	F(3, 26)=0.09, q=0.9700, p=0.9643	--	Unpaired t-test	t(12)=0.08, q =0.9782, p=0.9401
glucose-6-phosphate[LT]	308	5958	3.47±0.14 (3.28-3.66; n=8)	3.28±0.61 (2.36-3.80; n=6)	3.65±0.24 (3.23-3.88; n=8)	3.85±0.26 (3.54-4.33; n=8)	1-way ANOVA	F(3, 26)=3.78, q=0.0800, p=0.0225	--	Welch's t-test	t(6.14)=0.18, q =0.9630, p=0.8610
glutamic acid	28	33032	56,896.75±52,400.59 (22,333-184,658; n=8)	61954.00±22777.49 (31,061-90,564; n=6)	67,183.40±22,822.20 (25,963-96,658; n=8)	72,247.00±27,929.05 (39,091-131,544; n=8)	Kruskal-Wallis test	Kruskal-Wallis statistic=5.21, q=0.2931, p=0.1569	--	Mann-Whitney test	U=14, q =0.4915, p=0.2284
glutamine	18	5961	100,227.25±29,542.01 (59,954-131,175; n=8)	138,678.50±30,735.27 (97,253-178,388; n=6)	116,207.30±13,678.91 (90,473-136,038; n=8)	134,584.00±39,552.13 (92,373-214,634; n=8)	1-way ANOVA	F(3, 26)=2.62, q=0.1827, p=0.0720	--	Unpaired t-test	t(12)=2.37, q =0.3167, p=0.0354
glyceric acid	48	439194	4,825.50±8,805.24 (873-26,583; n=8)	2,926.83±1,346.93 (1,788-4,697; n=6)	2,954.80±1,099.77 (1,402-4,880; n=8)	2,205.50±795.17 (1,144-3,660; n=8)	Kruskal-Wallis test	Kruskal-Wallis statistic=4.58, q=0.3427, p=0.2056	--	Mann-Whitney test	U=13, q =0.4464, p=0.1812
glycerol[LT]	102844	753	5.77±0.35 (5.35-6.32; n=8)	5.54±0.17 (5.27-5.79; n=6)	5.65±0.13 (5.48-5.92; n=8)	5.58±0.04 (5.53-5.64; n=8)	1-way ANOVA	F(3, 26)=1.70, q=0.3299, p=0.1921	--	Unpaired t-test on LT data	t(12)=1.49, q =0.4398, p=0.1627
glycerol-3-galactoside[LT]	100875	16048618	3.48±0.31 (3.02-3.91; n=8)	3.46±0.24 (3.16-3.77; n=6)	3.73±0.12 (3.57-3.98; n=8)	3.77±0.33 (3.23-4.12; n=8)	1-way ANOVA	F(3, 26)=2.66, q=0.1788, p=0.0694	--	Unpaired t-test	t(12)=0.38, q =0.8754, p=0.7102

Metabolite	BinBase Identifier	PubChem Identifier	LD/LD-V	WD/WD-V	WD/LD-V	WD/LD-T	Test Used	Main Effect of Treatment Group Statistical Result	Study 2 Significant Pairwise Comparisons (with/without correction for multiple comparisons)	WD *vs.* LD only: Test Used	WD *vs.* LD only: Result
glycerol-alpha-phosphate	46174	754	18,458.88±6,933.22 (11,848-33,367; n=8)	19,444.17±4,218.14 (14,133-23,745; n=6)	63,257.00±112,189.20 (12,886-340,157; n=8)	16,969.30±4,337.30 (10,611-23,251; n=8)	Kruskal-Wallis test	Kruskal-Wallis statistic=4.50, q=0.3480, p=0.2124	--	Unpaired t-test	t(12)=0.31, q =0.9027, p=0.7646
glycine	6	750	52,387.75±12,926.45 (33,228-74,152; n=8)	62,742.50±14,943.20 (42,010-83,969; n=6)	70,651.30±15,712.06 (57,798-105,245; n=8)	74,715.30±12,342.47 (49,336-87,084; n=8)	Kruskal-Wallis test	Kruskal-Wallis statistic=10.50, q=0.0599, p=0.0148	--	Unpaired t-test	t(12)=1.39, q =0.4425, p=0.1900
glycolic acid*	1971	757	7,090.88±1,711.87 (3,042-8,339; n=8)	5,927.17±1,515.89 (4,711-8,879; n=6)	5,812.90±1,676.06 (4,555-8,573; n=8)	3,674.50±1,428.89 (1,805-6,070; n=8)	Kruskal-Wallis test	Kruskal-Wallis statistic=11.79, q=0.0481, p=0.0081	LD/LD-V vs. WD/LD-T q=0.0047, p=0.0009; WD/WD-V vs. WD/LD-T q=0.0596, p=0.0227; WD/LD-V vs. WD/LD-T q=0.0668, p=0.0382	Mann-Whitney test	U=13, q =0.4401, p=0.1812
guanosine[LT]	1966	6802	2.46±0.15 (2.25-2.68; n=8)	2.62±0.11 (2.49-2.76; n=6)	2.62±0.32 (2.15-3.09; n=8)	2.68±0.21 (2.37-2.90; n=8)	1-way ANOVA	F(3, 26)=1.53, q=0.3576, p=0.2314	--	Unpaired t-test	t(12)=2.16, q =0.3274, p=0.0520
heptadecanoic acid	727	10465	20,829.25±5,580.20 (11,073-26,294; n=8)	21,999.00±5,863.28 (16,496-29,988; n=6)	20,646.00±4,939.61 (15,316-29,583; n=8)	15,452.60±5,267.15 (9,428-25,806; n=8)	1-way ANOVA	F(3, 26)=2.22, q=0.2348, p=0.1102	--	Unpaired t-test	t(12)=0.38, q =0.8754, p=0.7106
hypoxanthine*	1663	790	5,839.13±2,467.30 (1,739-9,437; n=8)	11,304.83±7,272.84 (1,607-19,315; n=6)	16,410.90±12,690.11 (3,026-44,647; n=8)	22,452.90±7,092.86 (8,912-29,540; n=8)	Kruskal-Wallis test	Kruskal-Wallis statistic=14.02, q=0.0274, p=0.0029	LD/LD-V vs. WD/LD-V q=0.0541, p=0.0309; LD/LD-V vs. WD/LD-T q=0.0013, p=0.0002; WD/WD-V vs. WD/LD-T q=0.0541, p=0.0304	Welch's t-test	t(5.87)=1.77, q =0.4380, p=0.1289
inosine*	84524	6021	7,187.50±1,469.54 (5,474-9,651; n=8)	12,198.50±5,990.20 (1,665-19,980; n=6)	23,034.10±18,366.01 (3,726-63,791; n=8)	25,718.90±13,395.88 (7,729-44,699; n=8)	Welch's ANOVA	7.24(3.00, 10.76), q=0.0422, p=0.0062	LD/LD-V vs. WD/LD-V q=0.0784, p=0.0448; LD/LD-V vs. WD/LD-T q=0.0300, p=0.0057; WD/WD-V vs. WD/LD-T q=0.0764, p=0.0291	Welch's t-test	t(5.45)=2.00, q =0.3494, p=0.0966
inositol-4-monophosphate	4759	440043	1,797.63±1,166.06 (139-3,743; n=8)	1,324.00±529.28 (612-1,853; n=6)	1,660.60±867.48 (623-3,425; n=8)	1,251.40±364.00 (722-1,812; n=8)	1-way ANOVA	F(3, 26)=0.80, q=0.6169, p=0.5044	--	Unpaired t-test	t(12)=0.92, q =0.6154, p=0.3760
inulotriose	14686	22833608	136.75±140.17 (32-467; n=8)	189.00±247.84 (37-668; n=6)	346.80±486.81 (56-1,297; n=8)	83.50±58.57 (12-184; n=8)	Kruskal-Wallis test	Kruskal-Wallis statistic=1.77, q=0.6948, p=0.6212	--	Unpaired t-test on LT data	t(12)=0.11, q =0.9820, p=0.9155
isocitric acid[LT]	32122	5318532	2.65±0.46 (2.06-3.61; n=8)	2.80±0.23 (2.39-3.09; n=6)	2.86±0.41 (2.00-3.40; n=8)	2.95±0.31 (2.61-3.61; n=8)	1-way ANOVA	F(3, 26)=0.94, q=0.5683, p=0.4379	--	Unpaired t-test on LT data	t(12)=0.71, q =0.7255, p=0.4883

Metabolite	BinBase Identifier	PubChem Identifier	LD/LD-V	WD/WD-V	WD/LD-V	WD/LD-T	Test Used	Main Effect of Treatment Group Statistical Result	Study 2 Significant Pairwise Comparisons (with/without correction for multiple comparisons)	WD *vs.* LD only: Test Used	WD *vs.* LD only: Result
isoleucine	15	6306	38,267.63±23,380.93 (22,757-94,234; n=8)	69,054.67±35,156.01 (21,838-103,569; n=6)	68,730.50±28,585.30 (35,575-115,437; n=8)	124,266.00±78,793.41 (52,033-290,017; n=8)	Kruskal-Wallis test	Kruskal-Wallis statistic=10.96, q=0.0567, p=0.0119	--	Mann-Whitney test	U=14, q =0.4854, p=0.2284
isolinoleic acid[LT]	10647	5312483	2.18±0.39 (1.41-2.56; n=8)	2.09±0.25 (1.78-2.38; n=6)	2.00±0.44 (1.36-2.87; n=8)	2.46±0.42 (1.98-3.29; n=8)	1-way ANOVA	F(3, 26)= 2.09, q=0.2610, p=0.8727	--	Unpaired t-test	t(12)=0.97, q =0.6024, p=0.3508
isothreonic acid	1679	151152	346.25±104.43 (179-471; n=8)	618.50±374.41 (314-1,327; n=6)	499.10±77.43 (376-616; n=8)	456.30±73.06 (326-543; n=8)	1-way ANOVA	F(3, 26)=2.66, q=0.1788, p=0.0690	--	Welch's t-test	t(5.59)=1.73, q =0.4259, p=0.1378
itaconic acid[LT]	101725	811	4.58±0.34 (4.01-4.97; n=8)	4.32±0.16 (4.14-4.52; n=6)	4.50±0.24 (4.26-4.88; n=8)	4.35±0.25 (3.99-4.81; n=8)	1-way ANOVA	F(3, 26)=1.96, q=0.2733, p=0.1445	--	Welch's t-test	t(8.23)=2.23, q =0.3312, p=0.0554
lactic acid[LT]	80	612	5.13±0.13 (4.95-5.35; n=8)	5.11±0.16 (4.88-5.29; n=6)	5.13±0.15 (4.86-5.32; n=8)	5.23±0.22 (4.97-5.52; n=8)	1-way ANOVA	F(3, 26)=0.76, q=0.6248, p=0.5292	--	Unpaired t-test	t(12)=0.20, q =0.9628, p=0.8439
lactose	217651	6134	2,127.13±1,088.22 (716-4,113; n=8)	2,898.17±5,445.86 (252-13,994; n=6)	1,972.50±995.43 (523-3,018; n=8)	2,594.40±3,281.65 (93-9,737; n=8)	Kruskal-Wallis test	Kruskal-Wallis statistic=2.29, q=0.6150, p=0.5137	--	Welch's t-test on LT data	t(6.14)=1.04, q =0.6314, p=0.3362
lanosterol	133475	246983	89.00±48.14 (40-193; n=8)	126.50±51.43 (65-202; n=6)	124.40±36.38 (63-165; n=8)	118.50±68.12 (36-210; n=8)	1-way ANOVA	F(3, 26)=0.85, q=0.5922, p=0.4807	--	Unpaired t-test	t(12)=1.40, q =0.4401, p=0.1864
lauric acid[LT]	49	3893	4.42±0.13 (4.20-4.56; n=8)	4.38±0.13 (4.22-4.53; n=6)	4.48±0.23 (4.26-4.89; n=8)	4.18±0.18 (3.90-4.39; n=8)	1-way ANOVA	F(3, 26)=4.64, q=0.0531, p=0.0100	--	Unpaired t-test	t(12)=0.58, q =0.8014, p=0.5751
leucine*	9	6106	76,138.63±23,928.64 (50,464-125,633; n=8)	175,005.50±83,715.30 (55,349-270,918; n=6)	177,467.90±84,555.93 (88,092-323,321; n=8)	327,353.40±188,924.60 (147,186-726,695; n=8)	1-way ANOVA	F(3, 26)=6.58, q=0.0215, p=0.0019	LD/LD-V vs. WD/LD-T q=0.0005, p=0.0002; WD/WD-V vs. WD/LD-T q=0.0214, p=0.0204; WD/LD-V vs. WD/LD-T q=0.0214, p=0.0143	Welch's t-test	t(5.62)=2.81, q =0.3676, p=0.0331
levoglucosan	3169	2724705	384.50±100.44 (203-509; n=8)	316.67±125.04 (210-510; n=6)	371.40±151.17 (155-682; n=8)	332.30±244.88 (93-869; n=8)	1-way ANOVA	F(3, 26)=0.26, q=0.9005, p=0.8523	--	Unpaired t-test	t(12)=1.28, q =0.5582, p=0.2814
linoleic acid	165	5280450	20,600.25±8,693.38 (13,326-38,685; n=8)	21,767.83±3,120.39 (15,466-23,791; n=6)	25,147.60±8,955.17 (13,702-41,355; n=8)	24,866.90±5,861.84 (18,768-35,107; n=8)	Kruskal-Wallis test	Kruskal-Wallis statistic=2.92, q=0.5449, p=0.4039	--	Mann-Whitney test	U=14, q =0.4794, p=0.2284

Metabolite	BinBase Identifier	PubChem Identifier	LD/LD-V	WD/WD-V	WD/LD-V	WD/LD-T	Test Used	Main Effect of Treatment Group Statistical Result	Study 2 Significant Pairwise Comparisons (with/without correction for multiple comparisons)	WD *vs.* LD only: Test Used	WD *vs.* LD only: Result
linolenic acid	4704	5280934	13,579.00±15,413.26 (1,658-46,453; n=8)	6,637.17±5,693.63 (3,128-18,009; n=6)	15,396.80±18,498.58 (2,787-53,197; n=8)	20,016.90±16,839.20 (3,150-43,584; n=8)	Kruskal-Wallis test	Kruskal-Wallis statistic=1.78, q=0.6948, p=0.6185	--	Mann-Whitney test	U=20, q =0.8462, p=0.6620
lysine*	12	5962	5,826.25±2,183.29 (2,777-8,414; n=8)	10,479.83±6,329.03 (2,226-17,386; n=6)	9,575.00±3,787.36 (4,655-14,867; n=8)	21,211.90±11,587.18 (6,979-39,167; n=8)	1-way ANOVA	F(3, 26)=7.10, q=0.0170, p=0.0012	LD/LD-V vs. WD/LD-T q=0.0005, p=0.0002; WD/WD-V vs. WD/LD-T q=0.0091, p=0.0087; WD/LD-V vs. WD/LD-T q=0.0042, p=0.0026	Welch's t-test	t(5.90)=1.73, q =0.4259, p=0.1360
lyxitol	233	439255	3,859.00±1,074.78 (2,509-5,853; n=8)	2,878.83±820.38 (1,702-3,908; n=6)	4,650.00±1,708.87 (3,003-8,057; n=8)	3,561.40±2,226.90 (931-8,047; n=8)	1-way ANOVA	F(3, 26)=1.48, q=0.3668, p=0.2438	--	Unpaired t-test	t(12)=1.86, q =0.3396, p=0.0879
maleimide	1743	10935	1,183.50±326.08 (704-1,626; n=8)	1,593.17±310.39 (1,231-2,004; n=6)	1,307.30±242.96 (952-1,577; n=8)	2,310.40±1,379.36 (863-4,815; n=8)	Welch's ANOVA	3.00(3.00, 13.44), q=0.1805, p=0.0680	--	Unpaired t-test	t(12)=2.37, q =0.3167, p=0.0352
malic acid*	1391	525	4,684.00±1,737.87 (1,377-7,652; n=8)	7,237.17±2,195.39 (4,584-10,971; n=6)	9,317.50±3,930.47 (3,776-15,400; n=8)	9,012.50±2,262.07 (5,377-12,465; n=8)	1-way ANOVA	F(3, 26)=4.96, q=0.0466, p=0.0813	LD/LD-V vs. WD/LD-V q=0.0074, p=0.0020; LD/LD-V vs. WD/LD-T q=0.0074, p=0.0035	Unpaired t-test	t(12)=2.43, q =0.3959, p=0.0315
malonic acid	16918	867	94.50±19.91 (73-132; n=8)	95.33±12.72 (70-103; n=6)	124.10±62.47 (72-268; n=8)	72.40±33.41 (12-123; n=8)	Kruskal-Wallis test	Kruskal-Wallis statistic=6.16, q=0.2273, p=0.1043	--	Mann-Whitney test	U=20, q =0.8387, p=0.6364
maltose	1979	439186	73,759.00±31,280.70 (32,599-137,825; n=8)	98,429.67±115,113.84 (5,622-316,526; n=6)	81,149.40±50,736.15 (12,602-163,882; n=8)	142,259.10±154,914.85 (920-436,225; n=8)	Welch's ANOVA	0.52(3.00, 12.08), q=0.7402, p=0.6749	--	Welch's t-test	t(5.56)=0.51, q =0.8367, p=0.6290
maltotriose[LT]	84521	439586	3.18±0.32 (2.73-3.61; n=8)	3.30±0.84 (2.13-4.19; n=6)	3.02±0.62 (2.04-3.66; n=8)	3.14±1.00 (1.49-4.36; n=8)	1-way ANOVA	F(3, 26)=0.18, q=0.9379, p=0.9103	--	Welch's t-test	t(5.31)=1.43, q =0.4597, p=0.2082
mannose[LT]	390222	18950	3.72±0.32 (3.33-4.35; n=8)	3.75±0.30 (3.28-4.03; n=6)	3.86±0.26 (3.50-4.37; n=8)	4.08±0.50 (3.43-5.12; n=8)	1-way ANOVA	F(3, 26)=1.54, q=0.3558, p=0.2281	--	Unpaired t-test on LT data	t(12)=0.14, q =0.9738, p=0.8930
methionine*	45	6137	9,269.25±2,232.44 (5,801-13,928; n=8)	23,571.33±12,213.49 (7,303-35,435; n=6)	21,099.60±9,325.22 (9,722-36,863; n=8)	38,675.60±20,918.42 (14,844-80,478; n=8)	1-way ANOVA	F(3, 26)=6.82, q=0.0194, p=0.0015	LD/LD-V vs. WD/LD-T q=0.0005, p=0.0001; WD/WD-V vs. WD/LD-T q=0.0561, p=0.0422; WD/LD-V vs. WD/LD-T q=0.0261, p=0.0124	Welch's t-test	t(5.25)=2.83, q =0.3460, p=0.0346

Metabolite	BinBase Identifier	PubChem Identifier	LD/LD-V	WD/WD-V	WD/LD-V	WD/LD-T	Test Used	Main Effect of Treatment Group Statistical Result	Study 2 Significant Pairwise Comparisons (with/without correction for multiple comparisons)	WD *vs.* LD only: Test Used	WD *vs.* LD only: Result
methionine sulfoxide	372461	10062737	5,870.38±7,350.19 (2,375-23,984; n=8)	5,019.17±1,403.27 (3,013-6,782; n=6)	5,289.80±2,285.53 (3,539-9,998; n=8)	7,968.30±6,584.90 (3,204-23,023; n=8)	Kruskal-Wallis test	Kruskal-Wallis statistic=4.85, q=0.3178, p=0.1832	--	Mann-Whitney test	U=12, q =0.4159, p=0.1419
methylmaleic acid	18026	643798	1,543.38±1,106.44 (306-3,387; n=8)	686.67±330.71 (268-1,107; n=6)	1,069.00±693.73 (399-2,273; n=8)	750.60±534.93 (194-1,874; n=8)	1-way ANOVA	F(3, 26)=2.06, q=0.2650, p=0.1294	--	Welch's t-test	t(8.60)=2.07, q =0.3207, p=0.0698
monomyristin	97325	79050	291.38±102.60 (87-421; n=8)	554.67±226.54 (328-972; n=6)	487.30±331.87 (294-1,297; n=8)	803.30±794.64 (200-2,579; n=8)	Kruskal-Wallis test	Kruskal-Wallis statistic=8.40, q=0.1145, p=0.0384	--	Unpaired t-test	t(12)=2.94, q =0.2720, p=0.0124
myo-inositol	1741	892	192,379.88±85,793.92 (112,401-371,445; n=8)	252,384.50±57,252.53 (186,853-349,874; n=6)	250,085.90±107,084.66 (115,585-466,920; n=8)	185,013.30±66,988.37 (73,925-249,918; n=8)	1-way ANOVA	F(3, 26)=1.41, q=0.3864, p=0.2614	--	Unpaired t-test	t(12)=1.48, q =0.4328, p=0.1655
myristic acid[LT]	127	11005	4.05±0.12 (3.82-4.18; n=8)	3.98±0.05 (3.94-4.07; n=6)	3.99±0.12 (3.86-4.19; n=8)	3.86±0.11 (3.69-4.03; n=8)	1-way ANOVA	F(3, 26)=4.32, q=0.0560, p=0.0135	--	Unpaired t-test on LT data	t(12)=1.26, q =0.4779, p=0.2305
N-acetylaspartic acid	4081	65065	298.88±651.22 (25-1,909; n=8)	931.67±1,341.30 (43-3,030; n=6)	675.80±1,671.25 (38-4,811; n=8)	1,158.80±1,067.22 (63-2,711; n=8)	Kruskal-Wallis test	Kruskal-Wallis statistic=6.86, q=0.1887, p=0.0766	--	Mann-Whitney test	U=15, q =0.5582, p=0.2824
N-acetylmannosamine[LT]	3244	439281	2.40±0.32 (1.94-2.86; n=8)	2.11±0.12 (1.88-2.23; n=6)	2.28±0.17 (2.04-2.53; n=8)	2.22±0.33 (1.70-2.80; n=8)	1-way ANOVA	F(3, 26)=1.50, q=0.3609, p=0.2378	--	Welch's t-test	t(7.33)=2.19, q =0.3190, p=0.0632
N-carbamoylaspartate	106743	93072	90.75±14.62 (68-106; n=8)	59.67±11.20 (38-71; n=6)	560.10±1,422.05 (28-4,079; n=8)	62.50±27.44 (14-101; n=8)	Kruskal-Wallis test	Kruskal-Wallis statistic=8.11, q=0.1252, p=0.0439	--	Mann-Whitney test	U=2, q =0.0978, p=0.0023
nicotinamide[LT]	84542	936	3.95±0.10 (3.75-4.05; n=8)	3.93±0.20 (3.55-4.12; n=6)	4.01±0.23 (3.51-4.27; n=8)	4.16±0.24 (3.85-4.62; n=8)	1-way ANOVA	F(3, 26)=2.04, q=0.2670, p=0.6151	--	Unpaired t-test	t(12)=0.04, q =0.9822, p=0.9706
nicotinamide-to-nicotinic acid ratio[LT]	--	--	0.86±0.29 (0.55-1.24; n=8)	1.22±0.34 (0.58-1.54; n=6)	1.16±0.25 (0.73-1.46; n=8)	1.54±0.38 (1.08-2.32; n=8)	1-way ANOVA	F (3,26)=6.15, p=0.0027	LD/LD-V vs. WD/WD-V q=0.0794, p=0.0487; LD/LD-V VS. WD/LD-T q=0.0012, p=0.0002; WD/LD-V vs. WD/LD-T q=0.0591, p=0.0225	Unpaired t-test	t(12)=2.62, q =0.0000, p=0.0222
nicotinic acid[LT]*	285	938	3.08±0.28 (2.70-3.47; n=8)	2.71±0.15 (2.59-2.97; n=6)	2.85±0.18 (2.56-3.12; n=8)	2.61±0.29 (2.22-3.15; n=8)	1-way ANOVA	F(3, 26)=5.65, q=0.0349, p=0.0041	LD/LD-V vs. WD/WD-V q=0.0164, p=0.0078; LD/LD-V vs. WD/LD-T q=0.0025, p=0.0006	Unpaired t-test on LT data	t(12)=2.92, q =0.2720, p=0.0128

Supplementary Table 5

Metabolite	BinBase Identifier	PubChem Identifier	LD/LD-V	WD/WD-V	WD/LD-V	WD/LD-T	Test Used	Main Effect of Treatment Group Statistical Result	Study 2 Significant Pairwise Comparisons (with/without correction for multiple comparisons)	WD *vs.* LD only: Test Used	WD *vs.* LD only: Result
nonadecanoic acid	46258	12591	3,045.25±868.83 (1,785-4,488; n=8)	2,484.50±552.16 (1,786-3,440; n=6)	2,712.10±756.31 (1,768-3,994; n=8)	2,061.10±469.40 (1,082-2,707; n=8)	1-way ANOVA	F(3, 26)=2.86, q=0.1538, p=0.0561	--	Unpaired t-test	t(12)=1.38, q =0.4434, p=0.1932
oleamide	20961	5283387	4,989.50±1,488.68 (2,657-6,648; n=8)	5,252.33±1,671.58 (3,725-7,895; n=6)	4,696.60±1,018.48 (3,293-6,404; n=8)	3,607.00±974.32 (2,454-5,119; n=8)	1-way ANOVA	F(3, 26)=2.34, q=0.2243, p=0.0963	--	Unpaired t-test	t(12)=0.31, q =0.9027, p=0.7615
oleic acid	43	445639	50,209.13±49,031.77 (2,105-130,501; n=8)	62,879.67±30,018.72 (2,447-81,147; n=6)	86,217.80±45,909.56 (2,828-156,209; n=8)	100,629.10±25,901.32 (57,420-126,318; n=8)	Kruskal-Wallis test	Kruskal-Wallis statistic=6.19, q=0.2281, p=0.1029	--	Mann-Whitney test	U=18, q =0.7255, p=0.4908
ornithine[LT]	1821	6262	4.40±0.16 (4.14-4.70; n=8)	4.43±0.37 (3.99-5.08; n=6)	4.54±0.12 (4.39-4.78; n=8)	4.68±0.23 (4.40-5.08; n=8)	1-way ANOVA	F(3, 26)=2.54, q=0.1902, p=0.0783	--	Unpaired t-test on LT data	t(12)=0.22, q =0.9529, p=0.8261
orotic acid[LT]	18492	967	1.95±0.50 (1.15-2.71; n=8)	1.89±0.57 (0.90-2.34; n=6)	2.47±0.97 (1.38-4.55; n=8)	2.26±0.28 (1.67-2.53; n=8)	1-way ANOVA	F(3, 26)=1.33, q=0.4077, p=0.2854	--	Unpaired t-test on LT data	t(12)=0.22, q =0.9529, p=0.8296
oxoproline	10	7405	120,700.63±31,691.04 (75,970-161,459; n=8)	146,437.50±28,896.80 (106,438-180,675; n=6)	146,453.10±25,327.12 (94,426-175,835; n=8)	168,777.60±35,547.57 (115,589-206,386; n=8)	1-way ANOVA	F(3, 26)=3.27, q=0.1123, p=0.0370	--	Unpaired t-test	t(12)=1.56, q =0.4172, p=0.1448
palmitic acid	11	985	315,676.88±87,115.63 (204,012-458,076; n=8)	319,426.17±54,707.44 (265,692-391,875; n=6)	312,916.90±60,893.63 (241,264-429,951; n=8)	275,297.60±55,529.24 (212,249-393,489; n=8)	1-way ANOVA	F(3, 26)=0.73, q=0.6381, p=0.5443	--	Unpaired t-test	t(12)=0.09, q =0.9779, p=0.9281
palmitoleic acid[LT*]	391898	445638	4.19±0.20 (3.96-4.53; n=8)	3.88±0.11 (3.76-4.08; n=6)	4.01±0.16 (3.83-4.30; n=8)	3.97±0.12 (3.83-4.17; n=8)	1-way ANOVA	F(3, 26)=5.42, q=0.0386, p=0.0050	LD/LD-V vs. WD/WD-V q=0.0033, p=0.0008; LD/LD-V vs. WD/LD-V q=0.0396, p=0.0283; LD/LD-V vs. WD/LD-T q=0.0141, p=0.0067	Unpaired t-test on LT data	t(12)=3.48, q =0.1530, p=0.0045
pantothenic acid	31356	6613	1,850.88±548.73 (1,022-2,546; n=8)	1,367.33±310.46 (919-1,770; n=6)	1,260.40±470.36 (801-2,071; n=8)	1,379.10±756.38 (452-2,885; n=8)	1-way ANOVA	F(3, 26)=1.75, q=0.3178, p=0.1821	--	Unpaired t-test	t(12)=1.93, q =0.3396, p=0.0779
pentadecanoic acid	1680	13849	16,520.13±3,898.02 (8,992-20,803; n=8)	16,385.33±2,732.62 (12,567-19,408; n=6)	16,953.10±5,559.34 (11,122-27,980; n=8)	15,323.00±6,759.09 (8,047-30,219; n=8)	1-way ANOVA	F(3, 26)=0.15, q=0.9453, p=0.9311	--	Unpaired t-test	t(12)=0.07, q =0.9782, p=0.9437
phenylacetic acid*	1733	999	552.63±540.96 (179-1,425; n=8)	133.50±33.06 (90-183; n=6)	223.10±107.26 (157-485; n=8)	115.10±32.78 (67-175; n=8)	Kruskal-Wallis test	Kruskal-Wallis statistic=21.20, q=0.0043, p<0.0001	LD/LD-V vs. WD/WD-V q=0.0017, p=0.0011; LD/LD-V vs. WD/LD-T q=0.0001, p<0.0001; WD/WD-V vs. WD/LD-V q=0.0304, p=0.0386; WD/LD-V vs. WD/LD-T q=0.0052, p=0.0049	Mann-Whitney test	U=1, q =0.0737, p=0.0013

Metabolite	BinBase Identifier	PubChem Identifier	LD/LD-V	WD/WD-V	WD/LD-V	WD/LD-T	Test Used	Main Effect of Treatment Group Statistical Result	Study 2 Significant Pairwise Comparisons (with/without correction for multiple comparisons)	WD *vs.* LD only: Test Used	WD *vs.* LD only: Result
phenylalanine*	33	6140	18,226.75±8,088.40 (13,097-36,647; n=8)	44,509.50±24,384.25 (11,064-71,459; n=6)	42,351.10±21,380.39 (16,360-76,711; n=8)	80,573.40±49,016.87 (31,019-186,326; n=8)	Kruskal-Wallis test	Kruskal-Wallis statistic=14.66, q=0.0223, p=0.0021	LD/LD-V vs. WD/WD-V q=0.0676, p=0.0386; LD/LD-V vs. WD/LD-V q=0.0676, p=0.0288; LD/LD-V vs. WD/LD-T q=0.0007, p=0.0001	Mann-Whitney test	U=10, q =0.3291, p=0.0813
phosphate	4	1004	248,170.63±84,406.07 (136,056-377,890; n=8)	209,270.17±44,986.14 (133,723-272,054; n=6)	25,4375.40±70,393.50 (149,131-357,871; n=8)	233,744.40±66,171.25 (156,604-356,909; n=8)	1-way ANOVA	F(3, 26)=0.56, q=0.7194, p=0.6475	--	Unpaired t-test	t(12)=1.02, q =0.6273, p=0.3284
phosphoethanolamine	41988	1015	22,338.00±89,13.60 (6,793-31,436; n=8)	25,693.67±5,167.93 (16,551-30,904; n=6)	24,337.10±10,025.11 (10,725-39,762; n=8)	19,047.40±7,912.88 (6,768-30,698; n=8)	1-way ANOVA	F(3, 26)=0.87, q=0.5891, p=0.4713	--	Unpaired t-test	t(12)=0.82, q =0.6681, p=0.4284
phthalic acid[LT]	46142	1017	3.61±0.08 (3.47-3.71; n=8)	3.63±0.10 (3.53-3.82; n=6)	3.72±0.08 (3.63-3.87; n=8)	3.58±0.18 (3.25-3.86; n=8)	1-way ANOVA	F(3, 26)=2.00, q=0.2736, p=0.1384	--	Unpaired t-test on LT data	t(12)=0.38, q =0.8813, p=0.7092
phytol	3192	5280435	3,134.13±1,721.69 (1,657-6,851; n=8)	2,535.33±794.22 (1,831-3,660; n=6)	2,207.50±561.42 (1,683-3,328; n=8)	1,517.90±752.04 (457-2,456; n=8)	1-way ANOVA	F(3, 26)=3.12, q=0.1265, p=0.0432	--	Unpaired t-test	t(12)=0.79, q =0.6889, p=0.4473
pimelic acid	33429	385	1,427.88±359.00 (921-1,825; n=8)	1,537.00±469.55 (1,159-2,291; n=6)	1,239.60±291.68 (941-1,737; n=8)	948.00±339.16 (491-1,641; n=8)	1-way ANOVA	F(3, 26)=3.73, q=0.0806, p=0.0237	--	Unpaired t-test	t(12)=0.49, q =0.8367, p=0.6300
pinitol	115876	164619	1,656.00±1,035.22 (337-2,705; n=8)	1,596.83±1,130.25 (393-3,184; n=6)	1,875.10±1,052.50 (131-3,645; n=8)	1,468.50±1,101.06 (521-4,078; n=8)	Kruskal-Wallis test	Kruskal-Wallis statistic=1.11, q=0.8397, p=0.7755	--	Mann-Whitney test	U=22, q =0.9590, p=0.8518
proline	8	145742	12,622.50±3,924.56 (5,472-16,894; n=8)	15,419.83±9,285.65 (6,403-31,211; n=6)	18,490.10±7,459.82 (9,321-32,375; n=8)	32,307.90±19,332.43 (9,753-72,822; n=8)	1-way ANOVA	F(3, 26)=4.38, q=0.0558, p=0.0127	--	Welch's t-test	t(6.35)=0.69, q =0.7282, p=0.5129
pseudo uridine	1688	15047	318.88±118.37 (172-523; n=8)	848.67±1,498.77 (108-3,905; n=6)	398.10±93.57 (282-564; n=8)	355.60±112.28 (250-585; n=8)	Kruskal-Wallis test	Kruskal-Wallis statistic=4.49, q=0.3480, p=0.2129	--	Mann-Whitney test	U=23, q =0.9800, p=0.9234
putrescine	281	1045	3,161.13±1,638.11 (1,548-6,708; n=8)	3,033.83±2,080.15 (1,881-7,183; n=6)	3,760.00±1,619.49 (1,484-6,607; n=8)	2,706.80±1,276.47 (1,010-4,587; n=8)	Kruskal-Wallis test	Kruskal-Wallis statistic=2.24, q=0.6229, p=0.5240	--	Mann-Whitney test	U=17, q =0.6571, p=0.4136
pyruvic acid[LT]	5864	1060	3.34±0.10 (3.23-3.51; n=8)	3.28±0.18 (3.05-3.46; n=6)	3.31±0.08 (3.23-3.45; n=8)	3.26±0.23 (2.97-3.61; n=8)	1-way ANOVA	F(3, 26)=0.44, q=0.7908, p=0.7257	--	Unpaired t-test	t(12)=0.67, q =0.7282, p=0.5140

Metabolite	BinBase Identifier	PubChem Identifier	LD/LD-V	WD/WD-V	WD/LD-V	WD/LD-T	Test Used	Main Effect of Treatment Group Statistical Result	Study 2 Significant Pairwise Comparisons (with/without correction for multiple comparisons)	WD *vs.* LD only: Test Used	WD *vs.* LD only: Result
ribitol	7362	827	367.00±130.45 (196-631; n=8)	594.50±221.73 (301-903; n=6)	665.60±312.87 (300-1,345; n=8)	1,098.30±1,181.29 (464-3,983; n=8)	Kruskal-Wallis test	Kruskal-Wallis statistic=10.95, q=0.0561, p=0.0120	--	Unpaired t-test	t(12)=2.42, q =0.3751, p=0.0326
ribonic acid[LT]	84543	5460677	2.70±0.20 (2.35-3.04; n=8)	2.93±0.25 (2.60-3.24; n=6)	2.81±0.20 (2.57-3.06; n=8)	2.83±0.37 (2.36-3.58; n=8)	1-way ANOVA	F(3, 26)=0.91, q=0.5762, p=0.4508	--	Unpaired t-test	t(12)=1.96, q =0.3311, p=0.0740
ribulose-5-phosphate	42027	439184	263.50±78.94 (103-332; n=8)	613.50±313.63 (200-998; n=6)	540.60±283.82 (218-1,054; n=8)	689.40±554.89 (240-1,882; n=8)	Kruskal-Wallis test	Kruskal-Wallis statistic=8.52, q=0.1134, p=0.0364	--	Welch's t-test	t(5.48)=2.67, q =0.3008, p=0.0404
salicylaldehyde	168865	6998	477.13±138.47 (242-725; n=8)	324.67±115.55 (162-488; n=6)	606.50±433.19 (255-1,262; n=8)	208.60±87.41 (84-316; n=8)	Kruskal-Wallis test	Kruskal-Wallis statistic=11.42, q=0.0526, p=0.0096	--	Unpaired t-test	t(12)=2.18, q =0.3256, p=0.0498
salicylic acid*	3063	338	1,935.88±1,707.85 (795-5,187; n=8)	518.33±165.27 (391-756; n=6)	801.00±445.89 (449-1,792; n=8)	316.50±119.46 (136-420; n=8)	Kruskal-Wallis test	Kruskal-Wallis statistic=20.75, q=0.0034, p=0.0001	LD/LD-V vs. WD/WD-V q=0.0048, p=0.0046; LD/LD-V vs. WD/LD-T q<0.0001, p<0.0001; WD/LD-V vs. WD/LD-T q=0.0048, p=0.0036	Mann-Whitney test	U=0, q =0.0595, p=0.0007
serine	25	5951	94,659.13±18,772.78 (61,395-125,518; n=8)	143,646.67±80,217.23 (48,642-254,415; n=6)	128,492.80±30,043.12 (81,668-167,493; n=8)	182,112.60±81,682.08 (92,693-348,175; n=8)	Welch's ANOVA	4.54(3.00, 12.73), q=0.0814, p=0.0224	--	Welch's t-test	t(5.41)=1.47, q =0.4433, p=0.1982
sophorose[LT]	132242	441432	2.19±0.52 (1.23-2.76; n=8)	2.22±0.46 (1.70-2.92; n=6)	2.62±0.21 (2.20-2.93; n=8)	2.42±0.52 (1.77-3.27; n=8)	1-way ANOVA	F(3, 26)=1.54, q=0.3558, p=0.2279	--	Unpaired t-test on LT data	t(12)=0.12, q =0.9807, p=0.9057
sorbitol	162	5780	2,691.75±3,897.65 (479-12,205; n=8)	2,130.50±932.68 (1,202-3,837; n=6)	2,065.40±848.96 (1,233-3,120; n=8)	1,800.50±1,104.33 (591-4,204; n=8)	Kruskal-Wallis test	Kruskal-Wallis statistic=1.89, q=0.6836, p=0.5951	--	Unpaired t-test on LT data	t(12)=0.54, q =0.8230, p=0.6003
stearic acid	13	5281	2,118,986.13±601,716.56 (1,067,425-2,647,683; n=8)	2,127,267.00±602,388.75 (1,651,467-3,001,788; n=6)	1,938,831.00±395,694.02 (1,624,937-2,704,739; n=8)	1,375,691.30±541,630.17 (675,132-2,414,807; n=8)	Kruskal-Wallis test	Kruskal-Wallis statistic=9.31, q=0.0847, p=0.0254	--	Mann-Whitney test	U=22, q =0.9590, p=0.8518
succinate semialdehyde	2226	1112	1,935.63±795.71 (1,031-3,460; n=8)	1,738.33±750.50 (919-2,598; n=6)	1,277.60±348.86 (869-1,841; n=8)	1,029.00±365.35 (596-1,770; n=8)	1-way ANOVA	F(3, 26)=3.85, q=0.0790, p=0.0209	--	Unpaired t-test	t(12)=0.47, q =0.8338, p=0.6468
succinic acid[LT]	161	1110	3.48±0.14 (3.28-3.65; n=8)	3.47±0.16 (3.19-3.61; n=6)	3.55±0.10 (3.39-3.73; n=8)	3.29±0.25 (3.02-3.77; n=8)	1-way ANOVA	F(3, 26)=3.28, q=0.1123, p=0.0367	--	Unpaired t-test	t(12)=0.10, q =0.9811, p=0.9185

Metabolite	BinBase Identifier	PubChem Identifier	LD/LD-V	WD/WD-V	WD/LD-V	WD/LD-T	Test Used	Main Effect of Treatment Group Statistical Result	Study 2 Significant Pairwise Comparisons (with/without correction for multiple comparisons)	WD *vs.* LD only: Test Used	WD *vs.* LD only: Result
sucrose	173	5988	4,450.75±3,822.16 (1,579-10,610; n=8)	3,550.17±4,736.87 (920-12,824; n=6)	9,705.60±13,317.70 (1,060-32,014; n=8)	1,831.80±1,190.62 (895-4,497; n=8)	Kruskal-Wallis test	Kruskal-Wallis statistic=6.02, q=0.2348, p=0.1105	--	Mann-Whitney test	U=13, q =0.4401, p=0.1812
tartaric acid	33985	444305	92.00±53.69 (17-155; n=8)	371.33±611.35 (83-1,618; n=6)	188.30±151.26 (65-521; n=8)	149.40±75.34 (48-280; n=8)	Kruskal-Wallis test	Kruskal-Wallis statistic=3.90, q=0.3956, p=0.2723	--	Mann-Whitney test	U=14, q =0.4779, p=0.2284
terephthalic acid	46252	7489	2,995.13±970.21 (1,862-4,313; n=8)	2,677.50±664.89 (2,010-3,912; n=6)	2,942.10±495.93 (2,456-3,808; n=8)	1,979.00±769.45 (815-2,917; n=8)	1-way ANOVA	F(3, 26)=3.10, q=0.1252, p=0.0442	--	Unpaired t-test	t(12)=0.69, q =0.7348, p=0.5053
threonic acid*	172	5460407	2,765.50±1,050.38 (1,402-4,627; n=8)	7,609.50±3,811.13 (2,842-12,612; n=6)	7,910.60±4,126.15 (2,480-14,476; n=8)	8,273.60±3525.86 (3,421-12,859; n=8)	1-way ANOVA	F(3, 26)=4.84, q=0.0470, p=0.0083	LD/LD-V vs. WD/WD-V q=0.0126, p=0.0120; LD/LD-V vs. WD/LD-V q=0.0073, p=0.0046; LD/LD-V vs. WD/LD-T q=0.0073, p=0.0027	Welch's t-test	t(5.57)=3.03, q =0.3825, p=0.0254
threonine	26	6288	13,027.00±4,288.38 (6,217-19,264; n=8)	18,494.00±7,738.96 (7,700-26,067; n=6)	19,504.90±6,557.68 (9,160-28,464; n=8)	27,548.10±11,457.62 (15,139-50,355; n=8)	1-way ANOVA	F(3, 26)=4.51, q=0.0560, p=0.0112	--	Unpaired t-test	t(12)=1.69, q =0.4021, p=0.1159
tocopherol alpha-	100	638015	12,832.88±6,531.61 (5,822-20,455; n=8)	5,545.50±2,636.62 (2,441-9,708; n=6)	10,270.60±5,837.91 (2,793-21,093; n=8)	7,426.10±5,043.68 (743-13,679; n=8)	Kruskal-Wallis test	Kruskal-Wallis statistic=5.46, q=0.2755, p=0.1410	--	Mann-Whitney test	U=8, q =0.3018, p=0.0426
tocopherol gamma-	4545	92729	516.75±465.86 (169-1,595; n=8)	524.67±211.27 (322-743; n=6)	696.50±246.47 (433-1,224; n=8)	537.60±366.54 (84-1,039; n=8)	Kruskal-Wallis test	Kruskal-Wallis statistic=3.49, q=0.4492, p=0.3224	--	Mann-Whitney test	U=16, q =0.6046, p=0.3450
trans-4-hydroxyproline	43180	5810	845.75±218.44 (530-1,178; n=8)	904.83±291.00 (508-1,195; n=6)	1,018.10±272.24 (563-1,465; n=8)	1,047.30±201.72 (692-1,347; n=8)	1-way ANOVA	F(3, 26)=1.17, q=0.4712, p=0.3409	--	Unpaired t-test	t(12)=0.44, q =0.8513, p=0.6710
tryptophan^{LT}*	14	6305	4.02±0.10 (3.84-4.14; n=8)	4.26±0.34 (3.69-4.55; n=6)	4.33±0.19 (4.01-4.58; n=8)	4.61±0.26 (4.22-5.09; n=8)	1-way ANOVA	F(3, 26)=9.17, q=0.0064, p=0.0003	LD/LD-V vs. WD/WD-V q=0.0266, p=0.0632; LD/LD-V vs. WD/LD-V q=0.0075, p=0.0106; LD/LD-V vs. WD/LD-T q<0.0001, p<0.0001; WD/WD-V vs. WD/LD-T q=0.0075; p=0.0079; WD/LD-V vs. WD/LD-T q=0.0111, p=0.0211	Welch's t-test	t(5.25)=2.30, q =0.3192, p=0.0676

Metabolite	BinBase Identifier	PubChem Identifier	LD/LD-V	WD/WD-V	WD/LD-V	WD/LD-T	Test Used	Main Effect of Treatment Group Statistical Result	Study 2 Significant Pairwise Comparisons (with/without correction for multiple comparisons)	WD *vs.* LD only: Test Used	WD *vs.* LD only: Result
tyrosine^{LT*}	16	6057	4.47±0.13 (4.28-4.76; n=8)	4.80±0.34 (4.25-5.09; n=6)	4.81±0.22 (4.48-5.10; n=8)	5.11±0.25 (4.71-5.54; n=8)	1-way ANOVA	F(3, 26)=9.61, q=0.0049, p=0.0002	LD/LD-V vs. WD/WD-V q=0.0047, p=0.0174; LD/LD-V vs. WD/LD-V q=0.0043, p=0.0082; LD/LD-V vs. WD/LD-T q<0.0001, p<0.0001; WD/WD-V vs. WD/LD-T q=0.0047, p=0.0224; WD/LD-V vs. WD/LD-T q=0.0047, p=0.0189	Welch's t-test on LT data	t(6.20)=2.22, q =0.3192, p=0.0666
UDP-N-acetylglucosamine	62293	445675	1,617.25±529.42 (866-2,170; n=8)	1,857.33±354.18 (1,304-2,285; n=6)	1,644.30±388.47 (1,100-2,132; n=8)	1,360.10±559.36 (582-2,331; n=8)	1-way ANOVA	F(3, 26)=1.30, q=0.4188, p=0.2956	--	Unpaired t-test	t(12)=0.96, q =0.6076, p=0.3574
uracil	1664	1174	5,607.75±1,933.34 (2,403-8,582; n=8)	4,615.83±1,740.80 (3,044-7,216; n=6)	6,173.30±2,062.86 (3,567-8,424; n=8)	5,601.40±2,283.40 (1,607-8,623; n=8)	1-way ANOVA	F(3, 26)=0.68, q=0.6693, p=0.5748	--	Unpaired t-test	t(12)=0.99, q =0.6120, p=0.3418
urea	3256	1176	297,485.13±155,631.97 (135,352-589,988; n=8)	289,948.50±315,340.06 (107,713-929,277; n=6)	240,246.50±59,824.03 (162,205-350,900; n=8)	180,513.90±104,107.66 (113,701-353,078; n=8)	Kruskal-Wallis test	Kruskal-Wallis statistic=6.26, q=0.2283, p=0.0996	--	Mann-Whitney test	U=16, q =0.6046, p=0.3450
uric acid	23	1175	2,235.63±1,028.31 (1,042-4,166; n=8)	4,025.67±4,204.05 (207-11,968; n=6)	3,494.80±2,262.40 (360-6,802; n=8)	4,854.00±3,050.44 (1,938-11,111; n=8)	1-way ANOVA	F(3, 26)=1.26, q=0.4331, p=0.3083	--	Welch's t-test	t(5.45)=1.02, q =0.6024, p=0.3507
uridine*	4705	6029	499.25±295.68 (91-919; n=8)	652.00±471.50 (40-1,177; n=6)	1,317.10±1,018.05 (99-3,432; n=8)	1,969.00±944.81 (494-3,745; n=8)	1-way ANOVA	F(3, 26)=5.95, q=0.0277, p=0.0031	LD/LD-V vs. WD/LD-V q=0.0590, p=0.0421, LD/LD-V vs. WD/LD-T q=0.0030, p=0.0007; WD/WD-V vs. WD/LD-T q=0.0078, p=0.0037	Unpaired t-test	t(12)=0.75, q =0.7068, p=0.4698
valine^{LT}*	3	6287	4.55±0.13 (4.36-4.76; n=8)	4.82±0.25 (4.46-5.03; n=6)	4.85±0.16 (4.68-5.08; n=8)	5.00±0.19 (4.78-5.30; n=8)	1-way ANOVA	F(3, 26)=8.38, q=0.0085, p=0.0005	LD/LD-V vs. WD/WD-V q=0.0118, p=0.0113; LD/LD-V vs. WD/LD-V q=0.0044, p=0.0028; LD/LD-V vs. WD/LD-T q=0.0001, p<0.0001	Welch's t-test	t(5.81)=2.62, q =0.3008, p=0.0407
xanthine*	1669	1188	1,443.13±1,168.78 (143-3,558; n=8)	3,351.33±2,797.43 (77-7,227; n=6)	4,692.50±2,849.65 (232-7,878; n=8)	11,670.30±6,587.79 (639-21,006; n=8)	1-way ANOVA	F(3, 26)=9.93, q=0.0049, p=0.0002	LD/LD-V vs WD/LD-T q<0.0001, p<0.0001; WD/WD-V vs. WD/LD-T q=0.0010, p=0.0006; WD/LD-V vs WD/LD-T q=0.0017, p=0.0016	Unpaired t-test	t(12)=1.75, q =0.3715, p=0.1049

Metabolite	BinBase Identifier	PubChem Identifier	LD/LD-V	WD/WD-V	WD/LD-V	WD/LD-T	Test Used	Main Effect of Treatment Group Statistical Result	Study 2 Significant Pairwise Comparisons (with/without correction for multiple comparisons)	WD *vs.* LD only: Test Used	WD *vs.* LD only: Result
xanthosine[LT]	104466	64959	2.15±0.22 (1.73-2.49; n=8)	2.21±0.27 (1.92-2.56; n=6)	2.32±0.30 (1.83-2.73; n=8)	2.58±0.27 (2.26-3.16; n=8)	1-way ANOVA	F(3, 26)=3.92, q=0.0757, p=0.0196	--	Unpaired t-test on LT data	t(12)=0.52, q =0.8306, p=0.6107
xylitol	5857	6912	1,138.50±371.39 (714-1,857; n=8)	1,517.00±685.67 (1,035-2,891; n=6)	1,477.10±414.84 (1,032-2,265; n=8)	1,500.00±1,003.60 (908-3,933; n=8)	Kruskal-Wallis test	Kruskal-Wallis statistic=4.02, q=0.3864, p=0.2593	--	Mann-Whitney test	U=12, q =0.4159, p=0.1419
xylose[LT]	169	135191	3.01±0.07 (2.89-3.12; n=8)	2.98±0.18 (2.80-3.31; n=6)	3.01±0.08 (2.90-3.17; n=8)	2.92±0.18 (2.60-3.24; n=8)	1-way ANOVA	F(3, 26)=0.79, q=0.6148, p=0.5099	--	Welch's t-test on LT data	t(6.31)=0.38, q =0.8784, p=0.7182
xylulose[LT]*	31632	439205	2.58±0.13 (2.35-2.72; n=8)	2.67±0.14 (2.49-2.85; n=6)	2.76±0.12 (2.64-2.97; n=8)	2.94±0.24 (2.60-3.46; n=8)	1-way ANOVA	F(3, 26)=6.78, q=0.0194, p=0.0016	LD/LD-V vs. WD/LD-V q=0.0458, p=0.0327; LD/LD-V vs. WD/LD-T q=0.0008, p=0.0002; WD/WD-V vs. WD/LD-T q=0.0126, p=0.0060; WD/LD-V vs. WD/LD-T q=0.0483, p=0.0460	Unpaired t-test	t(12)=1.42, q =0.4530, p=0.1804
zymosterol[LT]	110304	92746	3.21±0.21 (2.91-3.59; n=8)	3.48±0.19 (3.26-3.70; n=6)	3.46±0.34 (3.09-4.20; n=8)	3.33±0.24 (2.84-3.61; n=8)	1-way ANOVA	F(3, 26)=1.82, q=0.3065, p=0.1677	--	Unpaired t-test	t(12)=2.34, q =0.3205, p=0.0377

Supplemental Table 6 IPA Categorization of Epididymal White Adipose Tissue Metabolites. IPA Disease & Functions (**a**) and Canonical Pathways (**b**) with significant (≥2.000 or ≤-2.000) activation z-scores for the respective comparisons of the log_2 fold changes of epididymal white adipose tissue metabolites between the WD/LD-T vs. WD/LD-V and the WD/WD-V vs. LD/LD-V groups; nine metabolites were unable to map into IPA (see text for list). LD, lean diet; -T, treated with the nicotinamide N-methyltransferase inhibitor; -V, vehicle-treated; WD, Western diet

a. Diseases & Functions

Groups	Disease or Function	p-value	Predicted Activation State	Activation z-score	# of Molecules	Molecules
WD/LD-T vs. WD/LD-V	Uptake of amino acids	3.11E-06	Decreased	-3.24	17	beta-alanine, cholesterol, GABA, glycine, guanosine, L-alanine, L-aspartic acid, L-cysteine, L-glutamic acid, L-methionine, L-phenylalanine, L-serine, L-threonine, L-tryptophan, L-tyrosine, S-2-amino-hexanedioic acid, trans-4-hydroxy-L-proline
	Uptake of L-amino acid	2.87E-07	Decreased	-3.066	16	beta-alanine, cholesterol, GABA, glycine, guanosine, L-alanine, L-aspartic acid, L-cysteine, L-methionine, L-phenylalanine, L-serine, L-threonine, L-tryptophan, L-tyrosine, S-2-amino-hexanedioic acid, trans-4-hydroxy-L-proline
	lines	2.42E-04	Decreased	-2.97	9	arachidonic acid, ascorbic acid, D-sphingosine, glycine, L-cystine, L-methionine, palmitic acid, sorbitol, sucrose
	Cell death of tumor cell lines	8.49E-08	Decreased	-2.932	35	adenosine, AMP, arachidonic acid, ascorbic acid, cholesterol, citric acid, D-sphingosine, ethanolamine, GABA, glycine, inositol, L-cystine, L-glutamic acid, L-glutamine, L-methionine, L-phenylalanine, L-serine, L-tryptophan, L-tyrosine, lactic acid, linoleic acid, malonic acid, myristic acid, oleic acid, orotic acid, palmitic acid, phenylacetic acid, phosphorylethanolamine, pyruvic acid, salicylic acid, sorbitol, stearic acid, sucrose, uridine, xanthine
	Uptake of glutamine family amino acid	1.92E-05	Decreased	-2.714	11	cholesterol, GABA, glycine, guanosine, L-alanine, L-aspartic acid, L-cysteine, L-phenylalanine, L-tryptophan, S-2-amino-hexanedioic acid, trans-4-hydroxy-L-proline
	Uptake of L-proline	4.18E-05	Decreased	-2.646	7	GABA, glycine, L-alanine, L-cysteine, L-phenylalanine, L-tryptophan, trans-4-hydroxy-L-proline
	Apoptosis of lymphoma cell lines	2.15E-03	Decreased	-2.621	7	arachidonic acid, ascorbic acid, D-sphingosine, L-cystine, L-methionine, sorbitol, sucrose
	Response of liver	1.29E-02	Decreased	-2.599	8	ascorbic acid, cholesterol, glycine, inosine, L-methionine, oleic acid, palmitic acid, salicylic acid
	Synthesis of lipid	5.87E-03	Decreased	-2.566	23	adenosine, arachidonic acid, ascorbic acid, cholesterol, cholic acid, D-sphingosine, fumaric acid, GABA, glycerol, hypoxanthine, L-glutamic acid, L-methionine, linoleic acid, myristic acid, nicotinic acid, oleic acid, orotic acid, palmitic acid, phenylacetic acid, phytol, salicylic acid, stearic acid, uric acid
	Uptake of L-alanine	8.99E-08	Decreased	-2.53	10	beta-alanine, glycine, L-alanine, L-cysteine, L-methionine, L-phenylalanine, L-serine, L-threonine, L-tryptophan, L-tyrosine
	Necrosis	1.75E-07	Decreased	-2.46	49	5-aminovaleric acid, adenosine, AMP, arachidonic acid, ascorbic acid, cholesterol, cholic acid, citric acid, D-sphingosine, ethanolamine, GABA, glycerol, glycine, guanosine, inosine, inositol, L-cysteine, L-cystine, L-glutamic acid, L-glutamine, L-methionine, L-phenylalanine, L-serine, L-tryptophan, L-tyrosine, lactic acid, lauric acid, linoleic acid, linolenic acid, malic acid, malonic acid, myristic acid, niacinamide, oleic acid, orotic acid, palmitic acid, phenylacetic acid, phosphorylethanolamine, putrescine, pyruvic acid, retinaldehyde, salicylic acid, sorbitol, stearic acid, succinic acid, sucrose, uric acid, uridine, xanthine
	Cell death of liver cells	8.56E-03	Decreased	-2.356	10	arachidonic acid, ascorbic acid, cholesterol, cholic acid, glycine, L-cysteine, oleic acid, palmitic acid, salicylic acid, stearic acid
	Apoptosis of tumor cell lines	1.28E-07	Decreased	-2.351	29	adenosine, arachidonic acid, ascorbic acid, cholesterol, citric acid, D-sphingosine, ethanolamine, GABA,L-cystine, L-glutamic acid, L-methionine, L-phenylalanine, L-serine, L-tyrosine, linoleic acid, malonic acid, myristic acid, oleic acid, orotic acid, palmitic acid, phenylacetic acid, phosphorylethanolamine, pyruvic acid, salicylic acid, sorbitol, stearic acid, sucrose, uridine, xanthine
	Cell death of epithelial cells	1.25E-02	Decreased	-2.331	13	arachidonic acid, ascorbic acid, cholic acid, glycine, L-cysteine, L-glutamic acid, niacinamide, oleic acid, palmitic acid, retinaldehyde, salicylic acid, sorbitol, stearic acid
	Synthesis of fatty acid	1.55E-03	Decreased	-2.291	17	adenosine, arachidonic acid, ascorbic acid, cholesterol, cholic acid, D-sphingosine, fumaric acid, GABA,glycerol, L-glutamic acid, L-methionine, linoleic acid, nicotinic acid, oleic acid, palmitic acid, salicylic acid, stearic acid
	Liver lesion	1.48E-11	Decreased	-2.284	36	1,5-anhydroglucitol, aminomalonic acid, arachidonic acid, ascorbic acid, cholesterol, cholic acid, D-mannose, ethanolamine, fumaric acid, glycine, inosine, L-cysteine, L-glutamic acid, L-methionine, L-phenylalanine, L-serine, L-threonine, L-tryptophan, L-tyrosine, L-valine, lactic acid, linoleic acid, malic acid, myristic acid, niacinamide, oleic acid, palmitic acid, phosphoric acid, phosphorylethanolamine, pyrrolidonecarboxylic acid, ribitol, salicylic acid, stearic acid, succinic acid, succinic semialdehyde, uric acid

a. Diseases & Functions

WD/LD-T vs. WD/LD-V	Glucose metabolism disorder	8.25E-05	Decreased	-2.254	22	1,5-anhydroglucitol, arachidonic acid, ascorbic acid, cholesterol, citric acid, creatinine, D-pinitol, fumaric acid, GABA, glycerol, glycine, L-aspartic acid, L-glutamic acid, lactic acid, nicotinic acid, oleic acid, palmitic acid, pyruvic acid, salicylic acid, succinic acid, sucrose, uric acid
	Transport of monosaccharide	9.25E-08	Decreased	-2.225	15	ascorbic acid, cholesterol, D-mannose, L-cysteine, L-lysine, L-methionine, L-phenylalanine, L-threonine, L-tryptophan, L-tyrosine, myristic acid, oleic acid, palmitic acid, sorbitol, stearic acid
	Cell death of breast cancer cell lines	1.93E-03	Decreased	-2.212	8	adenosine, arachidonic acid, L-cystine, myristic acid, oleic acid, palmitic acid, pyruvic acid, stearic acid
	Apoptosis of embryonic cell lines	4.49E-03	Decreased	-2.2	5	arachidonic acid, L-cysteine, malonic acid, palmitic acid, sorbitol
	Cell death of hepatocytes	1.13E-02	Decreased	-2.162	9	arachidonic acid, ascorbic acid, cholic acid, glycine, L-cysteine, oleic acid, palmitic acid, salicylic acid, stearic acid
	Necrosis of liver	3.37E-03	Decreased	-2.112	11	arachidonic acid, ascorbic acid, cholesterol, cholic acid, glycine, L-cysteine, L-phenylalanine, oleic acid, palmitic acid, salicylic acid, stearic acid
	Organ Degeneration	2.95E-03	Decreased	-2.044	12	arachidonic acid, ascorbic acid, beta-glycerophosphoric acid, erythritol, L-glutamic acid, L-glutamine, L-phenylalanine, L-valine, malonic acid, niacinamide, palmitic acid, sucrose
	Cell death of fibroblast cell lines	3.92E-05	Decreased	-2.042	11	arachidonic acid, D-sphingosine, L-cysteine, linoleic acid, malonic acid, oleic acid, palmitic acid, pyruvic acid, salicylic acid, sorbitol, sucrose
	Transport of D-glucose	1.83E-07	Decreased	-2.04	14	cholesterol, D-mannose, L-cysteine, L-lysine, L-methionine, L-phenylalanine, L-threonine, L-tryptophan, L-tyrosine, myristic acid, oleic acid, palmitic acid, sorbitol, stearic acid
	Killing of Yersinia pestis	2.14E-05	Decreased	-2	4	lauric acid, linoleic acid, myristic acid, palmitic acid
	Cytolysis of macrophage cancer cell lines	6.35E-04	Decreased	-2	4	ascorbic acid, cholesterol, L-methionine, L-phenylalanine
	Quantity of lactic acid	2.44E-03	Decreased	-2	5	AMP, L-glutamic acid, malonic acid, niacinamide, sucrose
	Weight loss	9.51E-03	Decreased	-2	5	ascorbic acid, creatinine, L-glutamic acid, L-methionine, sucrose
	Efflux of neutral amino acid	3.85E-07	Increased	2.132	8	beta-alanine, glycine, L-alanine, L-cysteine, L-leucine, L-methionine, L-serine, L-threonine
	Interphase	1.37E-06	Increased	2.224	17	arachidonic acid, cholesterol, citric acid, glycine, L-alanine, L-asparagine, L-aspartic acid, L-glutamic acid, L-proline, L-serine, linoleic acid, linolenic acid, niacinamide, phosphorylethanolamine, salicylic acid, stearic acid, uridine
	Viral Infection	2.99E-04	Increased	2.224	21	adenine, adenosine, arachidonic acid, ascorbic acid, cholesterol, citrulline, creatinine, D-mannose, D-sphingosine, guanosine, hypoxanthine, inosine, L-tryptophan, lauric acid, linoleic acid, nicotinic acid, pyruvic acid, salicylic acid, sorbitol, sucrose, urea
	Stimulation of cells	4.24E-04	Increased	2.289	13	arachidonic acid, ascorbic acid, GABA, glycine, L-aspartic acid, L-cysteine, L-glutamic acid, L-proline, L-serine, L-tryptophan, lactic acid, palmitic acid, uric acid
	Cell death of cancer cells	3.21E-03	Increased	2.395	9	adenosine, ascorbic acid, guanosine,inosine,L-glutamic acid,niacinamide,palmitic acid,putrescine,uridine
	Excitation of orexin neurons	3.26E-05	Increased	2.449	6	glycine, L-aspartic acid, L-cysteine, L-proline, L-serine, lactic acid
	Cell viability of hepatoma cell lines	1.03E-04	Increased	2.556	8	arachidonic acid, L-aspartic acid, L-cystine, L-methionine, L-proline, L-serine, palmitic acid, salicylic acid
	Incorporation of thymidine	1.71E-03	Increased	2.631	7	L-methionine, lauric acid, linoleic acid, myristic acid, oleic acid, palmitic acid, stearic acid
	Entry into S phase of hepatocytes	6.18E-09	Increased	2.646	7	glycine, L-alanine, L-asparagine, L-aspartic acid, L-glutamic acid, L-proline, L-serine
	Growth of Yersinia pestis	5.52E-06	Increased	2.821	11	AMP, D-pantothenic acid, glycine, itaconic acid, L-aspartic acid, L-glutamic acid, L-isoleucine, L-methionine, L-phenylalanine, L-threonine, L-valine
	Excitation of neurons	6.45E-04	Increased	2.822	8	GABA, glycine, L-aspartic acid, L-cysteine, L-glutamic acid, L-proline, L-serine, lactic acid
	S phase	9.25E-06	Increased	2.828	9	citric acid, glycine, L-alanine, L-asparagine, L-aspartic acid, L-glutamic acid, L-proline, L-serine, uridine

a. Diseases & Functions

WD/LD-T vs. WD/LD-V	Growth of organism	5.89E-13	Increased	3.043	32	1-oleoylglycerol, adenosine, AMP, ascorbic acid, D-pantothenic acid, erythritol, GABA, glycerol, glycine, hypoxanthine, inosine, itaconic acid, L-alanine, L-aspartic acid, L-cysteine, L-cystine, L-glutamic acid, L-isoleucine, L-methionine, L-phenylalanine, L-proline, L-serine, L-threonine, L-valine, lactic acid, lauric acid, oleic acid, pyruvic acid, sorbitol, uracil, uric acid, uridine
	Growth of bacteria	8.97E-12	Increased	3.078	26	1-oleoylglycerol, adenosine, AMP, ascorbic acid, D-pantothenic acid, erythritol, glycine, hypoxanthine, inosine, itaconic acid, L-alanine, L-aspartic acid, L-cysteine, L-cystine, L-glutamic acid, L-isoleucine, L-methionine, L-phenylalanine, L-proline, L-serine, L-threonine, L-valine, lactic acid, lauric acid, oleic acid, uracil
WD/WD-V vs. LD/LD-V	Uptake of amino acids	2.88E-06	Decreased	-3.63	17	beta-alanine, cholesterol, GABA, glycine, guanosine, L-alanine, L-aspartic acid, L-cysteine, L-glutamic acid, L-methionine, L-phenylalanine, L-serine, L-threonine, L-tryptophan, L-tyrosine, S-2-amino-hexanedioic acid, trans-4-hydroxy-L-proline
	Uptake of L-amino acid	2.17E-07	Decreased	-3.509	16	beta-alanine, cholesterol, GABA, glycine, guanosine, L-alanine, L-aspartic acid, L-cysteine, L-methionine, L-phenylalanine, L-serine, L-threonine, L-tryptophan, L-tyrosine, S-2-amino-hexanedioic acid, trans-4-hydroxy-L-proline
	Uptake of L-alanine	7.41E-08	Decreased	-3.162	10	beta-alanine, glycine, L-alanine, L-cysteine, L-methionine, L-phenylalanine, L-serine, L-threonine, L-tryptophan, L-tyrosine
	Uptake of glutamine family amino acid	1.58E-05	Decreased	-2.714	11	cholesterol, GABA, glycine, guanosine, L-alanine, L-aspartic acid, L-cysteine, L-phenylalanine, L-tryptophan, S-2-amino-hexanedioic acid, trans-4-hydroxy-L-proline
	Uptake of L-proline	3.66E-05	Decreased	-2.646	7	GABA, glycine, L-alanine, L-cysteine, L-phenylalanine, L-tryptophan, trans-4-hydroxy-L-proline
	Oxidation of monosaccharide	1.19E-03	Decreased	-2.6	7	lactic acid, lauric acid, linoleic acid, myristic acid, oleic acid, palmitic acid, stearic acid
	Transport of monosaccharide	7.04E-08	Decreased	-2.578	15	ascorbic acid, cholesterol, D-mannose, L-cysteine, L-lysine, L-methionine, L-phenylalanine, L-threonine, L-tryptophan, L-tyrosine, myristic acid, oleic acid, palmitic acid, sorbitol, stearic acid
	Transport of D-glucose	1.42E-07	Decreased	-2.456	14	cholesterol, D-mannose, L-cysteine, L-lysine, L-methionine, L-phenylalanine, L-threonine, L-tryptophan, L-tyrosine, myristic acid, oleic acid, palmitic acid, sorbitol, stearic acid
	Oxidation of glucose-6-phosphate	2.08E-06	Decreased	-2.449	6	lauric acid, linoleic acid, myristic acid, oleic acid, palmitic acid, stearic acid
	Dermatitis	1.09E-02	Decreased	-2.194	6	azelaic acid, glycerol, L-glutamine, lactic acid, salicylic acid, urea
	Killing of Yersinia pestis	1.97E-05	Increased	2	4	lauric acid, linoleic acid, myristic acid, palmitic acid
	Transport of H+	4.27E-04	Increased	2	5	L-glutamic acid, linoleic acid, linolenic acid, oleic acid, palmitic acid
	Growth of bacteria	5.12E-10	Increased	2.013	23	adenosine, AMP, ascorbic acid, D-pantothenic acid, erythritol, glycine, inosine, itaconic acid, L-alanine, L-aspartic acid, L-cysteine, L-cystine, L-glutamic acid, L-isoleucine, L-methionine, L-phenylalanine, L-proline, L-serine, L-threonine, L-valine, lactic acid, lauric acid, oleic acid
	Formation of fibrils	1.36E-04	Increased	2.02	7	arachidonic acid, ascorbic acid, beta-lactose, oleic acid, palmitic acid, stearic acid, urea
	Concentration of D-glucose	4.92E-05	Increased	2.025	15	AMP, ascorbic acid, cholic acid, glycerol, glycine, L-cysteine, L-glutamine, L-ornithine, lactic acid, levan, niacinamide, oleic acid, pyruvic acid, salicylic acid, sucrose
	Growth of Yersinia pestis	4.52E-06	Increased	2.032	11	AMP, D-pantothenic acid, glycine, itaconic acid, L-aspartic acid, L-glutamic acid, L-isoleucine, L-methionine, L-phenylalanine, L-threonine, L-valine
	Generation of reactive oxygen species	1.47E-04	Increased	2.038	19	adenosine, arachidic acid, arachidonic acid, ascorbic acid, cholesterol, D-sphingosine, L-glutamic acid, L-methionine, L-proline, linoleic acid, malic acid, malonic acid, oleic acid, palmitic acid, salicylic acid, succinic acid, urea, uric acid, xanthine
	Apoptosis of pancreatic cancer cell lines	5.88E-05	Increased	2.073	7	arachidonic acid, cholesterol, GABA, linoleic acid, oleic acid, palmitic acid, stearic acid
	Cell death of pancreatic cancer cell lines	2.15E-05	Increased	2.073	8	arachidonic acid, cholesterol, GABA, lactic acid, linoleic acid, oleic acid, palmitic acid, stearic acid
	Quantity of blood cells	7.26E-04	Increased	2.111	14	arachidonic acid, ascorbic acid, cholesterol, GABA, L-methionine, L-phenylalanine, lactic acid, lauric acid, linolenic acid, niacinamide, oleic acid, palmitic acid, salicylic acid, uric acid
	Ion homeostasis of cells	1.22E-02	Increased	2.122	16	adenine, adenosine, ascorbic acid, beta-alanine, cholesterol, GABA, glucose-6-phosphate, glycine, L-glutamic acid, niacinamide, oleic acid, palmitic acid, putrescine, retinaldehyde, salicylic acid, uric acid

a. Diseases & Functions

**WD/WD-V vs. LD/LD-V**	Cell death of phagocytes	7.52E-03	Increased	2.128	10	ascorbic acid, cholesterol, glycine, L-glutamic acid, niacinamide, palmitic acid, salicylic acid, sorbitol, stearic acid, uric acid
	Concentration of eicosanoid	5.30E-04	Increased	2.159	10	adenosine, arachidonic acid, ascorbic acid, GABA, linoleic acid, linolenic acid, nicotinic acid, oleic acid, palmitic acid, salicylic acid
	Apoptosis of tumor cell lines	9.58E-08	Increased	2.167	29	adenosine, arachidonic acid, ascorbic acid, cholesterol, citric acid, D-sphingosine, ethanolamine, GABA, L-cystine, L-glutamic acid, L-methionine, L-phenylalanine, L-serine, L-tyrosine, linoleic acid, malonic acid, myristic acid, oleic acid, orotic acid, palmitic acid, phenylacetic acid, phosphorylethanolamine, pyruvic acid, salicylic acid, sorbitol, stearic acid, sucrose, uridine, xanthine
	Killing of cells	3.53E-05	Increased	2.182	13	adenosine, arachidonic acid, ascorbic acid, cholesterol, glycine, L-glutamic acid, lauric acid, linoleic acid, myristic acid, niacinamide, oleic acid, palmitic acid, salicylic acid
	Proliferation of embryonic cell lines	1.09E-03	Increased	2.183	7	adenosine, GABA, guanosine, L-threonine, salicylic acid, sorbitol, uridine
	Accumulation of acylglycerol	3.66E-07	Increased	2.193	12	arachidonic acid, cholesterol, cholic acid, glycerol, L-cysteine, lauric acid, linoleic acid, myristic acid, niacinamide, oleic acid, palmitic acid, stearic acid
	Secretion of molecule	9.35E-03	Increased	2.204	14	adenosine, arachidonic acid, ascorbic acid, cholesterol, D-sphingosine, GABA, L-glutamic acid, L-methionine, linoleic acid, nicotinic acid, oleic acid, palmitic acid, salicylic acid, succinic acid
	Quantity of monosaccharide	3.45E-05	Increased	2.208	16	AMP, ascorbic acid, cholic acid, glycerol, glycine, L-cysteine, L-glutamine, L-ornithine, lactic acid, levan, niacinamide, oleic acid, pyruvic acid, salicylic acid, sucrose, uric acid
	Concentration of cholesterol ester	2.46E-04	Increased	2.213	6	cholesterol, cholic acid, linoleic acid, oleic acid, palmitic acid, phytol
	Dysfunction of mitochondria	3.59E-03	Increased	2.213	8	arachidonic acid, benzoic acid, cholesterol, L-glutamic acid, malonic acid, palmitic acid, salicylic acid, uric acid
	Growth of intestine	1.06E-03	Increased	2.219	5	adenosine, guanosine, L-glutamic acid, putrescine, uridine
	Gluconeogenesis of hepatocytes	4.94E-06	Increased	2.219	8	citrulline, glycerol, L-alanine, L-glutamine, lactic acid, oleic acid, pyruvic acid, retinaldehyde
	Cell viability of lung cancer cells	7.31E-06	Increased	2.219	5	adenosine, guanosine, inosine, L-glutamic acid, uridine
	Cell death of antigen presenting cells	1.70E-03	Increased	2.232	9	ascorbic acid, cholesterol, glycine, L-glutamic acid, niacinamide, palmitic acid, salicylic acid, stearic acid, uric acid
	Apoptosis	1.38E-06	Increased	2.233	43	guanosine, L-cysteine, L-cystine, L-glutamic acid, L-glutamine, L-methionine, L-phenylalanine, L-serine, L-tyrosine, lactic acid, lauric acid, linoleic acid, linolenic acid, malic acid, malonic acid, myristic acid, niacinamide, oleic acid, orotic acid, palmitic acid, phenylacetic acid, phosphorylethanolamine, putrescine, pyruvic acid, salicylic acid, sorbitol, stearic acid, succinic acid, sucrose, uric acid, uridine, xanthine
	Replication of Junin virus strain Candid 1	6.10E-05	Increased	2.236	5	adenine, adenosine, guanosine, hypoxanthine, inosine
	Replication of viral replicon	1.30E-04	Increased	2.236	5	adenine, adenosine, guanosine, hypoxanthine, inosine
	Replication of Tacaribe virus strain TRVL11573	2.46E-04	Increased	2.236	5	adenine, adenosine, guanosine, hypoxanthine, inosine
	Transport of monovalent inorganic cation	1.94E-03	Increased	2.236	6	L-glutamic acid, linoleic acid, linolenic acid, oleic acid, palmitic acid, uric acid
	Cell death of cervical cancer cell lines	1.09E-02	Increased	2.236	6	adenosine, AMP, arachidonic acid, cholesterol, citric acid, D-sphingosine
	Consumption of oxygen	1.59E-04	Increased	2.243	11	adenosine, AMP, ascorbic acid, L-glutamic acid, lactic acid, malic acid, oleic acid, palmitic acid, pyruvic acid, salicylic acid, succinic acid
	Quantity of polyunsaturated fatty acids	3.81E-04	Increased	2.258	11	adenosine, arachidonic acid, ascorbic acid, cholesterol, GABA, linoleic acid, linolenic acid, nicotinic acid, oleic acid, palmitic acid, salicylic acid
	Accumulation of lipid	1.72E-05	Increased	2.306	19	adenosine, arachidonic acid, cholesterol, cholic acid, glycerol, inositol, itaconic acid, L-cysteine, L-serine, lauric acid, linoleic acid, myristic acid, niacinamide, nicotinic acid, oleic acid, orotic acid, palmitic acid, stearic acid, uridine

a. Diseases & Functions

**WD/WD-V vs. LD/LD-V**	Metabolism of reactive oxygen species	4.09E-04	Increased	2.346	30	adenosine, arachidic acid, arachidonic acid, ascorbic acid, cholesterol, D-sphingosine, glucose-6-phosphate, glycine, guanosine, L-cysteine, L-glutamic acid, L-methionine, L-proline, L-tryptophan, lactic acid, linoleic acid, malic acid, malonic acid, niacinamide, oleic acid, palmitic acid, pyruvic acid, retinaldehyde, salicylic acid, sorbitol, succinic acid, urea, uric acid, uridine, xanthine
	Cell death of macrophages	5.07E-03	Increased	2.376	7	cholesterol, L-glutamic acid, niacinamide, palmitic acid, salicylic acid, stearic acid, uric acid
	Hyperpolarization	7.59E-03	Increased	2.393	6	adenosine, GABA, glycine, L-glutamic acid, pyruvic acid, succinic acid
	Respiration of mitochondria	6.24E-03	Increased	2.407	6	hypoxanthine, inosine, L-glutamic acid, malic acid, pyruvic acid, succinic acid
	Apoptosis of stem cells	4.08E-03	Increased	2.415	6	adenosine, ascorbic acid, D-sphingosine, palmitic acid, sorbitol, uric acid
	Metabolism of hydrogen peroxide	1.81E-04	Increased	2.424	13	arachidonic acid, ascorbic acid, cholesterol, glycine, L-glutamic acid, L-tryptophan, malic acid, niacinamide, oleic acid, palmitic acid, pyruvic acid, succinic acid, urea
	Cell death of myeloid cells	1.00E-02	Increased	2.432	9	ascorbic acid, cholesterol, L-glutamic acid, niacinamide, palmitic acid, salicylic acid, sorbitol, stearic acid, uric acid
	Replication of arenaviridae	5.49E-05	Increased	2.449	6	adenine, adenosine, cholesterol, guanosine, hypoxanthine, inosine
	Excitation of orexin neurons	2.90E-05	Increased	2.449	6	glycine, L-aspartic acid, L-cysteine, L-proline, L-serine, lactic acid
	Proliferation of CD4+ T-lymphocytes	1.47E-03	Increased	2.449	6	cholesterol, hypoxanthine, linoleic acid, oleic acid, palmitic acid, stearic acid
	Synthesis of reactive oxygen species	8.26E-04	Increased	2.501	29	adenosine, arachidic acid, arachidonic acid, ascorbic acid, cholesterol, D-sphingosine, glucose-6-phosphate, glycine, guanosine, L-cysteine, L-glutamic acid, L-methionine, L-proline, lactic acid, linoleic acid, malic acid, malonic acid, niacinamide, oleic acid, palmitic acid, pyruvic acid, retinaldehyde, salicylic acid, sorbitol, succinic acid, urea, uric acid, uridine, xanthine
	Quantity of cells	3.49E-05	Increased	2.503	22	adenosine, arachidonic acid, ascorbic acid, cholesterol, citric acid, GABA, inosine, L-glutamic acid, L-methionine, L-phenylalanine, lactic acid, lauric acid, linoleic acid, linolenic acid, malonic acid, niacinamide, nicotinic acid, oleic acid, palmitic acid, salicylic acid, sucrose, uric acid
	Gluconeogenesis	7.83E-06	Increased	2.56	9	citrulline, glycerol, L-alanine, L-glutamine, lactic acid, oleic acid, palmitic acid, pyruvic acid, retinaldehyde
	Response of chorda tympani	3.19E-06	Increased	2.564	7	citric acid, L-glutamic acid, linoleic acid, linolenic acid, maltose, oleic acid, sucrose
	Killing of bacteria	7.02E-04	Increased	2.607	8	arachidonic acid, ascorbic acid, lauric acid, linoleic acid, myristic acid, niacinamide, palmitic acid, salicylic acid
	Response of glossopharyngeal nerve	1.72E-07	Increased	2.613	7	citric acid, L-glutamic acid, lauric acid, linoleic acid, linolenic acid, oleic acid, sucrose
	Entry into S phase of hepatocytes	5.36E-09	Increased	2.646	7	glycine, L-alanine, L-asparagine, L-aspartic acid, L-glutamic acid, L-proline, L-serine
	Efflux of L-alanine	3.19E-06	Increased	2.646	7	beta-alanine, glycine, L-alanine, L-cysteine, L-methionine, L-serine, L-threonine
	Export of molecule	5.47E-10	Increased	2.68	25	adenosine, arachidonic acid, benzoic acid, beta-alanine, cholesterol, cholic acid, GABA, glycine, L-alanine, L-aspartic acid, L-cysteine, L-glutamic acid, L-leucine, L-methionine, L-serine, L-threonine, linoleic acid, malonic acid, nicotinic acid, oleic acid, palmitic acid, putrescine, pyruvic acid, salicylic acid, uric acid
	Biosynthesis of hydrogen peroxide	4.44E-04	Increased	2.701	12	arachidonic acid, ascorbic acid, cholesterol, glycine, L-glutamic acid, malic acid, niacinamide, oleic acid, palmitic acid, pyruvic acid, succinic acid, urea
	Production of reactive oxygen species	2.53E-03	Increased	2.761	23	arachidonic acid, ascorbic acid, cholesterol, D-sphingosine, glucose-6-phosphate,guanosine, L-cysteine, L-glutamic acid, L-methionine, lactic acid, linoleic acid, malic acid, malonic acid, niacinamide, oleic acid, palmitic acid,pyruvic acid, retinaldehyde, salicylic acid, sorbitol, succinic acid, uric acid, xanthine
	Efflux of neutral amino acid	3.29E-07	Increased	2.772	8	beta-alanine, glycine, L-alanine, L-cysteine, L-leucine, L-methionine, L-serine, L-threonine
	Production of hydrogen peroxide	1.60E-03	Increased	2.779	10	arachidonic acid, ascorbic acid, cholesterol, L-glutamic acid, malic acid, niacinamide, oleic acid, palmitic acid, pyruvic acid, succinic acid

a. Diseases & Functions

WD/WD-V vs. LD/LD-V	Excitation of neurons	5.62E-04	Increased	2.822	8	GABA, glycine, L-aspartic acid, L-cysteine, L-glutamic acid, L-proline, L-serine, lactic acid
	S phase	7.83E-06	Increased	2.828	9	citric acid, glycine, L-alanine, L-asparagine, L-aspartic acid, L-glutamic acid, L-proline, L-serine, uridine
	Interphase	1.04E-06	Increased	2.926	17	arachidonic acid, cholesterol, citric acid, glycine, L-alanine, L-asparagine, L-aspartic acid, L-glutamic acid, L-proline, L-serine, linoleic acid, linolenic acid, niacinamide, phosphorylethanolamine, salicylic acid, stearic acid, uridine
	Efflux of L-amino acid	1.66E-08	Increased	2.945	11	adenosine, benzoic acid, beta-alanine, glycine, L-alanine, L-cysteine, L-leucine, L-methionine, L-serine, L-threonine, pyruvic acid
	Quantity of carbohydrate	2.98E-06	Increased	3.25	23	adenosine, AMP, ascorbic acid, beta-glycerophosphoric acid, cholesterol, cholic acid, citrulline, glycerol, glycine, L-cysteine, L-glutamine, L-ornithine, lactic acid, levan, N-acetyl-D-mannosamine, niacinamide, oleic acid, palmitic acid, pyruvic acid, salicylic acid, sucrose, uric acid, uridine
	Stimulation of cells	3.48E-04	Increased	3.527	13	arachidonic acid, ascorbic acid, GABA, glycine, L-aspartic acid, L-cysteine, L-glutamic acid, L-proline, L-serine, L-tryptophan, lactic acid, palmitic acid, uric acid
	Quantity of Ca2+	1.22E-08	Increased	3.65	30	9Z-hexadecenoic acid, adenine, adenosine, arachidic acid, arachidonic acid, behenic acid, beta-glycerophosphoric acid, cholesterol, citric acid, D-sphingosine, GABA, glycine, heptadecanoic acid, L-cysteine, L-glutamic acid, L-lysine, L-ornithine, lauric acid, linoleic acid, linolenic acid, myristic acid, nonadecanoic acid, oleic acid, palmitic acid, pentadecanoic acid, pyruvic acid, retinaldehyde, stearic acid, succinic acid, xanthine
	Quantity of metal ion	6.43E-09	Increased	3.673	32	9Z-hexadecenoic acid, adenine, adenosine, arachidic acid, arachidonic acid, behenic acid, beta-glycerophosphoric acid, cholesterol, citric acid, D-sphingosine, erythritol, GABA, glycine, heptadecanoic acid, L-cysteine, L-glutamic acid, L-lysine, L-ornithine, lauric acid, linoleic acid, linolenic acid, myristic acid, nonadecanoic acid, oleic acid, palmitic acid, pentadecanoic acid, pyruvic acid, retinaldehyde, stearic acid, succinic acid, uric acid, xanthine
	Organismal death	7.02E-10	Increased	4.466	38	4-hydroxybenzoic acid, adenosine, aniline, ascorbic acid, benzoic acid, caprolactam, cetyl alcohol, citric acid, creatinine, D-pantothenic acid, D-xylose, ethanolamine, fumaric acid, glycerol, glycolic acid, L-cysteine, L-glutamine, L-phenylalanine, L-tryptophan, lactic acid, lauric acid, linoleic acid, malic acid, myristic acid, niacinamide, nicotinic acid, oleic acid, orotic acid, palmitic acid, phosphoric acid, phthalic acid, salicylic acid, sorbitol, stearic acid, succinic acid, sucrose, terephthalic acid, urea

b. Canonical Pathways

Groups	Canonical Pathway	-log(p-value)	Ratio	z-score	Molecules
WD/LD-V vs. WD/LD-T	tRNA Charging	1.14E+01	4.42E-01	-3.900	AMP, glycine, L-alanine, L-asparagine, L-aspartic acid, L-cysteine, L-glutamic acid, L-glutamine, L-isoleucine, L-leucine, L-lysine, L-methionine, L-phenylalanine, L-proline, L-serine, L-threonine, L-tryptophan, L-tyrosine, L-valine
	Citrulline Biosynthesis	3.06E+00	3.33E-01	-2.000	citrulline, L-glutamic acid, L-glutamine, L-ornithine, L-proline, urea
LD/LD-V vs. WD/WD-V	tRNA Charging	2.83E-12	4.42E-01	3.900	AMP, glycine, L-alanine, L-asparagine, L-aspartic acid, L-cysteine, L-glutamic acid, L-glutamine, L-isoleucine, L-leucine, L-lysine, L-methionine, L-phenylalanine, L-proline, L-serine, L-threonine, L-tryptophan, L-tyrosine, L-valine
